# Population genomics reveals a long-term increase in azole resistance associated with the emerging cluster AZR among *Candida tropicalis* causing invasive infections

**DOI:** 10.1080/22221751.2026.2695535

**Published:** 2026-06-25

**Authors:** Yiying Zhao, Rongchen Dai, Feiyi Liu, Keyu Liu, Wei Kang, Ge Zhang, Jin Li, Jingjia Zhang, Tong Wang, Haotian Gao, Yuyan Huang, David S. Perlin, Meng Xiao, Yingchun Xu, Xin Fan

**Affiliations:** a Department of Laboratory Medicine, State Key Laboratory of Complex Severe and Rare Diseases, Peking Union Medical College Hospital, Chinese Academy of Medical Sciences and Peking Union Medical College, Beijing, People’s Republic of China; b Clinical Biobank, National Infrastructures for Translational Medicine, Institute of Clinical Medicine, Peking Union Medical College Hospital, Chinese Academy of Medical Sciences and Peking Union Medical College, Beijing, People’s Republic of China; c Center for Discovery and Innovation, Hackensack Meridian Health, Nutley, NJ, USA; d Department of Infectious Diseases and Clinical Microbiology, Beijing Institute of Respiratory Medicine and Beijing Chao-Yang Hospital, Capital Medical University, Beijing, People’s Republic of China; e Beijing Key Laboratory of Multimodal Intelligent Diagnosis and Treatment System for Respiratory Diseases, Beijing Institute of Respiratory Medicine and Beijing Chao-Yang Hospital, Capital Medical University, Beijing, People’s Republic of China

**Keywords:** Candidemia, whole genome sequencing, antifungal resistance, strain replacement, dual-clade mixed infection, China Hospital Invasive Fungal Surveillance Net (CHIF-NET)

## Abstract

A notable increase in azole resistance among *Candida tropicalis* has been observed worldwide, particularly in the Asia-Pacific region, associated with the expansion of an emerging resistant population named cluster AZR. Here, we present up-to-date epidemiological, antifungal susceptibility and population genomic data from the China Hospital Invasive Fungal Surveillance Net (CHIF-NET) study. A total of 911 *C. tropicalis* isolates causing invasive candidiasis were collected in 2022–2023, representing 16.1% of all *Candida* isolates and ranking as the third most common species. High resistance rates to fluconazole (42.3%) were observed, with 87.5% showing cross-resistance to voriconazole. Candidemia isolates showed significantly higher azole resistance than non-candidemia isolates (46.1% vs 39.2%, *p* = 0.036). Fluconazole resistance rate was higher in medical departments (55.0%) but lower in ICUs (33.7%). Longitudinal analysis demonstrated an overall upward trend in fluconazole resistance from 5.7% since 2009. Although a transient decline was observed during 2020–2021, resistance rebounded thereafter and reached a historical peak of 43.1% in 2022. Population genomic analysis of 604 isolates identified cluster AZR as the predominant fluconazole-resistant population (58.6%), and its expansion was associated with the temporal increase in azole resistance. Of note, all cluster AZR isolates harboured the *ERG11* A395 T key resistant mutation, and 98.2% had increased *ERG11* copy number. Besides, genomic analysis identified strain replacement and dual-clade mixed infection in cluster AZR-associated clinical cases. In conclusion, the high prevalence of azole resistance in *C. tropicalis* and the expansion of cluster AZR represent a significant clinical challenge and underscore the need for effective interventions.

## Introduction

Invasive candidiasis has become increasingly prevalent and is associated with mortality rates exceeding 50%, leading to prolonged hospitalization and additional financial costs [1–3]. Among non-*albicans Candida* species, the opportunistic pathogen *Candida tropicalis* represents a global threat, ranking as the first to second most common frequently isolated *Candida* species in Asia and Latin America [4–6]. A notable increase in azole resistance, reaching up to 40%−80%, has been reported among *C. tropicalis* isolates worldwide, with the highest burden observed in the Asia-Pacific region [1,6]. Its significant azole resistance, together with virulence factors including biofilm formation, tissue invasion, and immune evasion, contributes to the higher overall mortality associated with *C. tropicalis* infections compared with other *Candida* species [7]. This species has been classified in high priority group of the World Health Organization (WHO) fungal priority pathogens list (FPPL), underscoring the urgent need for both surveillance and mechanism studies [8].

The common molecular mechanisms underlying azole resistance include mutations and overexpression of the drug target gene *ERG11*, which encodes lanosterol 14α-demethylase (cytochrome P450 CYP51), and upregulation of drug efflux pumps (e.g. CDR and MDR transporters) [7]. Among these mechanisms, the *ERG11* mutation A395 T, leading to the amino acid substitution Y132F, represents one of the most common resistance mechanisms identified in *C. tropicalis* [9,10]. Notably, mutations at this key position have also been identified across multiple *Candida* species [11–13]. Of note, previous studies have identified a shift of the predominant azole-resistant population from multilocus sequence typing (MLST) clade 5 to clade 4 [10,14,15]*.* Moreover, population genomic studies revealed that the dramatic increase of azole resistance in the Asia-Pacific region was largely driven by the emergence and expansion of a subpopulation of MLST clade 4, referred to as cluster AZR or clade X [14,16]. Isolates from this cluster exhibited high-level fluconazole resistance, which was attributed to copy number variations (CNVs) of the *ERG11* gene simultaneously carrying A395 T mutation.

As one of the regions with the highest burden of azole-resistant *C. tropicalis*, there has been a continuing need for surveillance programmes to monitor the antifungal susceptibility dynamics. In 2009, our team initiated the China Hospital Invasive Fungal Surveillance Net (CHIF-NET) to provide informative data on nationwide epidemiology and antifungal susceptibility of pathogens causing invasive fungal diseases (IFDs) [17]. After years of continuous increase, azole resistance declined for the first time in CHIF-NET20-21, marking a temporary reversal of the previous upward trend [18,19]. Here, we presented the up-to-date epidemiological and antifungal susceptibility data of *C. tropicalis* isolates from 49 centres in CHIF-NET22-23, in comparison with data from previous years. Notably, following a transient decline in CHIF-NET20-21, azole resistance rebounded in CHIF-NET22-23, reaching the highest level to date (42.3%) [18,19]. In addition, using a large-scale comparative population genomic analysis of 604 *C. tropicalis* isolates selected from the CHIF-NET study, we revealed that cluster AZR remained the predominant azole-resistant population in recent years. In cases involving multiple strains isolated from individual patients, genomic analysis revealed dual-clade mixed infection or strain replacement between cluster AZR and other phylogenetic populations, highlighting the complex nature of invasive *C. tropicalis* infections.

## Material and methods

### C. tropicalis isolates

The CHIF-NET study is a laboratory-based, multicentre passive surveillance study of invasive yeast infections, which was initiated in 2009 [17]. This study included the most recent two-year data from 2022 to 2023 (CHIF-NET22-23), collected from a total of 49 hospitals (44 and 46 participating hospitals in CHIF-NET22 and CHIF-NET23, respectively) in 28 provinces across China. The study inclusion criteria were as previously described [17].

For each surveillance year, all isolates from eligible patients with IFDs, including candidemia patients, were forwarded to a central laboratory (Peking Union Medical College Hospital), for confirmatory species identification and antifungal susceptibility testing. Related processing procedures were as previously described [20]. A total of 911 *C. tropicalis* isolates causing IFDs were included in this study.

To gain a more comprehensive understanding of longitudinal changes, we compared the epidemiological and antifungal susceptibility data from CHIF-NET22-23 with those from earlier surveillance years. In addition, population genomic analysis was performed on 604 *C. tropicalis* strains collected between 2009 and 2023, including 448 strains described in our previous study [14] and 156 strains sequenced in the present study (Supplementary Table S1 and Figure S1). Specifically, the newly sequenced 156 isolates were selected from the CHIF-NET collection during 2020–2023 using a stratified random sampling strategy, with approximately 30% fluconazole-resistant isolates. Within each susceptibility category, isolates were randomly selected without additional stratification. After sequencing quality control, and data filtering, 156 isolates were retained for downstream analyses.

### Species identification

All isolates were identified to the species level in the central laboratory by matrix-assisted laser desorption/ionization time-of-flight mass spectrometry (MALDI-TOF MS) with Autof MS 1000 (Autobio Diagnostics, Zhengzhou, China) or Vitek MS (bioMérieux, Marcy-l’Étoile, France) system. MALDI-TOF MS identification was interpreted according to the manufacturers’ instructions. For isolates with no identification or uncertain identification (e.g. low-confidence value) results by MALDI-TOF MS, sequencing of the fungal internal transcribed spacer (ITS) rDNA regions was performed for definitive species identification [17,21]. For ITS sequence-based identification, species-level identification was accepted when the best BLAST hit showed ≥99% sequence identity to a validated type strain’s sequence.

### Antifungal susceptibility testing

The *in vitro* susceptibility to nine antifungal agents, including fluconazole (FLU), voriconazole (VOR), itraconazole (IZ), posaconazole (PZ), caspofungin (CAS), micafungin (MF), anidulafungin (AND), 5-flucytosine (FC), and amphotericin B (AMB), was determined using Sensititre YeastOne^TM^ YO10 method (Thermo Fisher Scientific, Cleveland, OH, USA) following manufacturer’s instructions [22]. *Candida parapsilosis* ATCC 22019 and *Candida krusei* ATCC 6258 were included as quality control strains in each batch of antifungal susceptibility testing, and all minimum inhibitory concentrations (MICs) remained within the recommended quality control ranges throughout the surveillance period [23]. For FLU, VOR, CAS, MF, and AND, antifungal susceptibility results were interpreted per latest CLSI clinical breakpoints (CBPs); while for IZ, PZ, and AMB that CBPs were currently not available, epidemiological cutoff values (ECVs) were used to distinguish wild-type (WT) from non-wild-type (NWT) phenotypes (Supplementary Table S2) [23,24]. Cross-resistance was defined as resistance or of non-wild-type phenotype to ≥2 antifungal agents within the same class, whereas multidrug resistance was defined as resistance or of NWT phenotype to antifungal agents from ≥2 classes [20].

### Whole-genome sequencing (WGS)

All *C. tropicalis* isolates for WGS were grown on Sabouraud Dextrose Agar (SDA; Oxoid, Thermo Fisher Scientific, CM0041 T) at 35 °C for 48 h, and subjected to short-read sequencing. Genomic DNA extraction was performed using the fungal DNA extraction kit (magnetic beads) (Majorbio, Shanghai, China) according to manufacturer’s protocol. Purified genomic DNA was quantified, and high-quality DNA was used for subsequent analyses. A paired-end library with an average insert size of 400 bp was prepared using the NEXTFLEX Rapid DNA-Seq Kit (Revvity, Waltham, MA, USA), and sequenced on the Illumina NovaSeq™ X Plus (Illumina, San Diego, CA, USA) at Majorbio Bio-pharm Technology Co., Ltd. (Shanghai, China). Raw reads were filtered using fastp with the default argument (version 0.20.0) [25].

### Read mapping, variant identification and filtering

For *C. tropicalis* bioinformatic analyses, an in-house pipeline (designated “Spica”) was developed and applied [14]. Briefly, all subsequent analyses were performed using this pipeline. Filtered reads of each isolate were mapped to the *C. tropicalis* MYA-3404 reference genome (GenBank assembly accession: GCA_013177555.1 [https://www.ncbi.nlm.nih.gov/datasets/genome/GCA_013177555.1/]) using the Burrows-Wheeler alignment tool (BWA) version 0.7.7 with the BWA-MEM algorithm [26]. BAM files were generated and sorted using SAMtools (version 1.6) with the default argument [27]. SNP calling was performed with the Genome Analysis Toolkit (GATK) (version 4.3.0.0) according to the GATK Best Practices [28,29]. For each sample, reads were marked using MarkDuplicates, and variants were called using HaplotypeCaller to create single-sample GVCF files. Individual GVCF files were combined using CombineGVCFs with default parameters, followed by joint genotyping of all isolates using GenotypeGVCFs. Finally, SNPs were selected using SelectVariants and filtered based on the following parameters: QD <2.0; FS >60.0; MQ <40.0; MQRankSum <−12.5; ReadPosRankSum <−8.0; and HaplotypeScore >13.0. INDELs were selected using SelectVariants and filtered based on the following parameters: QD <2.0; QUAL <30.0; FS >200.0; and ReadPosRankSum <−20.0. A “bamdst” script (https://github.com/shiquan/bamdst) was used for quality control based on BAM files, and samples with a low fraction of reads mapped (<60%) were excluded from subsequent analyses. Because *C. tropicalis* is a diploid species, an average read depth of ≥60× was considered sufficient to ensure high level of confidence for downstream analyses.

### Multilocus sequence typing (MLST) analysis

MLST analysis was conducted using six gene loci as previously described [30]. For five of the six loci (*ICL1*, *MDR1*, *SAPT2*, *SAPT4*, and *ZWF1a*), SNPs within the reference regions were extracted directly from VCF files. For the *XYR1* locus, reads were mapped to a single copy of reference sequence to identify SNPs. The DNA sequences of each locus were reconstructed using a custom script and compared with data from the PubMLST database (https://pubmlst.org, last accessed on 1 December 2025) to determine allele numbers and diploid sequence types (DSTs). Novel DSTs identified in this study were numbered from DST12001. The MLST allelic profiles were analyzed by goeBURST implemented in PHYLOViZ (version 2.0) software to define clonal complexes (CCs), starting from CCN0 and named in descending order according to the number of isolates within each CC [31].

### Phylogenetic and population structure analysis

High-quality SNPs were obtained after GATK hard filtering as described above. SNPs from the joint genotyped VCF file were converted to FASTA format using vcf2phylip.py (https://github.com/edgardomortiz/vcf2phylip), with heterozygous SNP loci converted as IUPAC bases. A maximum-likelihood (ML) tree was inferred using IQ-TREE (version 1.6.12) with 1000 ultrafast bootstrap replicates [32]. The best-fit nucleotide substitution model selected by ModelFinder according to Bayesian Information Criterion (BIC) was TVM + F + I + G4, and phylogenetic clustering was supported by bootstrap values >99.0% [33,34]. The tree was visualized and annotated using iTOL (https://itol.embl.de).

Cluster AZR and group AZR-ADJ were defined per described in our previous study [14]. Briefly, pairwise genome-wide SNP distances were calculated from the quality-filtered SNP matrix. Candidate isolates belonging to cluster AZR were initially screened using a pairwise genome-wide SNP distance threshold of ≤20000 SNPs together with highly supported phylogenetic clustering. Additionally, average nucleotide identity (ANI) was calculated using FastANI (version 1.33) based on quality-controlled genome assemblies, and an intra-group ANI cutoff value of ≥99.6% was applied to support WGS cluster assignment. Whole-genome SNP-based BIC and ADMIXTURE (version 1.3) analyses were performed to estimate the optimal number of genomic populations. Principal components analysis (PCA) and discriminant analysis of principal components (DAPC) were further conducted using GCTA (version 1.94) and PLINK (version 1.9) to validate the inferred population structure. Final assignment of isolates to cluster AZR was determined based on concordant evidence from ML phylogeny, pairwise SNP distances, ANI clustering, and population structure analyses.

### Analysis of polyploidy, aneuploidy, large structure variation, copy number variation (CNV) and loss of heterozygosity (LOH)

Polyploidy was assessed based on the frequency distribution of cumulative heterozygous biallelic SNPs across all scaffolds as described previously [14]. Aneuploidy, large structure variation and CNV were analyzed using “Splint” algorithm, which processed BAM files with 500-bp window frames [35]. To identify CNVs in drug target genes, sequencing depth was calculated from sorted BAM files using the SAMtools “depth” command. The mean read depth across non-overlapping 1-kb windows spanning the whole genome was visualized using a custom script for assisting analysis of aneuploidy and large structure variation events.

LOH analysis was carried out as previously described [14]. Briefly, the genetic state of each locus in each sample was coded to distinguish loci that were homozygous for the haploid reference (−1), heterozygous SNPs (0), and homozygous nonreference SNPs (1). The genome was divided into consecutive 10-kb windows, and the number of heterozygous SNPs within each window was calculated. A genome-wide LOH matrix encompassing all isolates from the VCF dataset was subsequently generated and visualized using custom scripts.

### Classification of infection episodes into strain replacement and dual-clade mixed infection

Multiple isolates from individual patients within single infection episodes (less than one month) that demonstrated antifungal susceptibility shifts were selected for further analysis. WGS and downstream analyses were performed as described above. Genetically distinct strains were determined based on assignment to different WGS-defined phylogenetic clusters (see *phylogenetic and population structure analysis* above). Strain replacement was defined as the sequential isolation of strains belonging to different phylogenetic clusters from the same patient over time. Dual-clade mixed infection was defined as the presence of ≥2 strains belonging to different phylogenetic clusters from the same patient and specimen source at the same sampling time point [36,37].

### Statistical analysis

All statistical analyses were performed using IBM SPSS software (version 27.0; IBM SPSS Corp., New York, USA) and R (version 4.4.2). Categorical variables were compared using the *χ*^2^ or Fisher’s exact test, as appropriate. For categorical variables involving multiple group comparisons, post-hoc pairwise comparisons were performed using pairwise proportion tests with Bonferroni correction. Multivariable logistic regression analysis was performed to identify independent factors, as appropriate. Temporal trends were evaluated using the Cochran-Armitage trend test, with exact binomial 95% confidence intervals (CIs) calculated where appropriate. A *p*-value < 0.05 was considered significant.

## Results

### Clinical characteristics of C. tropicalis isolates

From 2022 to 2023, a total of 911 nonduplicate *C. tropicalis* isolates recovered from patients with IFDs were collected, accounting for 16.1% of all *Candida* isolates. Data on the distribution of isolates by clinical characteristics was summarized in Table 1. Isolates from outpatient/emergency departments only comprised 9.5% (87/911) of the collection, while isolates from inpatient wards accounted for 90.5% (824/911), including 31.6% from intensive care units (ICUs) (288/911), 28.5% from medical departments (260/911), and 25.5% from surgical departments (232/911), with the remaining from other inpatient wards (4.8%, 44/911). Among all specimen types, isolates recovered from blood cultures comprised 44.8% of the collection (408/911) (Table 1).

**Table 1.** Distribution of *Candida tropicalis* isolates from invasive candidiasis by clinical service and specimen sources from CHIF-NET22-23.

Characteristics	Overall, % (n)	FLU-R, % (n)	AZR, % (n/N)
**Overall**	**100** (**911)**	**42.3** (**385)**	**58.6** (**109/186)**
**Clinical services**			
ICU	31.6 (288)	33.7 (97)	45.5 (25/55)
Medical	28.5 (260)	55.0 (143)	66.7 (42/63)
Surgical	25.5 (232)	38.8 (90)	71.4 (35/49)
Outpatient/Emergency	9.5 (87)	41.4 (36)	30.8 (4/13)
Other wards	4.8 (44)	43.2 (19)	50.0 (3/6)
**Specimen types**			
Blood	44.8 (408)	46.1 (188)	60.0 (72/120)
Non-blood	55.2 (503)	39.2 (197)	56.1 (37/66)

Note: FLU, fluconazole; AZR, cluster AZR; ICU, intensive care unit. FLU-R indicates the proportion of isolates resistant to fluconazole according to CLSI clinical breakpoints. AZR indicates the proportion of isolates belonging to cluster AZR among fluconazole-resistant isolates.

### In vitro susceptibility to azoles

**Figure 1.**
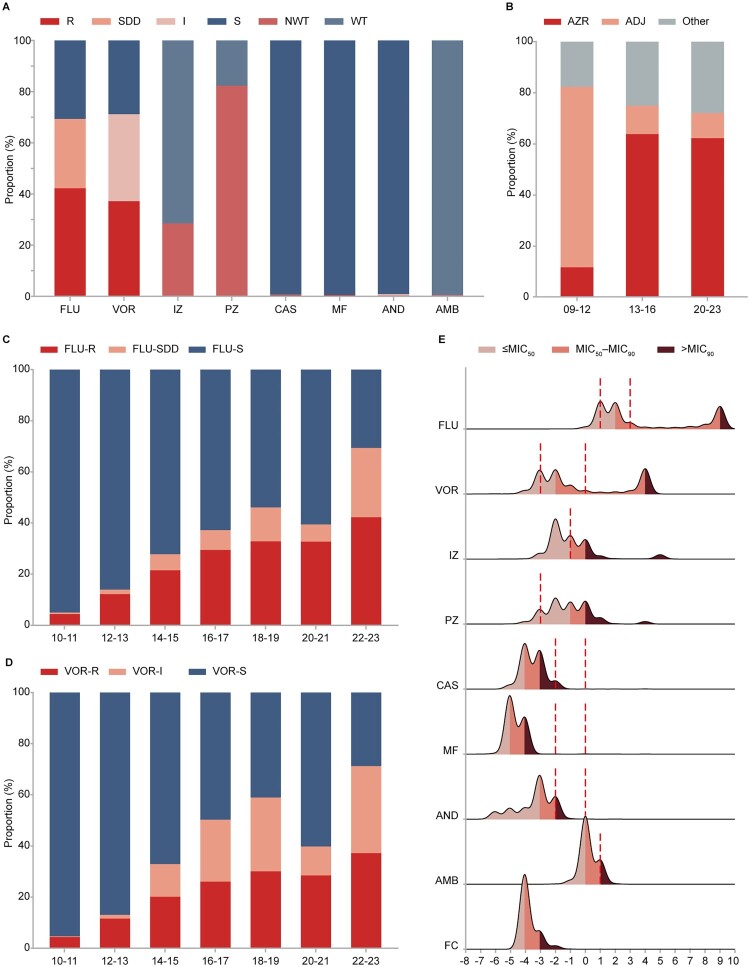
Antifungal susceptibility and temporal trends of azole resistance and phylogenetic populations in *C. tropicalis*. A. Antifungal susceptibility profiles of *C. tropicalis* isolates in CHIF-NET22-23. B. Changes in the proportions of cluster AZR and group AZR-ADJ over time. C, D. Temporal trends in the proportions of resistant, susceptible-dose-dependent and susceptible to fluconazole (C) and voriconazole (D) over a 14-year surveillance period. E. Minimum inhibitory concentrations (MICs) distributions of *C. tropicalis* isolates in CHIF-NET22-23. The x-axis represents log_2_ MICs (μg/mL). Shaded areas represent isolates with MICs ≤ MIC_50_, between MIC_50_ and MIC_90_, and >MIC_90_, respectively. Vertical dashed lines indicate the corresponding CLSI clinical breakpoints (CBPs) or epidemiological cutoff values (ECVs) for each antifungal agent (Supplementary Table S2). Abbreviations: FLU, fluconazole; VOR, voriconazole; IZ, itraconazole; PZ, posaconazole; CAS, caspofungin; MF, micafungin; AND, anidulafungin; AMB, amphotericin B; FC, 5-flucytosine; I, intermediate; R, resistant; SDD, susceptible-dose-dependent; S, susceptible; WT, wild-type; NWT, non-wild-type; MIC, minimum inhibitory concentration; AZR, cluster AZR; ADJ, group AZR-ADJ.

Azole resistance was significantly high among 911 *C. tropicalis* isolates from CHIF-NET22-23, with 42.3% (385/911) of isolates resistant to FLU and 37.2% (339/911) resistant to VOR (Figure 1(A)). In addition, 37.0% (337/911) of strains showed cross-resistance to FLU and VOR. The rates of NWT isolates to IZ and PZ were 28.5% (260/911) and 82.3% (750/911), respectively (Figure 1(A)). Furthermore, 28.2% (257/911) of isolates were cross-resistant to all four tested azoles.

Among different clinical departments, the medical departments showed the highest FLU resistance rate (55.0%, 143/260), which was significantly higher than that in ICUs (33.7%, 97/288) (adjusted *p* < 0.001) (Table 1). Regarding different specimen types, the FLU resistance rate was significantly higher in candidemia isolates (46.1%, 188/408) compared to non-candidemia isolates (39.2%, 197/503) (*p* = 0.036) (Table 1). After multivariable adjustment, the difference in FLU resistance between medical departments and ICUs (*p* < 0.001), surgical departments (*p* = 0.001), and outpatient/emergency departments (*p* = 0.019) remained significant, whereas other pairwise comparisons were not statistically significant (Supplementary Table S3).

**Figure 2.**
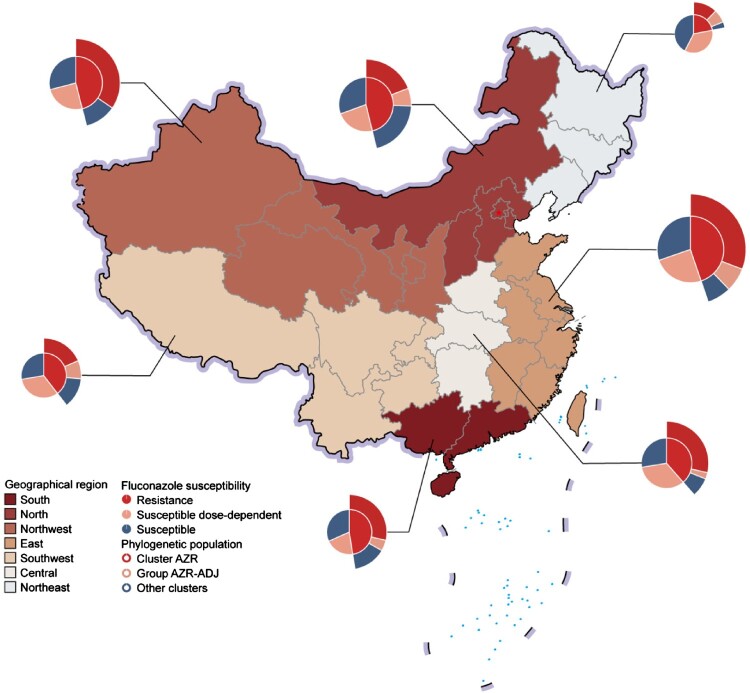
Geographic distribution of azole resistance rates and phylogenetic populations across different regions in China. The colour gradient indicates the overall resistance burden, and pie charts represent antifungal susceptibilities (inner pie charts) and phylogenetic population structure (outer ring charts). The size of each pie chart represents the relative number of isolates collected in each region during 2022–2023.

Across the seven administrative regions in China, variations in FLU resistance rates were noted among *C. tropicalis* isolates (21.9% to 47.5%, overall *p* = 0.01) (Figure 2). Resistance rates were significantly lower in the Northeast (21.9%, 16/73) region than in the South (47.5%, 48/101), North (46.3%, 68/147), Northwest (46.1%, 65/141), and East (44.8%, 99/221) regions (all adjusted *p* < 0.05) (Figure 2). Multivariable logistic regression analysis demonstrated that both geographical region (overall *p* = 0.002) and clinical department (overall *p* < 0.001) were independently associated with FLU resistance, whereas specimen types were not significantly associated with resistance after adjustment (Supplementary Table S3).

### Trends in FLU and VOR resistance over time

Trends in FLU and VOR resistance rates among *C. tropicalis* over 14 surveillance years are shown in Figure 1(C and D). Overall, a notable increase in azole resistance was observed (Cochran-Armitage trend tests, *p* < 0.001), with FLU resistance rate rising from 4.4% (15/340, 95% CI 2.5%−7.2%) to 42.3% (385/911, 95% CI 39.0%−45.5%) and VOR resistance rate from 4.4% (15/340, 95% CI 2.5%−7.2%) to 37.2% (339/911, 95% CI 34.1%−40.4%) (Figure 1(C and D)). The FLU resistance rate continuously increased from CHIF-NET10-11, followed by a transient decrease in CHIF-NET20-21 and a rebound to over 40% in CHIF-NET22-23, exceeding the previous peak observed in CHIF-NET19 (Figure 1(C)) [18,19].

### Population genomics showed the predominance of cluster AZR in azole-resistant population

To gain a genetic overview of recent shifts in azole susceptibility, we performed WGS and population analyses of 604 *C. tropicalis* isolates collected from IFDs patients in China between CHIF-NET09 and CHIF-NET23. Using WGS-based MLST analysis, 238 DSTs that belonged to 129 MLST CCs were identified, including 31 novel DSTs that were not included in PubMLST database (Supplementary Tables S1 and S4). MLST clade 4 was the most prevalent azole-resistant clade in China, comprising 64.5% (120/186) of the collection (Supplementary Table S1).

**Figure 3.**
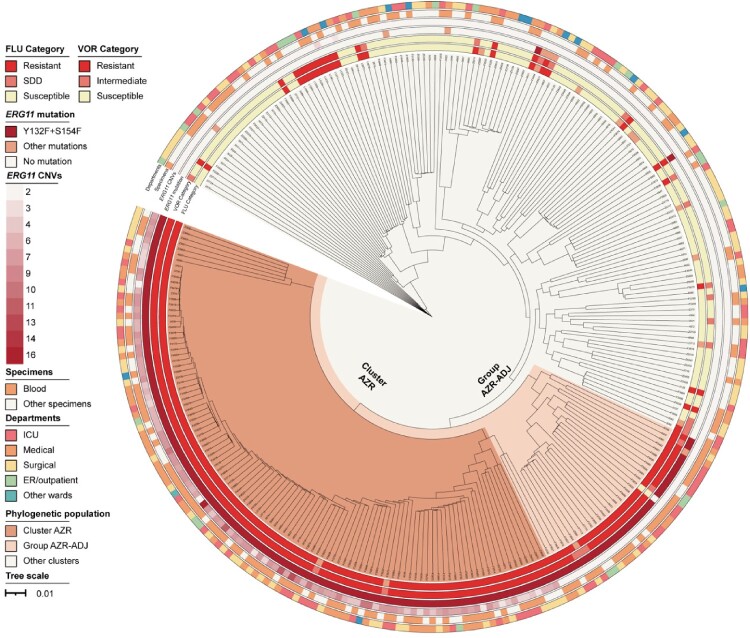
Maximum-likelihood phylogenetic tree of *C. tropicalis* isolates based on whole-genome sequencing (WGS) data. Cluster AZR and group AZR-ADJ are indicated. Outer rings represent fluconazole (FLU) and voriconazole (VOR) susceptibility profiles, *ERG11* key mutations, *ERG11* copy number variations (CNVs), specimen sources, and clinical departments.

By combining phylogenetic, ANI and ADMIXTURE population structure analyses, a ML tree was built and clusters (CTCs) were decided (Figure 3). Among FLU-resistant isolates, 58.6% (109/186) belonged to cluster AZR and 16.1% (30/186) belonged to group AZR-ADJ, which is a branch adjacent to cluster AZR that also exhibited high-level resistance (Figure 3, Supplementary Table S1) [14]. All the cluster AZR isolates belonged to MLST clade 4, whereas only 37.5% (12/32) of the group AZR-ADJ isolates belonged to MLST clade 4 (Supplementary Table S1). All cluster AZR isolates were non-susceptible to FLU, of which 98.2% (109/111) were cross-resistant to FLU and VOR, while the remaining two isolates were susceptible-dose-dependent (SDD) to FLU and intermediate to VOR (Figure 3). In addition, 94.6% (105/111) of cluster AZR isolates exhibited high-level FLU resistance (MICs >128 μg/mL) (Figure 3). WGS analysis identified that all cluster AZR isolates carried A395 T mutations, and 98.2% exhibited increased *ERG11* copy numbers (median 7, IQR 6–8) (Figure 3).

By clinical department, the proportion of cluster AZR was higher in surgical department (71.4%, 35/49) and medical departments (66.7%, 42/63), and lower in ICUs (45.5%, 25/55) (overall *p* = 0.013) (Table 1). However, the association between clinical department and cluster AZR distribution was not statistically significant after multivariable adjustment (Supplementary Table S5). Regarding the specimen types, the proportion of cluster AZR was higher (60.0%, 72/120) in blood samples than in other specimen types (56.1%, 37/66); however, the difference was not statistically significant (*p* > 0.05) (Table 1).

Geographic variation in the distribution of resistant populations across seven geographic regions in China was analyzed. The proportion of cluster AZR ranged from 41.4% to 75.0%, whereas that of group AZR-ADJ ranged from 0% to 30.6%, with no statistically significant differences across regions (all *p* > 0.05) (Figure 2). Compared with the overall prevalence of cluster AZR, the Northwest (75.0%, 6/8), Central (74.2%, 23/31), and East (68.8%, 22/32) exhibited higher rates, whereas the North (41.4%, 12/29) and Southwest (46.7%, 14/30) showed lower rates (Figure 2).

Furthermore, longitudinal analysis demonstrated a significant increase in the prevalence of cluster AZR (Cochran-Armitage trend test, *p* = 0.012). Cluster AZR replaced group AZR-ADJ and became the predominant resistant population (63.9%, 69/108, 95% CI 54.1%−72.9%) in China during 2013–2016; upon the most recent four years, cluster AZR has raised up to 62.3% (38/61, 95% CI 49.0%−74.4%) of FLU-resistant isolates, whereas group AZR-ADJ accounted for only 9.8% (6/61, 95% CI 3.7%−20.2%) (Figure 1(B)).

### Polyploidies, aneuploidies, large structural variations and LOH in C. tropicalis

Ploidy analysis demonstrated that most *C. tropicalis* isolates were diploid (98.0%, 592/604), whereas polyploidies were rare, including triploid (1.5%, 9/604), tetraploid (0.3%, 2/604), and octoploid (0.2%, 1/604) (Supplementary Table S1). Further analysis indicated that aneuploidy occurred in 15 isolates. Aneuploid events were most frequently found on chromosome 6 (n = 8), followed by chromosome 5 (n = 3) (Supplementary Table S1). Large structural variations were observed in 6.8% (41/604) of isolates, with three isolates harbouring variations on multiple chromosomes. Most large structural variations were found on chromosome 4 (n = 31), with the remaining on chromosomes 1, 2, 3, 5 or 6 (n = 11) (Supplementary Table S1).

To investigate *ERG11* CNVs independent of tandem repeat duplication, we analyzed aneuploidies and large structural variations on chromosome 5 that contained *ERG11* in two non-cluster AZR isolates. One isolate harboured large structural variation on chromosome 5, and carried four copies of *ERG11*, exhibiting cross-resistance to FLU and VOR (Supplementary Table S1). The other isolate exhibited an increased *ERG11* copy numbers (n = 3) associated with chromosome 5 aneuploidy; however, this isolate remained susceptible to both FLU and VOR (Supplementary Table S1).

LOH events were widely observed in 91.2% (551/604) of isolates, and 2.0% (12/604) of isolates exhibited genome-wide high heterozygosity (Supplementary Table S1). Most LOH were found on chromosome R (81.3%, 491/604), followed by chromosome 6 (40.2%, 243/604). We further investigated the association between LOH events and *ERG11* heterozygosity in cluster AZR. Among 111 isolates carrying *ERG11* mutations, 85.7% (6/7) of isolates carrying homozygous mutations exhibited LOH, whereas all heterozygous isolates (104/104) lacked detectable LOH events.

### Genomic analysis identified strain replacement and dual-clade mixed infection

**Figure 4.**
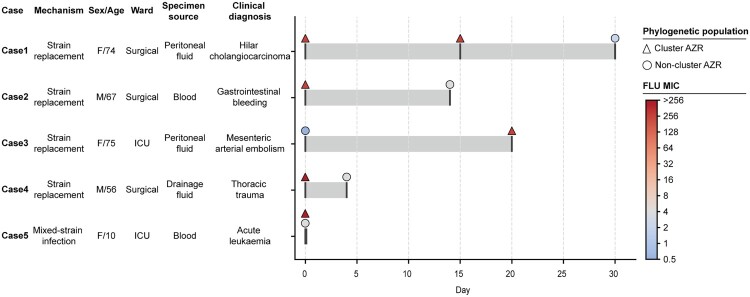
Phylogenetic analysis of five cases showing strain replacement or dual-clade mixed infection. Each timeline illustrates sequential isolates collected from individual patients, with symbols indicating phylogenetic population and colours representing fluconazole (FLU) minimum inhibitory concentrations (MICs).

Antifungal susceptibility testing identified five series of isolates from single patients that exhibited azole susceptibility shifts within a single infection episode (less than one month). The key clinical and laboratory examination information of these five cases were summarized in Figure 4. In case 1, *C. tropicalis* was persistently isolated from peritoneal fluid over a 30-day period. The patient had a diagnosis of Hilar cholangiocarcinoma. In case 2, *C. tropicalis* was persistently isolated from blood over a 14-day period. The patient was diagnosed with gastrointestinal bleeding. In case 3, *C. tropicalis* was persistently isolated from peritoneal fluid over a 20-day period. The patient had a diagnosis of mesenteric arterial embolism. In case 4, *C. tropicalis* was persistently isolated from drainage fluid within four days. The patient was diagnosed with thoracic trauma. In case 5, two *C. tropicalis* strains with different susceptibility phenotypes were simultaneously isolated from blood on the same day. The patient had a diagnosis of acute leukaemia.

WGS was performed to determine the genetic relatedness of strains isolated during a single infection episode and to identify the underlying mechanisms of azole resistance. For all five cases, MLST and phylogenetic analysis suggested that strains from the same patient did not cluster together, and exhibit high pairwise SNPs (ranging from 42577 to 78243). All FLU-resistant strains belonged to cluster AZR, carrying *ERG11* Y132F and S154F amino acid substitution, along with increased *ERG11* copy numbers (ranging from 5 to 8) (Figure 4). In cases 1–4, sequential isolates with different genetic backgrounds were recovered within a single infection episode, demonstrating strain replacement events (Figure 4). In case 5, strains were isolated from blood cultures on the same day, suggesting a dual-clade mixed infection (Figure 4).

### In vitro susceptibilities to other antifungal agents and resistance mechanisms

Over 99% of isolates remained susceptible to all three echinocandins tested (CAS, MF and AND) (Figure 1(A)). To further investigate the resistance mechanism, WGS was performed on three CAS-resistant isolates. All three isolates carried S654P mutation of the 1,3-β-glucan synthase gene *FKS1*, which was located within the hot spot 1 (HS1) region, and has been previously reported in *C. tropicalis* (Supplementary Table S1) [38]. For two isolates exhibiting multidrug resistance to FLU and CAS, phylogenetic analysis confirmed one isolate belonged to cluster AZR, and carried eight copies of *ERG11* along with Y132F and S154F mutations, whereas the other isolate harboured a V125A substitution in *ERG11* (Supplementary Table S1).

Over 99% of isolates were of WT phenotype to AMB (Figure 1(A)). The MIC_50_ and MIC_90_ values of AMB were 1 and 2 μg/mL, respectively (Figure 1(E)). As the absence of CBPs and ECVs, susceptibility to FC was analyzed using MIC_50_ and MIC_90_ values, which were 0.06 and 0.12 μg/mL, respectively (Figure 1(E)).

## Discussion

In recent years, the global burden of IFDs has increased substantially, causing approximately 6.5 million infections and 3.8 million deaths worldwide each year [39,40]. Among common fungal pathogens, *Candida* species are a predominant cause of fungal bloodstream infections, accounting for 85% of cases [41–43]. Among non-*albicans Candida* species, *C. tropicalis* represented a major threat, with 30-day mortality of bloodstream infections (32%−52%) exceeding that of overall candidemia (30%−40%) [1].

Among the limited classes of antifungal agents, azoles are among the most widely used agents for candidiasis [41]. Notably, azole resistance exceeding 15% was reported in several countries across Europe and the Asia-Pacific region [19,44–47]. However, in this study, our latest two-year surveillance data indicated that FLU resistance has exceeded 40% in China, representing the highest level reported worldwide. Cross-resistance to FLU and VOR was observed in 37.0% of isolates, and 28.5% exhibited NWT phenotype to IZ. The proportion of PZ-NWT isolates was even higher (82.3%); however, as previously reported, this observation is likely attributable to the stringent PZ ECV applied to *C. tropicalis* (as summarized in Supplementary Table S2) [48].

The emergence and expansion of certain phylogenetic populations could drive shifts in antimicrobial resistance, which has been recognized in bacterial and fungal pathogens such as *Mycobacterium tuberculosis*, *Salmonella typhi*, *Clostridium difficile*, *Candida albicans*, *Candida auris* and *Aspergillus fumigatus* [49–54]. In Asia-Pacific region, previous MLST-based studies have shown that the increase of azole resistance can be attributed to the shift of predominant resistant population from MLST clade 5 to clade 4 [10,14,15]. In recent years, WGS has enabled in-depth investigation of the resistant population structure and dynamics. In our previous study, we noticed the emergence and rapid expansion of a subpopulation of clade 4, named cluster AZR, which was associated with the dramatic increase of azole resistance in China [14]. In this study, cluster AZR remained the most prevalent resistant cluster (62.3%), while group AZR-ADJ accounting for approximately 10% of overall FLU-resistant strains in CHIF-NET20-23. Recent genomic studies have identified cluster AZR in China, Singapore, and Malaysia, suggesting regional dissemination of this clone [14,55]; however, the limited availability of global WGS data may lead to underestimation of its transmission. In all, the emergence and rapid dissemination of cluster AZR have become a potential global threat and warrants further molecular epidemiology surveillance.

Of note, our study revealed that azole resistance rate varied among different clinical departments, which was higher in isolates from medical departments (55.0%), and lower in isolates from ICUs (33.7%). According to an Active Surveillance of Fungal Bloodstream Infections programme carried out in 303 hospitals in China during 2020, the isolation rate of *C. tropicalis* reached >50% in haematology department [56]. Recent studies further revealed that *C. tropicalis* has emerged as a major pathogen in patients with haematological disease, exhibiting high azole resistance and mortality rate [57–59]. This may be associated with extensive use of azoles for antifungal prophylaxis and empiric treatment in this population [41]. These findings highlight the need for continuous observation of these high-risk patients, and appropriate use of azoles during therapy. In addition, geographical variation in azole resistance may be associated with differences in local antifungal usage patterns, patient risk profiles, climatic and environmental factors [5,60,61]. However, the underlying factors remained incompletely understood and warrant further investigations.

Longitudinal analysis revealed a long-term increase of azole resistance over 14 surveillance years. However, the epidemiological data presented in this study could not fully explain the underlying factors that drove the transient decline and subsequent rebound in azole resistance rates during 2020–2023. Previous studies suggested that expanding use of echinocandins for empiric therapy and prophylaxis may contribute to antifungal resistance among *Candida* species [62,63]; however, our previous nationwide surveillance did not identify a direct correlation between clinical azole consumption and the increasing prevalence of azole resistance in *C. tropicalis* [64]. Potential contribution of clinical antifungal consumption to azole resistance dynamics warrants further investigation. Aside from clinical azoles, exposure to agricultural azoles may also drive antifungal resistance [6]. A recent study indicated that tebuconazole, a triazole fungicide widely used in agriculture, could induce resistance to clinical azoles in *C. tropicalis* [65]. Moreover, clade 4 azole-resistant *C. tropicalis* isolates, including cluster AZR strains, have been frequently recovered from environmental niches, including fruits, soils, and irrigation water, and exhibit high genetic relatedness to clinical isolates [66,67]. In *A. fumigatus*, population genomics also confirmed that acquisition of drug-resistance in human infections could be driven by the use of agricultural fungicides [52]. Future research and antifungal stewardship should be framed within the “One Health” perspective.

Consistent with previous reports, extensive LOH appears to be a common genomic feature of *C. tropicalis*, occurring across diverse phylogenetic populations and being detected in more than 90% of isolates in the present study [68]. In addition, LOH events were found to affect the zygosity of the key resistance-associated *ERG11* mutation in cluster AZR isolates. LOH has been recognized as an important genetic mechanism contributing to the evolution and adaptation in fungi [69,70]. In *C. albicans*, phylogenetic lineage-associated LOH events have also been reported in azole-resistant populations [53].

Of note, antifungal susceptibility profiles may shift within individual patients during therapy. A WGS-based study by McTaggart et al. reported the *in vivo* emergence of azole resistance in *C. tropicalis*, driven by the acquisition of *ERG11* key mutations or development of aneuploidies [71]. In contrast, in our WGS analysis of five cases with multiple isolates exhibiting differing azole susceptibility profiles, no evidence of *in vivo* microevolution was observed; instead, all FLU-resistant isolates from these patients belonged to cluster AZR, whereas the remaining non-resistant isolates were assigned to non-AZR phylogenetic lineages. A previous MLST-based study from Brazil also showed that isolates recovered from the same patient exhibited different DSTs, in line with our findings [72]. These findings warranted evaluation of the limitations of routine clinical laboratory practices in detecting resistant variants within mixed infections, as current clinical workflows commonly rely on a single colony per isolate. This highlights the need for lineage-aware surveillance and repeated susceptibility testing during therapy to guide appropriate antifungal management.

Several limitations of the present study were acknowledged. As a retrospective laboratory-based surveillance study primarily focused on the epidemiology and antifungal susceptibility of yeast pathogens, detailed patient-level clinical data, such as host immune status, antifungal treatment history, and clinical outcomes, were not systematically collected. Besides, hospital- and population-level denominator data, including patient-days, antifungal consumption, infection control-related information and other population-based epidemiological metrics, were not available, limiting further evaluation of factors underlying azole resistance incidence and temporal shifts. Third, although the WGS dataset included both previously published and newly sequenced isolates, the isolates were selected using different frameworks and partially different purposes, which may introduce potential selection bias. Despite these limitations, the large sample size and broad geographic coverage of the CHIF-NET study support the robustness of the overall findings.

In conclusion, our study provided an updated overview of the epidemiology, antifungal susceptibility profiles and population genomic characteristics of *C. tropicalis* isolates causing IFDs in China. Azole resistance continues to worsen and is associated with the expansion of the emerging predominant azole-resistant cluster AZR. Broader molecular epidemiological surveillance is warranted to define regional and global trends in antifungal resistance and clonal dynamics of *C. tropicalis*, to support the development of more effective antifungal stewardship strategies and to curb antifungal resistance through the “One Health” approach.

## Supplementary Material

Supplementary data_260601_final.pdf

## Data Availability

New short-read sequencing results of *C. tropicalis* isolates generated in this study have been deposited at the NCBI Sequence Read Archive under BioProject ID PRJNA1452242. Additional previously published WGS and MLST data used in this study (Supplementary Table S1) were sourced from the NCBI SRA database (https://www.ncbi.nlm.nih.gov/sra) and the PubMLST database (https://pubmlst.org), respectively.
