# Supplementary material for: Population genomics reveals a long-term increase in azole resistance associated with the emerging cluster AZR among Candida tropicalis causing invasive infections
Source: Emerg Microbes Infect. 2026 Jun 25;15(1):2695535. doi: 10.1080/22221751.2026.2695535 (PMC13348128; doi:10.1080/22221751.2026.2695535)
Supplement: Supplementary data_260601_final.pdf [file TEMI_A_2695535_SM0575.pdf]

Supplementary Table S1. Summary of the 604 *Candida tropicalis* isolates used for population genomic analysis in this study.

Position in tree	Strain ID no.	Other strain no.	NCBI SRA accession no.	Year	Sex	Age	Clinical services	Blood source	Geographical region	DST	MLST CC	MLST Clade	WGS cluster	Polyploidy	Aneuploidy	Large structure variation	LOH (chromosome)	Erg11p key substitution	*ERG11* Heterozygosity	*ERG11* CNV	Antifungal susceptibilities (MIC [mg/L]/Interpretation)									Sequenced in this study
																					FLU	VOR	IZ	PZ	CAS	MF	AND	AMB	FC	
1	F4024	14SF023	SRR23915593	2014	F	20	Medical	Yes	North	437	CCN3	Non-clade 4	Other	Diploid	-	-	2,R	-	-	2	2/S	0.12/S	0.25/WT	0.5/NWT	0.03/S	0.03/S	0.03/S	1/WT	0.06	
2	F3362	15XY256	SRR23915265	2015	M	54	Other wards	Yes	Central	10015	CCN89	Non-clade 4	Other	Diploid	-	-	-	-	-	2	2/S	0.25/I	0.5/WT	0.5/NWT	0.06/S	0.03/S	0.06/S	1/WT	0.06	
3	F3332	15HB046	SRR23915313	2015	M	27	Medical	Yes	North	437	CCN3	Non-clade 4	Other	Diploid	-	-	2,R	-	-	2	1/S	0.03/S	0.06/WT	0.12/WT	0.03/S	0.015/S	0.03/S	1/WT	0.06	
4	F3446	15XY257	SRR23915342	2015	M	54	Other wards	Yes	Central	10029	CCN3	Non-clade 4	Other	Diploid	-	-	1,2,4,R	-	-	2	2/S	0.5/I	0.5/WT	0.5/NWT	0.03/S	0.03/S	0.03/S	1/WT	0.06	
5	F3232	15NX137	SRR23915646	2015	F	25	ICU	Yes	Northwest	10009	CCN3	Non-clade 4	Other	Diploid	-	-	1,2,4,R	-	-	2	0.25/S	0.008/S	0.015/WT	0.015/WT	0.03/S	0.015/S	0.015/S	0.5/WT	0.06	
6	F9473	10BD107	SRR23915352	2010	M	64	Medical	Yes	North	10104	CCN90	Non-clade 4	Other	Diploid	-	-	2,3,5,6,R	-	-	2	2/S	0.12/S	0.25/WT	0.12/WT	0.12/S	0.03/S	0.12/S	0.5/WT	≤0.06	
7	2575	21PU1732	SRR38060866	2021	F	50	Outpatient/Emergency	Yes	North	394	CCN1	Non-clade 4	Other	Diploid	-	-	1,3,6,R	-	-	2	2/S	0.25/I	0.25/WT	0.25/NWT	0.12/S	0.06/S	0.25/S	1/WT	≤0.06	Yes
8	Z3724	21XH724	SRR38060865	2021	F	51	Surgical	No	Central	394	CCN1	Non-clade 4	Other	Diploid	-	-	1,3,6,R	-	-	2	2/S	0.12/S	0.12/WT	0.12/WT	0.06/S	0.06/S	0.12/S	1/WT	≤0.06	Yes
9	Z3717	21XH717	SRR38060717	2021	F	51	Surgical	No	Central	394	CCN1	Non-clade 4	Other	Diploid	-	-	1,3,6,R	-	-	2	2/S	0.12/S	0.12/WT	0.12/WT	0.06/S	0.06/S	0.12/S	0.5/WT	≤0.06	Yes
10	F3588	13J3049	SRR23915520	2013	N/A	N/A	Surgical	Yes	East	10042	CCN1	Non-clade 4	Other	Triploid	chr 6-0.5×	-	2,3,R	-	-	2	2/S	0.12/S	0.25/WT	0.25/WT	0.03/S	0.015/S	0.015/S	0.5/WT	0.06	
11	F3630	13J3048	SRR23915644	2013	N/A	N/A	Medical	No	East	10042	CCN1	Non-clade 4	Other	Triploid	chr 6-0.5×	-	2,3,R	-	-	2	2/S	0.12/S	0.12/WT	0.12/WT	0.03/S	0.015/S	0.015/S	0.5/WT	0.06	
12	F3656	14JD036	SRR23915630	2014	M	41	Surgical	No	Northeast	1396	CCN1	Non-clade 4	Other	Diploid	-	-	2,R	-	-	2	256/R	8/R	16/NWT	8/NWT	0.03/S	0.03/S	0.03/S	1/WT	0.06	
13	F3792	14J3055	SRR23915306	2014	N/A	N/A	Medical	Yes	East	443	CCN1	Non-clade 4	Other	Diploid	-	-	1,3,R	-	-	2	2/S	0.06/S	0.12/WT	0.12/WT	0.03/S	0.03/S	0.015/S	0.5/WT	0.5	
14	F3716	14G1216	SRR23915507	2014	M	67	Medical	No	South	443	CCN1	Non-clade 4	Other	Diploid	-	-	2,3,R	-	-	2	2/S	0.12/S	0.25/WT	0.25/WT	0.06/S	0.03/S	0.12/S	1/WT	0.06	
15	F4042	15CZ006	SRR23915583	2015	M	61	Surgical	Yes	East	443	CCN1	Non-clade 4	Other	Diploid	-	-	2,3,4,R	-	-	2	2/S	0.25/I	0.5/WT	0.5/NWT	0.03/S	0.03/S	0.03/S	1/WT	0.06	
16	F3682	16HZ092	SRR23915413	2016	F	54	Surgical	Yes	Central	394	CCN1	Non-clade 4	Other	Diploid	-	-	3,R	-	-	2	2/S	0.12/S	0.25/WT	0.12/WT	0.25/S	0.06/S	0.12/S	0.5/WT	0.06	
17	F4094	15HX892	SRR23915578	2015	F	50	Medical	Yes	Southwest	394	CCN1	Non-clade 4	Other	Diploid	-	-	3,4,5,6,R	-	-	2	256/R	8/R	0.5/WT	0.5/NWT	0.25/S	0.06/S	0.12/S	0.5/WT	0.06	
18	F3350	15H1472	SRR23915296	2015	F	55	ICU	Yes	Northeast	434	CCN1	Non-clade 4	Other	Diploid	-	-	2,3,6,R	-	-	2	4/SDD	0.5/I	0.5/WT	0.5/NWT	0.03/S	0.03/S	0.03/S	1/WT	0.06	
19	F3462	15H1466	SRR23915335	2015	F	55	Surgical	Yes	Northeast	434	CCN1	Non-clade 4	Other	Diploid	-	-	2,3,6,R	-	-	2	8/R	0.5/I	0.5/WT	1/NWT	0.03/S	0.03/S	0.03/S	1/WT	0.06	
20	F3990	15JS486	SRR23915280	2015	F	69	Surgical	No	East	434	CCN1	Non-clade 4	Other	Diploid	-	-	3,6,R	-	-	2	0.25/S	0.008/S	0.12/WT	0.03/WT	0.03/S	0.015/S	0.06/S	0.5/WT	0.06	
21	F3594	16GY025	SRR23915517	2016	M	58	Medical	Yes	Southwest	10044	CCN1	Non-clade 4	Other	Diploid	-	-	1,3,6,R	-	-	2	2/S	0.12/S	0.25/WT	0.25/WT	0.06/S	0.03/S	0.12/S	1/WT	0.06	
22	F4138	11XY017	SRR23915372	2011	M	26	ICU	Yes	Central	394	CCN1	Non-clade 4	Other	Diploid	-	-	3,6,R	-	-	2	2/S	0.12/S	0.25/WT	0.25/WT	0.03/S	0.03/S	0.015/S	0.5/WT	0.06	
23	F3718	15GX104	SRR23915505	2015	M	3	Other wards	Yes	South	981	CCN1	Non-clade 4	Other	Diploid	-	-	2,4,6,R	-	-	2	2/S	0.12/S	0.25/WT	0.25/WT	0.03/S	0.03/S	0.12/S	0.5/WT	0.12	
24	F3686	16HX940	SRR23915411	2016	F	52	ICU	Yes	Southwest	331	CCN1	Non-clade 4	Other	Diploid	-	-	1,5,R	-	-	2	2/S	0.25/I	0.25/WT	0.25/WT	0.25/S	0.06/S	0.12/S	0.5/WT	0.06	
25	F3986	14AH064	SRR23915282	2014	M	48	Medical	Yes	East	331	CCN1	Non-clade 4	Other	Diploid	-	-	1,5,R	-	-	2	2/S	0.12/S	0.12/WT	0.12/WT	0.015/S	0.03/S	0.015/S	1/WT	0.06	
26	F3170	15H1504	SRR23915537	2015	F	38	ICU	Yes	Northeast	394	CCN1	Non-clade 4	Other	Triploid	-	-	1,2,3,4,6,R	-	-	2	1/S	0.03/S	0.12/WT	0.12/WT	0.06/S	0.015/S	0.015/S	0.5/WT	0.06	
27	F3710	13X1016	SRR23915399	2013	F	16	Medical	Yes	Northwest	331	CCN1	Non-clade 4	Other	Diploid	-	-	6,R	-	-	2	2/S	0.25/I	0.25/WT	0.25/WT	0.03/S	0.03/S	0.015/S	0.5/WT	0.06	
28	F3410	15GH455	SRR23915541	2015	F	70	Surgical	No	North	10022	CCN1	Non-clade 4	Other	Diploid	-	-	1,4,6,R	-	-	2	0.5/S	0.03/S	0.03/WT	0.03/WT	0.03/S	0.03/S	0.015/S	0.5/WT	0.06	
29	F3886	11XY001	SRR23915377	2011	F	54	Surgical	Yes	Central	331	CCN1	Non-clade 4	Other	Diploid	-	-	1,2,3,4,6,R	-	-	2	2/S	0.12/S	0.25/WT	0.25/WT	0.03/S	0.03/S	0.015/S	0.5/WT	0.06	
30	F3450	14Z2111	SRR23915341	2014	M	53	ICU	Yes	East	331	CCN1	Non-clade 4	Other	Diploid	-	-	6,R	-	-	2	1/S	0.06/S	0.12/WT	0.12/WT	0.06/S	0.015/S	0.06/S	0.5/WT	0.06	
31	F4010	15CQ246	SRR23915598	2015	M	62	Surgical	No	Southwest	394	CCN1	Non-clade 4	Other	Diploid	-	-	1,2,3,4,6,R	-	-	2	1/S	0.06/S	0.12/WT	0.12/WT	0.12/S	0.03/S	0.015/S	0.5/WT	0.06	
32	F4048	15DP065	SRR23915277	2015	M	46	ICU	Yes	Southwest	394	CCN1	Non-clade 4	Other	Diploid	-	-	1,2,3,4,6,R	-	-	2	256/R	8/R	0.5/WT	1/NWT	0.06/S	0.06/S	0.12/S	0.5/WT	0.06	
33	F3694	14H1407	SRR23915408	2014	M	53	ICU	No	Northeast	394	CCN1	Non-clade 4	Other	Diploid	-	chr 4-pos 1-3×	3,6,R	-	-	2	1/S	0.12/S	0.12/WT	0.12/WT	0.03/S	0.03/S	0.03/S	1/WT	0.06	
34	F3988	15JS480	SRR23915281	2015	F	59	Surgical	No	East	394	CCN1	Non-clade 4	Other	Diploid	-	-	3,6,R	-	-	2	2/S	0.12/S	0.25/WT	0.12/WT	0.12/S	0.06/S	0.12/S	0.5/WT	0.06	
35	F9514	11HX250	SRR23915236	2011	M	64	Surgical	No	Southwest	394	CCN1	Non-clade 4	Other	Diploid	-	-	3,6,R	-	-	2	2/S	0.06/S	0.12/WT	0.12/WT	0.06/S	0.015/S	0.06/S	1/WT	0.12	
36	F4036	15A1077	SRR23915586	2015	M	70	Surgical	No	Northwest	394	CCN1	Non-clade 4	Other	Diploid	-	chr 4-pos 1-2.5×	1,2,3,6,R	-	-	2	2/S	0.25/I	0.25/WT	0.25/WT	0.03/S	0.03/S	0.03/S	0.5/WT	0.06	
37	F3736	15G1204	SRR23915496	2015	F	59	Medical	Yes	South	394	CCN1	Non-clade 4	Other	Diploid	chr 6-1.5×	-	3,6,R	-	-	2	4/SDD	0.25/I	0.25/WT	0.25/WT	0.03/S	0.015/S	0.12/S	0.5/WT	0.06	
38	F9483	13BD298	SRR23915452	2013	M	83	ICU	No	North	10106	CCN1	Non-clade 4	Other	Diploid	-	-	3,6,R	-	-	2	2/S	0.25/I	0.25/WT	0.25/WT	0.03/S	0.03/S	0.12/S	1/WT	≤0.06	
39	F3572	15PU989	SRR23915527	2015	M	64	Surgical	Yes	North	394	CCN1	Non-clade 4	Other	Diploid	-	-	3,6,R	-	-	2	2/S	0.25/I	0.25/WT	0.25/WT	0.03/S	0.06/S	0.12/S	1/WT	0.06	
40	F3226	16K1100	SRR23915513	2016	F	65	Surgical	No	Southwest	10008	CCN1	Non-clade 4	Other	Diploid	-	-	3,6,R	-	-	2	256/R	8/R	16/NWT	8/NWT	0.25/S	0.06/S	0.25/S	1/WT	0.25	
41	F3408	15GH457	SRR23915542	2015	F	58	Surgical	No	North	394	CCN1	Non-clade 4	Other	Diploid	-	-	2,3,6,R	-	-	2	1/S	0.03/S	0.06/WT	0.06/WT	0.03/S	0.03/S	0.015/S	0.5/WT	0.06	
42	F4014	15J2048	SRR23915597	2015	M	61	ICU	No	East	394	CCN1	Non-clade 4	Other	Diploid	-	-	3,6,R	-	-	2	2/S	0.25/I	0.25/WT	0.25/WT	0.06/S	0.03/S	0.12/S	0.25/WT	0.06	
43	F3476	15TJ689	SRR23915328	2015	M	85	Surgical	No	Central	331	CCN1	Non-clade 4	Other	Diploid	-	-	6,R	-	-	2	4/SDD	0.5/I	0.5/WT	0.5/NWT	0.12/S	0.06/S	0.12/S	1/WT	0.06	
44	F9503	09HX011	SRR23915249	2009	M	62	Medical	Yes	Southwest	331	CCN1	Non-clade 4	Other	Diploid	-	-	1,2,4,R	-	-	2	2/S	0.12/S	0.25/WT	0.12/WT	0.03/S	0.03/S	0.03/S	1/WT	≤0.06	
45	F3738	16F1125	SRR23915493	2016	M	75	Surgical	Yes	East	394	CCN1	Non-clade 4	Other	Diploid	-	-	6,R	-	-	2	2/S	0.25/I	0.25/WT	0.5/NWT	0.06/S	0.06/S	0.12/S	1/WT	0.06	
46	F3590	13RJ149	SRR23915519	2013	M	63	ICU	Yes	East	10043	CCN91	Non-clade 4	Other	Diploid	-	chr 4-pos 1-2×	1,4,R	-	-	2	2/S	0.25/I	0.25/WT	0.25/WT	0.06/S	0.03/S	0.06/S	1/WT	0.06	
47	F4002	13RJ151	SRR23915600	2013	M	56	ICU	Yes	East	10043	CCN91	Non-clade 4	Other	Diploid	-	chr 4-pos 1-4×	1,4,R	-	-	2	1/S	0.12/S	0.25/WT	0.25/WT	0.03/S	0.03/S	0.03/S	1/WT	0.06	
48	F3908	15CQ217	SRR23915481	2015	M	64	ICU	No	Southwest	331	CCN1	Non-clade 4	Other	Diploid	-	-	2,4,R	-	-	2	2/S	0.12/S	0.25/WT	0.12/WT	0.12/S	0.06/S	0.12/S	0.5/WT	0.06	
49	F4080	15H1420	SRR23915259	2015	M	62	Surgical	Yes	Northeast	331	CCN1	Non-clade 4	Other	Diploid	-	-	2,4,R	-	-	2	1/S	0.03/S	0.06/WT	0.06/WT	0.03/S	0.03/S	0.03/S	0.5/WT	0.06	
50	F3190	15H4224	SRR23915432	2015	F	61	ICU	Yes	Northeast	331	CCN1	Non-clade 4	Other	Diploid	-	-	2,3,6,R	-	-	2	0.5/S	0.008/S	0.06/WT	0.03/WT	0.03/S	0.015/S	0.015/S	0.5/WT	0.06	
51	5011	21GZ1443	SRR38060778	2021	F	67	Surgical	No	South	12024	CCN1	Non-clade 4	Other	Diploid	-	-	3,4,6,R	-	-	2	1/S	0.06/S	0.06/WT	0.06/WT	0.06/S	0.03/S	0.03/S	0.5/WT	≤0.06	Yes
52	4824	21GZ1256	SRR38060767	2021	F	15	Outpatient/Emergency	Yes	South	434	CCN1	Non-clade 4	Other	Diploid	-	-	1,2,3,6,R	-	-	2	1/S	0.03/S	0.06/WT	0.03/WT	0.06/S	0.03/S	0.06/S	0.25/WT	≤0.06	Yes
53	5025	21GZ1457	SRR38060804	2021	F	54	ICU	Yes	South	394	CCN1	Non-clade 4	Other	Diploid	-	-	2,3,5,6,R	-	-	2	2/S	0.12/S	0.06/WT	0.12/WT	0.03/S	0.03/S	0.12/S	0.5/WT	≤0.06	Yes
54	5024	21GZ1456	SRR38060793	2021	M	69	ICU	Yes	South	394	CCN1	Non-clade 4	Other	Diploid	-	-	2,5,R	-	-	2	1/S	0.03/S	0.06/WT	0.03/WT	0.06/S	0.03/S	0.12/S	0.5/WT	≤0.06	Yes
55	3011	21SJ681	SRR38060830	2021	F	68	Outpatient/Emergency	Yes	Northeast	394	CCN1	Non-clade 4	Other	Diploid	-	-	1,2,4,R	R388G	Heterozygous	2	2/S	0.12/S	0.12/WT	0.12/WT	0.06/S	0.06/S	0.12/S	1/WT	≤0.06	Yes
56	2673	21PU1830	SRR38060819	2021	F	93	ICU	No	North	12029	CCN1	Non-clade 4	Other	Diploid	-	-	2,3,4,R	-	-	2	1/S	0.06/S	0.12/WT	0.12/WT	0.06/S	0.06/S	0.03/S	1/WT	≤0.06	Yes
57	Z2269	20NX530	SRR38060808	2020	F	41	Surgical	Yes	Northwest	910	CCN41	Non-clade 4	Other	Diploid	-	-	R	-	-	2	2/S	0.12/S	0.25/WT	0.25/NWT	0.03/S	0.03/S	0.06/S	1/WT	≤0.06	Yes
58	Z2223	20NX484	SRR38060864	2020	F	41	ICU	Yes	Northwest	910	CCN41	Non-clade 4	Other	Diploid	-	-	R	-	-	2	1/S	0.03/S	0.12/WT	0.06/WT	0.06/S	0.03/S	0.12/S	0.5/WT	≤0.06	Yes
59	F3852	15FJ451	SRR23915395	2015	F	25	Medical	Yes	East	10072	CCN87	Non-clade 4	Other	Diploid	-	-	3,R	-	-	2	2/S	0.12/S	0.5/WT	0.5/NWT	0.06/S	0.06/S	0.12/S	0.5/WT	0.12	
60	F3868	15HX873	SRR23915386	2015	M	63	ICU	No	Southwest	1335	CCN88	Non-clade 4	Other	Diploid	-	-	1,3,4,6,R	-	-	2	256/R	8/R	0.5/WT	0.5/NWT	0.25/S	0.06/S	0.12/S	0.5/WT	0.06	
61	F3482	14HX757	SRR23915436	2014	F	63	Medical	Yes	Southwest	1335	CCN88	Non-clade 4	Other	Diploid	-	-	1,3,6,R	-	-	2	0.5/S	0.03/S	0.03/WT	0.03/WT	0.03/S	0.03/S	0.015/S	0.5/WT	0.06	
62	F9517	12HX423	SRR23915233	2012	M	35	ICU	Yes	Southwest	359	CCN23	Non-clade 4	Other	Diploid	-	-	2,3,4,5,R	-	-	2	1/S	0.06/S	0.12/WT	0.12/WT	0.03/S	0.03/S	0.03/S	0.5/WT	≤0.06	
63	F3432	15GF052	SRR23915348	2015	F	83	Surgical	Yes	South	811	CCN23	Non-clade 4	Other	Diploid	-	-	1,4,5,6,R	-	-	2	0.5/S	0.03/S	0.06/WT	0.03/WT	0.03/S	0.015/S	0.015/S	0.5/WT	0.06	
64	4875	21GZ1307	SRR38060853	2021	F	47	Surgical	Yes	South	525	CCN11	Non-clade 4	Other	Diploid	-	-	3,6,R	-	-	2	2/S	0.06/S	0.12/WT	0.06/WT	0.06/S	0.06/S	0.12/S	0.25/WT	≤0.06	Yes
65	Z2115	21LR674	SRR38060842	2021	M	78	ICU	No	Northeast	525	CCN11	Non-clade 4	Other	Diploid	-	-	3,6,R	-	-	2	2/S	0.06/S	0.12/WT	0.12/WT	0.03/S	0.03/S	≤0.015/S	0.5/WT	≤0.06	Yes
66	Z2113	21LR672	SRR38060750	2021	M	78	ICU	No	Northeast	525	CCN11	Non-clade 4	Other	Diploid	-	-	3,6,R	-	-	2	2/S	0.06/S	0.12/WT	0.12/WT	0.03/S	0.03/S	≤0.015/S	1/WT	≤0.06	Yes
67	3019	21SJ689	SRR38060739	2021	M	59	Medical	Yes	Northeast	525	CCN11	Non-clade 4	Other	Diploid	-	-	1,R	-	-	2	2/S	0.06/S	0.25/WT	0.25/NWT	0.06/S	0.06/S	0.12/S	1/WT	≤0.06	Yes
68	Z2600	21PU1757	SRR38060728	2021	F	67	Surgical	No	North	525	CCN11	Non-clade 4	Other	Diploid	-	-	1,4,5,R	-	-	2	2/S	0.12/S	0.12/WT	0.12/WT	0.06/S	0.06/S	0.25/S	0.5/WT	≤0.06	Yes
69	Z2457	20PU1614	SRR38060721	2020	F	66	ICU	No	North	525	CCN11	Non-clade 4	Other	Diploid	-	-	1,2,4,5,R	-	-	2	1/S	0.06/S	0.12/WT	0.12/WT	0.03/S	0.06/S	0.12/S	0.5/WT	≤0.06	Yes
70	F3720	15GX080	SRR23915504	2015	F	20	Medical	Yes	South	10056	CCN7	Non-clade 4	Other	Diploid	-	-	1,2,4,5,R	-	-	2	1/S	0.12/S	0.12/WT	0.12/WT	0.03/S	0.03/S	0.12/S	0.5/WT	0.06	
71	F3962	15JS515	SRR23915295	2015	M	46	Surgical	No	East	526	CCN11	Non-clade 4	Other	Diploid	-	-	R	-	-	2	0.5/S	0.03/S	0.06/WT	0.03/WT	0.06/S	0.03/S	0.12/S	0.25/WT	0.12	
72	F3960	15JS512	SRR23915298	2015	F	68	Medical	Yes	East	526	CCN11	Non-clade 4	Other	Diploid	-	-	R	-	-	2	64/R	2/R	1/NWT	2/NWT	0.25/S	0.03/S	0.06/S	2/WT	0.06	
73	F3456	14ZR076	SRR23915338	2014	M	73	Medical	Yes	Northeast	525	CCN11	Non-clade 4	Other	Diploid	-	-	3,6,R	-	-	2	1/S	0.06/S	0.12/WT	0.12/WT	0.015/S	0.03/S	0.03/S	1/WT	0.06	
74	F3768	14AH075	SRR23915318	2014	F	38	Medical	Yes	East	525	CCN11	Non-clade 4	Other	Diploid	-	-	R	-	-	2	4/SDD	0.25/I	0.5/WT	0.5/NWT	0.03/S	0.03/S	0.03/S	1/WT	0.06	
75	F4062	15GH524	SRR23915269	2015	M	68	Surgical	No	North	525	CCN11	Non-clade 4	Other	Diploid	-	-	4,6,R	-	-	2	1/S	0.12/S	0.25/WT	0.25/WT	0.03/S	0.03/S	0.12/S	0.5/WT	0.06	
76	F9487	10H1053	SRR23915448	2010	F	56	ICU	No	Northeast	525	CCN11	Non-clade 4	Other	Diploid	-	-	3,6,R	-	-	2	2/S	0.12/S	0.25/WT	0.25/WT	0.03/S	0.015/S	0.015/S	1/WT	≤0.06	
77	F3966	15LZ101	SRR23915293	2015	F	50	Surgical	No	Northwest	525	CCN11	Non-clade 4	Other	Diploid	-	-	1,3,5,R	-	-	2	1/S	0.12/S	0.12/WT	0.12/WT	0.03/S	0.03/S	0.03/S	1/WT	0.06	
78	F3744	11XY002	SRR23915490	2011	M	61	Medical	Yes	Central	525	CCN11	Non-clade 4	Other	Diploid	-	chr 4-pos 1-2×	4,5,6,R	-	-	2	4/SDD	0.25/I	0.5/WT	0.5/NWT	0.06/S	0.03/S	0.015/S	1/WT	0.06	
79	F3708	13X1007	SRR23915400	2013	M	72	ICU	No	Northwest	525	CCN11	Non-clade 4	Other	Diploid	-	-	1,4,6,R	-	-	2	8/R	0.5/I	0.5/WT	0.5/NWT	0.03/S	0.03/S	0.015/S	1/WT	0.06	
80	F3740	15ZD335	SRR23915492	2015	F	84	Outpatient/Emergency	Yes	East	525	CCN11	Non-clade 4	Other	Diploid	-	-	1,3,4,6,R	-	-	2	2/S	0.25/I	0.25/WT	0.25/WT	0.03/S	0.03/S	0.12/S	0.5/WT	0.06	
81	F3488	14G1187	SRR23915433	2014	M	26	ICU	Yes	South	525	CCN11	Non-clade 4	Other	Diploid	-	-	1,3,4,6,R	-	-	2	128/R	2/R	16/NWT	8/NWT	0.03/S	0.03/S	0.12/S	0.5/WT	0.06	
82	F4034	15LZ102	SRR23915587	2015	F	44	Medical	Yes	Northwest	525	CCN11	Non-clade 4	Other	Diploid	-	-	4,6,R	-	-	2	2/S	0.06/S	0.12/WT	0.25/WT	0.03/S	0.03/S	0.015/S	1/WT	0.06	
83	F3670	16GY016	SRR23915420	2016	M	63	ICU	Yes	Southwest	525	CCN11	Non-clade 4	Other	Diploid	-	-	4,6,R	-	-	2	8/R	0.5/I	0.5/WT	1/NWT	0.03/S	0.06/S	0.06/S	1/WT	0.06	
84	F4108	14PU811	SRR23915571	2014	M	71	Surgical	No	North	525	CCN11	Non-clade 4	Other	Diploid	-	-	6,R	-	-	2	4/SDD	0.5/I	16/NWT	8/NWT	0.03/S	0.03/S	0.12/S	1/WT	0.06	
85	F3848	15FJ455	SRR23915397	2015	F	48	ICU	Yes	East	525	CCN11	Non-clade 4	Other	Diploid	-	-	4,6,R	-	-	2	4/SDD	0.25/I	0.5/WT	0.5/NWT	0.06/S	0.06/S	0.12/S	1/WT	0.06	
86	F3380	15FJ462	SRR23915248	2015	F	13	Outpatient/Emergency	Yes	East	525	CCN11	Non-clade 4	Other	Diploid	-	-	4,6,R	-	-	2	4/SDD	0.25/I	0.5/WT	0.5/NWT	0.06/S	0.03/S	0.12/S	0.5/WT	0.06	
87	F3796	14DL026	SRR23915304	2014	M	71	Medical	Yes	Northeast	525	CCN11	Non-clade 4	Other	Diploid	-	-	4,6,R	-	-	2	256/R	4/R	16/NWT	2/NWT	0.25/S	0.03/S	0.12/S	1/WT	0.06	
88	F3856	16F1112	SRR23915393	2016	F	42	ICU	Yes	East	525	CCN11	Non-clade 4	Other	Diploid	-	-	2,6,R	-	-	2	16/R	1/R	0.25/WT	0.25/WT	0.06/S	0.06/S	0.12/S	1/WT	0.06	
89	F9476	12BD206	SRR23915460	2012	M	87	ICU	No	North	525	CCN11	Non-clade 4	Other	Diploid	-	-	2,3,5,6,R	G464S	Homozygous	2	>256/R	4/R	1/NWT	1/NWT	0.06/S	0.03/S	0.03/S	0.5/WT	≤0.06	
90	F3196	15A1037	SRR23915666	2015	F	85	ICU	Yes	Northwest	525	CCN11	Non-clade 4	Other	Diploid	-	-	1,2,3,5,6,R	-	-	2	0.5/S	0.008/S	0.06/WT	0.03/WT	0.06/S	0.015/S	0.06/S	0.25/WT	0.06	
91	3712	21XH712	SRR38060720	2021	F	64	ICU	No	Central	525	CCN11	Non-clade 4	Other	Diploid	-	-	6,R	-	-	2	2/S	0.12/S	0.12/WT	0.25/NWT	0.06/S	0.06/S	0.25/S	1/WT	≤0.06	Yes
92	3589	21XH589	SRR38060719	2021	F	59	ICU	Yes	Central	525	CCN11	Non-clade 4	Other	Diploid	-	-	1,4,6,R	-	-	2	2/S	0.12/S	0.25/WT	0.25/NWT	0.06/S	0.06/S	0.03/S	0.5/WT	≤0.06	Yes
93	L1246	23PU2274	SRR38060718	2023	M	27	Outpatient/Emergency	No	North	525	CCN11	Non-clade 4	Other	Diploid	-	-	6,R	-	-	2	128/R	8/R	1/NWT	2/NWT	0.03/S	0.03/S	0.25/S	0.5/WT	≤0.06	Yes
94	L1285	23PU2313	SRR38060716	2023	M	27	Outpatient/Emergency	No	North	525	CCN11	Non-clade 4	Other	Diploid	-	-	6,R	-	-	2	>256/R	>8/R	>16/NWT	>8/NWT	0.015/S	0.03/S	0.12/S	0.5/WT	≤0.06	Yes
95	L1253	23PU2281	SRR38060715	2023	M	27	Outpatient/Emergency	No	North	525	CCN11	Non-clade 4	Other	Diploid	-	-	2,6,R	-	-	2	256/R	8/R	2/NWT	2/NWT	0.03/S	0.03/S	0.25/S	0.5/WT	≤0.06	Yes
96	L1239	23PU2267	SRR38060714	2023	M	15	ICU	Yes	North	978	CCN11	Non-clade 4	Other	Diploid	-	-	4,5,6,R	-	-	2	>256/R	>8/R	>16/NWT	>8/NWT	0.03/S	0.03/S	≤0.015/S	1/WT	≤0.06	Yes
97	L1237	23PU2265	SRR38060713	2023	M	15	Other wards	Yes	North	978	CCN11	Non-clade 4	Other	Diploid	-	-	4,5,6,R	-	-	2	>256/R	>8/R	>16/NWT	>8/NWT	0.015/S	0.03/S	≤0.015/S	1/WT	≤0.06	Yes
98	L1381	23PU2409	SRR38060712	2023	M	15	Medical	No	North	978	CCN11	Non-clade 4	Other	Diploid	-	chr 5-pos 1-2×	4,5,6,R	-	-	4	8/R	4/R	>16/NWT	>8/NWT	0.03/S	0.03/S	≤0.015/S	0.5/WT	≤0.06	Yes
99	L1371	23PU2399	SRR38060711	2023	M	15	Medical	Yes	North	978	CCN11	Non-clade 4	Other	Diploid	-	-	4,5,6,R	-	-	2	>256/R	>8/R	>16/NWT	>8/NWT	0.015/S	0.03/S	≤0.015/S	1/WT	≤0.06	Yes
100	L1231	23PU2259	SRR38060782	2023	M	15	Other wards	No	North	978	CCN11	Non-clade 4	Other	Diploid	-	-	4,5,6,R	-	-	2	>256/R	>8/R	>16/NWT	>8/NWT	0.03/S	0.03/S	≤0.015/S	1/WT	≤0.06	Yes
101	Z6121	21HS1305	SRR38060781	2021	M	76	Medical	No	East	12030	CCN7	Non-clade 4	Other	Diploid	chr 5-1.5×	pos 1-1.5×, chr 2-pos	1,2,3,4,5,6,R	-	-	3	1/S	0.03/S	0.12/WT	0.12/WT	0.06/S	0.015/S	≤0.015/S	0.5/WT	≤0.06	Yes
102	4941	21GZ1373	SRR38060780	2021	M	78	ICU	Yes	South	1436	CCN7	Non-clade 4	Other	Diploid	-	-	1,4,5,R	-	-	2	1/S	0.06/S	0.06/WT	0.06/WT	0.06/S	0.06/S	0.12/S	1/WT	≤0.06	Yes
103	Z6114	21HS1298	SRR38060779	2021	M	76	Medical	No	East	1436	CCN7	Non-clade 4	Other	Diploid	chr 6-0.5×	-	1,2,3,4,5,6,R	-	-	2	2/S	0.12/S	0.06/WT	0.03/WT	0.12/S	0.015/S	≤0.015/S	0.5/WT	≤0.06	Yes
104	L3677	23XH827	SRR38060777	2023	M	22	ICU	Yes	Central	1346	CCN7	Non-clade 4	Other	Diploid	-	-	2,4,5,R	-	-	2	8/R	1/R	0.25/WT	0.5/NWT	0.06/S	0.03/S	0.12/S	1/WT	≤0.06	Yes
105	L3676	23XH826	SRR38060776	2023	M	22	Outpatient/Emergency	Yes	Central	1346	CCN7	Non-clade 4	Other	Diploid	-	-	2,4,5,R	-	-	2	32/R	0.5/I	0.25/WT	0.25/NWT	0.03/S	0.03/S	0.25/S	1/WT	≤0.06	Yes
106	2670	21PU1827	SRR38060775	2021	M	32	Medical	No	North	343	CCN45	Non-clade 4	Other	Diploid	-	-	1,3,4	-	-	2	1/S	0.06/S	0.12/WT	0.12/WT	0.03/S	0.06/S	0.12/S	0.5/WT	≤0.06	Yes
107	F3474	15TJ690	SRR23915329	2015	M	56	ICU	Yes	Central	489	CCN49	Non-clade 4	Other	Diploid	-	-	1,2,5,6,R	-	-	2	1/S	0.03/S	0.12/WT	0.06/WT	0.06/S	0.06/S	0.12/S	1/WT	0.06	
108	F3478	14ZD330	SRR23915327	2014	M	80	ICU	Yes	East	965	CCN115	Non-clade 4	Other	Diploid	-	-	2,6,R	-	-	2	1/S	0.12/S	0.25/WT	0.25/WT	0.12/S	0.03/S	0.03/S	1/WT	0.06	
109	F3746	16XY288	SRR23915489	2016	M	72	Surgical	No	Central	965	CCN115	Non-clade 4	Other	Diploid	-	chr 4-pos 1-3×	2,R	-	-	2	2/S	0.25/I	0.5/WT	1/NWT	0.03/S	0.03/S	0.12/S	0.5/WT	0.12	
110	F3632	13J3039	SRR23915643	2013	M	47	Medical	No	East	965	CCN115	Non-clade 4	Other	Diploid	-	-	1,2	-	-	2	2/S	0.12/S	0.25/WT	0.5/NWT	0.03/S	0.03/S	0.015/S	1/WT	0.06	
111	F3648	14HB045	SRR23915634	2014	F	81	Medical	Yes	North	343	CCN45	Non-clade 4	Other	Diploid	-	-	1,3	-	-	2	0.5/S	0.03/S	0.06/WT	0.06/WT	0.03/S	0.015/S	0.03/S	1/WT	0.06	
112	F3772	14AH086	SRR23915316	2014	F	75	Medical	Yes	East	343	CCN45	Non-clade 4	Other	Diploid	-	-	3,5,R	-	-	2	2/S	0.25/I	0.25/WT	0.25/WT	0.03/S	0.03/S	0.06/S	1/WT	0.12	
113	F3766	14AH074	SRR23915319	2014	M	34	Surgical	Yes	East	343	CCN45	Non-clade 4	Other	Diploid	-	-	1,3,R	-	-	2	2/S	0.12/S	0.12/WT	0.25/WT	0.03/S	0.03/S	0.06/S	1/WT	0.12	
114	F3684	16JD132	SRR23915412	2016	M	79	ICU	No	Northeast	343	CCN45	Non-clade 4	Other	Diploid	-	-	1,3,R	-	-	2	0.5/S	0.015/S	0.06/WT	0.03/WT	0.03/S	0.015/S	0.06/S	0.5/WT	0.12	
115	F4136	11XY007	SRR23915373	2011	M	30	Surgical	Yes	Central	343	CCN45	Non-clade 4	Other	Diploid	-	-	1,3,4,6,R	-	-	2	2/S	0.12/S	0.25/WT	0.25/WT	0.03/S	0.015/S	0.015/S	1/WT	0.06	
116	F3470	15HB055	SRR23915330	2015	M	28	Medical	Yes	North	343	CCN45	Non-clade 4	Other	Diploid	-	-	3,R	-	-	2	2/S	0.12/S	0.25/WT	0.25/WT	0.03/S	0.03/S	0.06/S	1/WT	0.12	
117	4986	21GZ1418	SRR38060774	2021	M	65	Surgical	No	South	12026	CCN57	Non-clade 4	Other	Diploid	-	-	1,3,4,R	V519L	Heterozygous	2	0.5/S	0.03/S	0.06/WT	0.03/WT	0.06/S	0.03/S	0.03/S	0.5/WT	≤0.06	Yes
118	F3460	15H1441	SRR23915336	2015	M	0	Other wards	Yes	Northeast	184	CCN6	Non-clade 4	Other	Diploid	-	-	1,6	-	-	2	1/S	0.06/S	0.12/WT	0.06/WT	0.03/S	0.03/S	0.015/S	1/WT	0.06	
119	F3346	14SJ111	SRR23915478	2014	F	3	Medical	Yes	Northeast	184	CCN6	Non-clade 4	Other	Diploid	-	chr 4-pos 1-3.5×	1,4,6	-	-	2	2/S	0.5/I	0.5/WT	0.5/NWT	0.03/S	0.03/S	0.03/S	1/WT	0.06	
120	F4078	14H1397	SRR23915260	2014	F	52	Surgical	No	Northeast	184	CCN6	Non-clade 4	Other	Diploid	-	-	1,6	-	-	2	0.5/S	0.06/S	0.06/WT	0.06/WT	0.015/S	0.03/S	0.015/S	0.5/WT	0.06	
121	F4102	15LJ009	SRR23915574	2015	F	70	Other wards	Yes	Northwest	10095	CCN6	Non-clade 4	Other	Diploid	chr 6-1.5×	-	1,2,4,6	-	-	2	2/S	0.12/S	0.12/WT	0.12/WT	0.06/S	0.015/S	0.015/S	1/WT	0.06	
122	F3938	13J2011	SRR23915469	2013	M	69	Surgical	Yes	East	184	CCN6	Non-clade 4	Other	Diploid	-	-	1,5,6	-	-	2	1/S	0.12/S	0.25/WT	0.12/WT	0.03/S	0.03/S	0.03/S	0.5/WT	0.06	
123	F4072	14AH089	SRR23915263	2014	M	48	Medical	No	East	10092	CCN6	Non-clade 4	Other	Diploid	-	-	1,2,3,4,6,R	-	-	2	2/S	0.12/S	0.12/WT	0.12/WT	0.03/S	0.03/S	0.06/S	1/WT	0.06	
124	F3370	15GH430	SRR23915559	2015	M	37	Outpatient/Emergency	Yes	North	184	CCN6	Non-clade 4	Other	Diploid	-	-	1,2,3,4,6,R	-	-	2	0.5/S	0.03/S	0.03/WT	0.06/WT	0.03/S	0.015/S	0.015/S	0.25/WT	0.06	
125	F3606	14H1401	SRR23915512	2014	M	31	Surgical	Yes	Northeast	184	CCN6	Non-clade 4	Other	Diploid	-	-	1,6	-	-	2	1/S	0.06/S	0.06/WT	0.06/WT	0.015/S	0.03/S	0.015/S	1/WT	0.06	
126	F3806	14F1052	SRR23915626	2014	F	58	Surgical	No	East	184	CCN6	Non-clade 4	Other	Diploid	-	-	1,6	-	-	2	1/S	0.06/S	0.12/WT	0.06/WT	0.03/S	0.03/S	0.03/S	1/WT	0.06	
127	F3692	14JD042	SRR23915409	2014	F	57	Medical	No	Northeast	184	CCN6	Non-clade 4	Other	Diploid	-	-	1,6	-	-	2	2/S	0.12/S	0.12/WT	0.12/WT	0.03/S	0.03/S	0.25/S	1/WT	0.06	
128	F3842	15A1060	SRR23915608	2015	M	61	Surgical	No	Northwest	184	CCN6	Non-clade 4	Other	Diploid	-	-	1,6	-	-	2	2/S	0.12/S	0.12/WT	0.12/WT	0.06/S	0.03/S	0.12/S	0.5/WT	0.12	
129	F9505	09HX016	SRR23915246	2009	M	46	ICU	Yes	Southwest	184	CCN6	Non-clade 4	Other	Diploid	-	-	1,4,6	-	-	2	1/S	0.03/S	0.06/WT	0.06/WT	0.03/S	0.03/S	0.03/S	0.5/WT	≤0.06	
130	F3382	15Z2157	SRR23915237	2015	M	45	ICU	Yes	East	184	CCN6	Non-clade 4	Other	Diploid	-	-	1,6	-	-	2	1/S	0.06/S	0.12/WT	0.06/WT	0.03/S	0.06/S	0.12/S	1/WT	0.06	
131	F9497	13H1265	SRR23915438	2013	M	59	Medical	Yes	Northeast	139	CCN6	Non-clade 4	Other	Diploid	-	-	3,5,6,R	-	-	2	2/S	0.12/S	0.25/WT	0.25/WT	0.03/S	0.03/S	0.03/S	1/WT	0.06	
132	F3726	16CH128	SRR23915501	2016	M	68	Medical	No	East	139	CCN6	Non-clade 4	Other	Diploid	-	-	1,3,6,R	-	-	2	2/S	0.12/S	0.12/WT	0.12/WT	0.06/S	0.03/S	0.12/S	0.5/WT	0.12	
133	F4016	14PU828	SRR23915596	2014	M	61	ICU	No	North	139	CCN6	Non-clade 4	Other	Diploid	-	-	1,2,3,R	-	-	2	1/S	0.06/S	0.12/WT	0.25/WT	0.03/S	0.03/S	0.03/S	1/WT	0.12	
134	F3754	15A1044	SRR23915485	2015	F	41	Surgical	No	Northwest	401	CCN6	Non-clade 4	Other	Diploid	-	chr 4-pos 1-4×	1,2,3,4,R	-	-	2	1/S	0.12/S	0.12/WT	0.25/WT	0.03/S	0.03/S	0.03/S	1/WT	0.12	
135	F3596	15GF026	SRR23915516	2015	M	34	Medical	Yes	South	139	CCN6	Non-clade 4	Other	Diploid	-	-	1,2,3,6,R	-	-	2	0.12/S	0.008/S	0.015/WT	0.03/WT	0.03/S	0.03/S	0.03/S	0.5/WT	0.06	
136	Z1848	21JS1058	SRR38060773	2021	M	83	ICU	Yes	East	139	CCN6	Non-clade 4	Other	Diploid	-	-	3,6,R	-	-	2	1/S	0.06/S	0.12/WT	0.12/WT	0.06/S	0.06/S	0.12/S	2/WT	≤0.06	Yes
137	Z1800	21JS1010	SRR38060772	2021	M	83	ICU	No	East	139	CCN6	Non-clade 4	Other	Diploid	-	-	3,6,R	-	-	2	2/S	0.12/S	0.12/WT	0.12/WT	0.12/S	0.06/S	0.12/S	1/WT	≤0.06	Yes
138	F3130	15A1074	SRR23915679	2015	F	0	Other wards	Yes	Northwest	139	CCN6	Non-clade 4	Other	Diploid	-	-	1,2,3,4,6,R	-	-	2	0.25/S	0.008/S	0.03/WT	0.015/WT	0.03/S	0.015/S	0.015/S	0.5/WT	0.06	
139	Z5359	20T1408	SRR38060771	2020	M	48	Surgical	No	North	139	CCN6	Non-clade 4	Other	Diploid	chr 6-0.5×	pos 2-0.5×, chr 4-pos	1,2,3,6,R	-	-	2	1/S	0.06/S	0.12/WT	0.12/WT	0.03/S	0.06/S	0.12/S	1/WT	0.25	Yes
140	Z5338	20T1387	SRR38060770	2020	M	48	Surgical	No	North	139	CCN6	Non-clade 4	Other	Diploid	-	-	1,2,3,6,R	-	-	2	1/S	0.06/S	0.12/WT	0.12/WT	0.06/S	0.06/S	0.12/S	1/WT	0.12	Yes
141	5029	21GZ1461	SRR38060769	2021	M	34	Medical	No	South	139	CCN6	Non-clade 4	Other	Diploid	-	-	1,2,3,6,R	-	-	2	0.5/S	0.015/S	≤0.015/WT	0.015/WT	0.03/S	0.03/S	0.03/S	0.5/WT	≤0.06	Yes
142	2668	21PU1825	SRR38060768	2021	F	77	Outpatient/Emergency	No	North	139	CCN6	Non-clade 4	Other	Diploid	-	-	1,2,3,4,6,R	-	-	2	0.5/S	0.03/S	0.12/WT	0.06/WT	0.03/S	0.03/S	0.12/S	0.5/WT	≤0.06	Yes
143	F3418	15LR275	SRR23915539	2015	F	82	ICU	Yes	Northeast	10023	CCN83	Non-clade 4	Other	Diploid	-	-	R	-	-	2	1/S	0.12/S	0.25/WT	0.25/WT	0.03/S	0.03/S	0.03/S	1/WT	0.12	
144	F3950	14SS013	SRR23915462	2014	M	42	Medical	Yes	North	10082	CCN84	Non-clade 4	Other	Diploid	-	-	R	-	-	2	1/S	0.06/S	0.12/WT	0.12/WT	0.03/S	0.03/S	0.12/S	1/WT	0.06	
145	F9471	09BD017	SRR23915356	2009	F	76	Surgical	No	North	10102	CCN22	Non-clade 4	Other	Diploid	-	-	1,2,4,R	-	-	2	0.5/S	0.03/S	0.12/WT	0.06/WT	0.03/S	0.03/S	0.03/S	1/WT	0.06	
146	F3834	16SD251	SRR23915612	2016	F	63	ICU	No	East	10071	CCN22	Non-clade 4	Other	Diploid	-	-	1,2,4,5,R	-	-	2	1/S	0.06/S	0.12/WT	0.12/WT	0.06/S	0.03/S	0.06/S	1/WT	0.06	
147	F3832	16SJ220	SRR23915613	2016	F	78	Outpatient/Emergency	Yes	Northeast	10070	CCN85	Non-clade 4	Other	Diploid	-	chr 6-pos 1-2×	1,2,3,4,6,R	-	-	2	1/S	0.03/S	0.12/WT	0.06/WT	0.06/S	0.06/S	0.12/S	0.5/WT	0.06	
148	F3536	15JS394	SRR23915665	2015	M	62	Surgical	Yes	East	211	CCN86	Non-clade 4	Other	Diploid	-	-	5,R	-	-	2	0.5/S	0.03/S	0.06/WT	0.03/WT	0.06/S	0.03/S	0.015/S	0.25/WT	0.06	
149	3022	21SJ692	SRR38060766	2021	F	50	Medical	Yes	Northeast	12023	CCN47	Non-clade 4	Other	Diploid	-	-	High	F,R245K,K344T,V362	Heterozygous	2	2/S	0.12/S	0.25/WT	0.25/NWT	0.06/S	0.06/S	0.12/S	2/WT	0.25	Yes
150	3694	21XH694	SRR38060765	2021	M	68	Surgical	No	Central	1348	CCN8	Non-clade 4	Other	Diploid	-	-	2,3,6,R	-	-	2	2/S	0.06/S	0.12/WT	0.12/WT	0.06/S	0.03/S	0.12/S	1/WT	≤0.06	Yes
151	4863	21GZ1295	SRR38060764	2021	F	27	Surgical	No	South	346	CCN8	Non-clade 4	Other	Diploid	-	-	1,2,4,6,R	-	-	2	2/S	0.06/S	0.12/WT	0.12/WT	0.03/S	0.03/S	0.03/S	0.5/WT	≤0.06	Yes
152	3667	21XH667	SRR38060763	2021	F	49	ICU	Yes	Central	1348	CCN8	Non-clade 4	Other	Diploid	-	-	3,4,5,6,R	-	-	2	2/S	0.12/S	0.25/WT	0.25/NWT	0.06/S	0.03/S	0.12/S	1/WT	≤0.06	Yes
153	2693	21PU1850	SRR38060762	2021	M	80	Outpatient/Emergency	No	North	346	CCN8	Non-clade 4	Other	Diploid	-	-	1,3,R	-	-	2	0.5/S	0.015/S	≤0.015/WT	0.015/WT	0.015/S	0.03/S	0.06/S	0.25/WT	≤0.06	Yes
154	Z6011	20HS1195	SRR38060761	2020	M	78	Medical	No	East	346	CCN8	Non-clade 4	Other	Diploid	-	pos 4-1.5×, chr 2-pos	4,6,R	-	-	2	8/R	0.25/I	0.5/WT	0.5/NWT	0.03/S	0.03/S	≤0.015/S	1/WT	≤0.06	Yes
155	Z5997	20HS1181	SRR38060760	2020	M	78	Medical	No	East	346	CCN8	Non-clade 4	Other	Diploid	chr 2-1.5×, chr R-1.5	chr 2-pos 3-2×	4,6,R	-	-	2	4/SDD	0.12/S	0.5/WT	0.5/NWT	0.12/S	0.03/S	0.12/S	1/WT	≤0.06	Yes
156	F4110	15PU1008	SRR23915569	2015	M	35	Outpatient/Emergency	No	North	10087	CCN77	Non-clade 4	Other	Diploid	-	-	R	-	-	2	1/S	0.12/S	0.25/WT	0.25/WT	0.06/S	0.06/S	0.12/S	1/WT	0.06	
157	F4018	15PU1032	SRR23915595	2015	M	35	Outpatient/Emergency	No	North	10087	CCN77	Non-clade 4	Other	Diploid	-	-	R	-	-	2	1/S	0.12/S	0.25/WT	0.25/WT	0.03/S	0.06/S	0.12/S	1/WT	0.06	
158	F3970	15LJ006	SRR23915291	2015	M	36	Other wards	Yes	Northwest	639	CCN78	Non-clade 4	Other	Diploid	-	-	4,R	-	-	2	2/S	0.5/I	0.5/WT	0.5/NWT	0.03/S	0.03/S	0.03/S	1/WT	0.06	
159	F4122	16PU1137	SRR23915563	2016	F	66	ICU	No	North	639	CCN78	Non-clade 4	Other	Diploid	-	-	5,R	-	-	2	4/SDD	0.25/I	0.25/WT	0.25/WT	0.015/S	0.03/S	0.015/S	1/WT	0.06	
160	F4084	16HN033	SRR23915257	2016	M	20	Medical	Yes	Central	639	CCN78	Non-clade 4	Other	Diploid	-	-	5,R	-	-	2	2/S	0.25/I	0.5/WT	0.5/NWT	0.06/S	0.03/S	0.12/S	1/WT	0.06	
161	F3406	14GX023	SRR23915543	2014	M	54	Medical	Yes	South	10021	CCN79	Non-clade 4	Other	Diploid	-	-	1	-	-	2	4/SDD	0.25/I	0.25/WT	0.5/NWT	0.03/S	0.015/S	0.06/S	1/WT	0.06	
162	F4066	15GH553	SRR23915267	2015	M	72	Outpatient/Emergency	No	North	385	CCN21	Non-clade 4	Other	Diploid	-	-	1,4,6,R	-	-	2	4/SDD	0.25/I	0.25/WT	0.25/WT	0.03/S	0.03/S	0.12/S	0.5/WT	0.06	
163	F3974	15NX171	SRR23915289	2015	M	71	Surgical	No	Northwest	10083	CCN21	Non-clade 4	Other	Diploid	-	chr 4-pos 1-3×	1,R	-	-	2	2/S	0.5/I	0.5/WT	0.25/WT	0.03/S	0.03/S	0.03/S	1/WT	0.06	
164	F3668	16GY024	SRR23915421	2016	M	68	Surgical	No	Southwest	10052	CCN80	Non-clade 4	Other	Diploid	-	-	1,2,4,R	-	-	2	2/S	0.12/S	0.12/WT	0.25/WT	0.03/S	0.06/S	0.06/S	0.5/WT	0.06	
165	F3994	15HZ039	SRR23915278	2015	M	63	ICU	Yes	Central	10086	CCN81	Non-clade 4	Other	Diploid	-	-	1,3,4,6,R	-	-	2	4/SDD	0.5/I	0.5/WT	0.5/NWT	0.015/S	0.03/S	0.015/S	1/WT	0.06	
166	F3784	16TJ755	SRR23915309	2016	M	46	ICU	Yes	Central	10063	CCN82	Non-clade 4	Other	Diploid	-	chr 4-pos 1-5.5×	1,R	Y132F, S154F	Heterozygous	6	256/R	8/R	1/NWT	1/NWT	0.03/S	0.06/S	0.12/S	1/WT	0.06	
167	F3354	16CH188	SRR23915601	2016	M	47	Medical	No	East	10013	CCN136	Non-clade 4	Other	Diploid	-	-	-	-	-	2	1/S	0.12/S	0.25/WT	0.25/WT	0.25/S	0.03/S	0.12/S	0.5/WT	0.12	
168	F3998	15LZ090	SRR23915603	2015	M	48	Surgical	Yes	Northwest	482	CCN34	Non-clade 4	Other	Diploid	-	-	R	-	-	2	2/S	0.06/S	0.12/WT	0.12/WT	0.03/S	0.03/S	0.015/S	1/WT	0.06	
169	F3634	14HB037	SRR23915642	2014	F	61	Medical	Yes	North	482	CCN34	Non-clade 4	Other	Diploid	chr 6-1.5×	-	R	-	-	2	1/S	0.06/S	0.12/WT	0.25/WT	0.03/S	0.015/S	0.03/S	0.5/WT	0.06	
170	F3440	16HN017	SRR23915345	2016	F	0	ICU	Yes	Central	482	CCN34	Non-clade 4	Other	Diploid	-	-	3,R	-	-	2	2/S	0.12/S	0.5/WT	1/NWT	0.06/S	0.03/S	0.06/S	0.5/WT	0.06	
171	F3144	16GY014	SRR23915678	2016	M	74	ICU	No	Southwest	10003	CCN34	Non-clade 4	Other	Diploid	-	-	4,R	-	-	2	0.25/S	0.008/S	0.015/WT	0.015/WT	0.03/S	0.015/S	0.015/S	0.5/WT	0.06	
172	F3554	15HX815	SRR23915656	2015	F	47	Medical	No	Southwest	346	CCN8	Non-clade 4	Other	Diploid	-	-	2,6,R	-	-	2	1/S	0.06/S	0.12/WT	0.12/WT	0.03/S	0.015/S	0.03/S	1/WT	0.06	
173	F3508	15Z2148	SRR23915424	2015	F	14	Medical	Yes	East	346	CCN8	Non-clade 4	Other	Diploid	-	chr 4-pos 1-2×	1,2,3,R	-	-	2	1/S	0.03/S	0.12/WT	0.06/WT	0.015/S	0.015/S	0.015/S	1/WT	0.06	
174	F4132	14TJ531	SRR23915558	2014	F	61	Medical	No	Central	346	CCN8	Non-clade 4	Other	Diploid	-	-	1,2,4,6,R	-	-	2	2/S	0.06/S	0.12/WT	0.12/WT	0.03/S	0.03/S	0.12/S	1/WT	0.06	
175	F3838	15K1037	SRR23915610	2015	F	79	ICU	No	Southwest	346	CCN8	Non-clade 4	Other	Diploid	-	-	1,2,6,R	-	-	2	1/S	0.06/S	0.12/WT	0.12/WT	0.015/S	0.015/S	0.015/S	0.5/WT	0.06	
176	F3256	16nx232	SRR23915635	2016	M	82	Medical	Yes	Northwest	346	CCN8	Non-clade 4	Other	Diploid	-	-	1,2,5,6,R	-	-	2	0.5/S	0.12/S	0.12/WT	0.06/WT	0.5/I	0.015/S	0.06/S	0.25/WT	0.12	
177	F3552	15LZ085	SRR23915657	2015	M	43	Medical	Yes	Northwest	346	CCN8	Non-clade 4	Other	Diploid	-	-	2,4,6,R	-	-	2	2/S	0.06/S	0.25/WT	0.25/WT	0.06/S	0.03/S	0.12/S	1/WT	0.06	
178	F3220	16K1097	SRR23915524	2016	F	71	ICU	Yes	Southwest	346	CCN8	Non-clade 4	Other	Diploid	-	-	2,4,5,6,R	-	-	2	128/R	8/R	16/NWT	8/NWT	0.06/S	0.015/S	0.06/S	1/WT	0.06	
179	F3538	15JS477	SRR23915664	2015	M	28	Outpatient/Emergency	No	East	346	CCN8	Non-clade 4	Other	Diploid	-	-	1,4,5,6,R	-	-	2	2/S	0.12/S	0.25/WT	0.12/WT	0.25/S	0.06/S	0.12/S	0.5/WT	0.06	
180	F4006	15CZ005	SRR23915599	2015	F	75	Outpatient/Emergency	Yes	East	346	CCN8	Non-clade 4	Other	Diploid	-	-	1,2,4,6,R	-	-	2	2/S	0.25/I	0.5/WT	0.5/NWT	0.03/S	0.03/S	0.12/S	1/WT	0.06	
181	F3982	15DZ027	SRR23915284	2015	F	79	Medical	Yes	Northeast	346	CCN8	Non-clade 4	Other	Diploid	-	-	1,4,6,R	-	-	2	1/S	0.06/S	0.12/WT	0.25/WT	0.06/S	0.015/S	0.12/S	1/WT	0.06	
182	F3948	14SS011	SRR23915463	2014	F	52	Medical	Yes	North	346	CCN8	Non-clade 4	Other	Diploid	-	-	1,2,4,6,R	-	-	2	2/S	0.12/S	0.25/WT	0.25/WT	0.03/S	0.03/S	0.015/S	1/WT	0.06	
183	F3646	14HB040	SRR23915636	2014	M	24	Surgical	No	North	346	CCN8	Non-clade 4	Other	Diploid	-	-	1,2,4,6,R	-	-	2	1/S	0.03/S	0.06/WT	0.12/WT	0.03/S	0.015/S	0.015/S	1/WT	0.06	
184	F3194	14K1023	SRR23915677	2014	M	62	ICU	No	Southwest	346	CCN8	Non-clade 4	Other	Diploid	-	-	1,2,3,4,6,R	-	-	2	0.25/S	0.008/S	0.03/WT	0.03/WT	0.03/S	0.015/S	0.06/S	0.5/WT	64	
185	Z5614	21H4689	SRR38060759	2021	F	53	Medical	No	Northeast	12001	CCN8	Non-clade 4	Other	Diploid	-	-	1,2,4,6,R	-	-	2	2/S	0.06/S	0.12/WT	0.06/WT	0.03/S	0.03/S	0.03/S	1/WT	≤0.06	Yes
186	Z5608	21H4683	SRR38060806	2021	F	53	Medical	No	Northeast	12001	CCN8	Non-clade 4	Other	Diploid	-	-	1,2,4,6,R	-	-	2	2/S	0.06/S	0.12/WT	0.12/WT	0.03/S	0.015/S	≤0.015/S	1/WT	≤0.06	Yes
187	Z3138	21SJ808	SRR38060805	2021	M	64	Other wards	No	Northeast	341	CCN8	Non-clade 4	Other	Diploid	-	-	R	-	-	2	2/S	0.12/S	0.25/WT	0.25/NWT	0.06/S	0.03/S	0.06/S	1/WT	0.12	Yes
188	Z3134	21SJ804	SRR38060803	2021	M	64	Other wards	No	Northeast	341	CCN8	Non-clade 4	Other	Diploid	-	-	R	-	-	2	2/S	0.06/S	0.25/WT	0.12/WT	0.03/S	0.03/S	≤0.015/S	2/WT	0.12	Yes
189	Z3047	21SJ717	SRR38060802	2021	M	12	Medical	Yes	Northeast	346	CCN8	Non-clade 4	Other	Diploid	-	-	2,4,5,R	-	-	2	2/S	0.12/S	0.25/WT	0.25/NWT	0.06/S	0.03/S	0.12/S	1/WT	≤0.06	Yes
190	Z3001	20SJ670	SRR38060801	2020	M	12	Medical	Yes	Northeast	346	CCN8	Non-clade 4	Other	Diploid	-	-	4,5,R	-	-	2	2/S	0.12/S	0.25/WT	0.25/NWT	0.06/S	0.03/S	0.12/S	1/WT	≤0.06	Yes
191	3631	21XH631	SRR38060800	2021	F	57	ICU	No	Central	1515	CCN51	Non-clade 4	Other	Diploid	-	-	5	Y132F,S154F,Y257H	Heterozygous	2	32/R	1/R	0.25/WT	0.25/NWT	0.06/S	0.06/S	0.25/S	1/WT	0.12	Yes
192	F3928	16PU1072	SRR23915474	2016	M	54	Surgical	Yes	North	10078	CCN14	Non-clade 4	Other	Diploid	-	-	6,R	-	-	2	1/S	0.06/S	0.25/WT	0.25/WT	0.03/S	0.06/S	0.03/S	1/WT	0.06	
193	F9477	12BD249	SRR23915459	2012	N/A	N/A	Other wards	No	North	490	CCN14	Non-clade 4	Other	Diploid	-	-	R	-	-	2	1/S	0.12/S	0.25/WT	0.25/WT	0.06/S	0.03/S	0.12/S	1/WT	≤0.06	
194	F3810	15K1042	SRR23915625	2015	F	69	Surgical	No	Southwest	10064	CCN126	Non-clade 4	Other	Diploid	-	chr 4-pos 1-1.5×	1,4,R	-	-	2	1/S	0.06/S	0.12/WT	0.25/WT	0.03/S	0.03/S	0.015/S	1/WT	0.06	
195	F3752	15A1042	SRR23915486	2015	M	71	Medical	No	Northwest	10058	CCN127	Non-clade 4	Other	Diploid	-	-	3,R	-	-	2	2/S	0.12/S	0.25/WT	0.5/NWT	0.03/S	0.03/S	0.12/S	1/WT	0.06	
196	F3758	15Z2116	SRR23915323	2015	M	90	ICU	No	East	10059	CCN128	Non-clade 4	Other	Diploid	-	-	6,R	-	-	2	2/S	0.12/S	0.25/WT	0.25/WT	0.03/S	0.03/S	0.06/S	1/WT	0.06	
197	F3712	14SS002	SRR23915398	2014	F	44	Medical	Yes	North	10055	CCN13	Non-clade 4	Other	Diploid	-	-	1,2,3,5,R	-	-	2	0.25/S	0.015/S	0.06/WT	0.03/WT	0.03/S	0.03/S	0.015/S	1/WT	0.06	
198	F4098	12J3003	SRR23915576	2012	M	41	Medical	Yes	East	1277	CCN10	Non-clade 4	Other	Diploid	-	-	4,6,R	-	-	2	1/S	0.06/S	0.25/WT	0.25/WT	0.03/S	0.03/S	0.03/S	0.5/WT	0.06	
199	F3880	16TJ825	SRR23915381	2016	M	72	ICU	Yes	Central	1277	CCN10	Non-clade 4	Other	Diploid	-	-	4,6,R	-	-	2	2/S	0.12/S	0.25/WT	0.5/NWT	0.03/S	0.03/S	0.03/S	2/WT	0.06	
200	F9515	12HX326	SRR23915235	2012	F	20	ICU	Yes	Southwest	1277	CCN10	Non-clade 4	Other	Diploid	-	-	R	-	-	2	2/S	0.06/S	0.25/WT	0.25/WT	0.03/S	0.03/S	0.06/S	0.5/WT	≤0.06	
201	F4106	12NX083	SRR23915572	2012	M	22	Surgical	No	Northwest	1277	CCN10	Non-clade 4	Other	Diploid	-	-	4,R	-	-	2	2/S	0.12/S	0.25/WT	0.25/WT	0.03/S	0.03/S	0.015/S	1/WT	0.06	
202	F3178	15H4197	SRR23915343	2015	M	85	ICU	Yes	Northeast	1277	CCN10	Non-clade 4	Other	Diploid	-	-	R	-	-	2	0.5/S	0.008/S	0.03/WT	0.015/WT	0.06/S	0.008/S	0.12/S	0.5/WT	0.12	
203	F9504	09HX015	SRR23915247	2009	M	41	Surgical	Yes	Southwest	332	CCN129	Non-clade 4	Other	Diploid	-	-	6,R	-	-	2	2/S	0.06/S	0.12/WT	0.12/WT	0.03/S	0.03/S	0.06/S	1/WT	≤0.06	
204	F3850	15FJ456	SRR23915396	2015	M	28	Medical	Yes	East	332	CCN129	Non-clade 4	Other	Diploid	-	-	R	-	-	2	2/S	0.25/I	0.25/WT	0.25/WT	0.03/S	0.06/S	0.12/S	0.5/WT	0.06	
205	F3770	14AH083	SRR23915317	2014	F	19	Medical	Yes	East	332	CCN129	Non-clade 4	Other	Diploid	-	-	R	-	-	2	1/S	0.06/S	0.25/WT	0.25/WT	0.03/S	0.03/S	0.03/S	1/WT	0.06	
206	F3700	15H1437	SRR23915404	2015	M	35	Surgical	Yes	Northeast	183	CCN130	Non-clade 4	Other	Diploid	-	chr 4-pos 1-3×	4,R	-	-	2	4/SDD	0.5/I	0.25/WT	0.5/NWT	0.03/S	0.03/S	0.03/S	1/WT	0.06	
207	F3542	15JS410	SRR23915662	2015	M	61	Medical	Yes	East	183	CCN130	Non-clade 4	Other	Diploid	-	-	1,6,R	-	-	2	1/S	0.06/S	0.12/WT	0.12/WT	0.03/S	0.03/S	0.12/S	1/WT	0.06	
208	F3944	14AH068	SRR23915465	2014	F	50	Medical	Yes	East	183	CCN130	Non-clade 4	Other	Diploid	-	-	4,R	-	-	2	4/SDD	0.5/I	0.5/WT	0.5/NWT	0.03/S	0.03/S	0.06/S	1/WT	0.06	
209	F3764	14AH069	SRR23915320	2014	F	50	Medical	Yes	East	183	CCN130	Non-clade 4	Other	Diploid	-	chr 4-pos 1-2.5×	4,R	-	-	2	4/SDD	0.5/I	0.5/WT	0.5/NWT	0.03/S	0.03/S	0.015/S	1/WT	0.06	

210	F9516	12HX402	SRR23915234	2012	M	74	Other wards	No	Southwest	232	CCN131	Non-clade 4	Other	Diploid	-	-	1,2,R	-	-	2	2/S	0.06/S	0.25/WT	0.12/WT	0.03/S	0.03/S	0.03/S	1/WT	≤0.06	
211	F3860	16DL094	SRR23915391	2016	M	69	Outpatient/Emergency	No	Northeast	1175	CCN132	Non-clade 4	Other	Diploid	-	-	2,6,R	-	-	2	1/S	0.12/S	0.25/WT	0.25/WT	0.03/S	0.03/S	0.12/S	0.5/WT	0.06	
212	F3458	15GX110	SRR23915337	2015	F	3	Other wards	Yes	South	1175	CCN132	Non-clade 4	Other	Diploid	-	-	6,R	-	-	2	2/S	0.12/S	0.25/WT	0.25/WT	0.03/S	0.03/S	0.12/S	0.5/WT	0.06	
213	F4040	15CQ319	SRR23915584	2015	M	76	Surgical	Yes	Southwest	10089	CCN15	Non-clade 4	Other	Diploid	-	-	High	-	-	2	1/S	0.06/S	0.12/WT	0.06/WT	0.06/S	0.03/S	0.12/S	0.5/WT	0.06	
214	F9519	12HX458	SRR23915231	2012	M	49	Medical	No	Southwest	10114	CCN15	Non-clade 4	Other	Diploid	-	-	High	-	-	2	2/S	0.06/S	0.12/WT	0.12/WT	0.03/S	0.03/S	0.12/S	1/WT	≤0.06	
215	F9510	10HX104	SRR23915241	2010	M	72	Surgical	No	Southwest	10112	CCN15	Non-clade 4	Other	Diploid	-	-	High	-	-	2	1/S	0.03/S	0.12/WT	0.12/WT	0.03/S	0.03/S	0.06/S	0.5/WT	0.06	
216	F4028	14LR209	SRR23915591	2014	F	69	Medical	Yes	Northeast	10054	CCN33	Non-clade 4	Other	Diploid	-	-	High	-	-	2	0.5/S	0.03/S	0.06/WT	0.06/WT	0.03/S	0.03/S	0.06/S	1/WT	0.06	
217	F3976	14LR210	SRR23915288	2014	F	69	Medical	No	Northeast	10084	CCN33	Non-clade 4	Other	Diploid	-	-	High	-	-	2	4/SDD	0.03/S	0.12/WT	0.06/WT	0.25/S	0.03/S	0.06/S	1/WT	0.06	
218	F4052	16GH562	SRR23915274	2016	F	83	ICU	No	North	10054	CCN33	Non-clade 4	Other	Diploid	-	-	High	-	-	2	1/S	0.12/S	0.12/WT	0.12/WT	0.12/S	0.06/S	0.12/S	0.5/WT	0.06	
219	F3730	15TJ717	SRR23915499	2015	M	46	Surgical	No	Central	10054	CCN33	Non-clade 4	Other	Diploid	-	-	High	-	-	2	2/S	0.06/S	0.12/WT	0.12/WT	0.25/S	0.03/S	0.25/S	1/WT	0.06	
220	F3696	14H1415	SRR23915407	2014	M	64	Surgical	Yes	Northeast	10054	CCN33	Non-clade 4	Other	Diploid	-	-	High	-	-	2	0.5/S	0.03/S	0.03/WT	0.03/WT	0.03/S	0.03/S	0.03/S	0.5/WT	0.06	
221	F3778	16K1104	SRR23915312	2016	M	0	ICU	Yes	Southwest	10062	CCN133	Non-clade 4	Other	Diploid	-	-	High	-	-	2	1/S	0.06/S	0.12/WT	0.06/WT	0.03/S	0.03/S	0.03/S	0.25/WT	0.06	
222	F3424	15GH474	SRR23915535	2015	F	56	Outpatient/Emergency	Yes	North	10026	CCN134	Non-clade 4	Other	Diploid	-	-	High	-	-	2	1/S	0.06/S	0.12/WT	0.06/WT	0.03/S	0.015/S	0.015/S	0.5/WT	0.06	
223	F3608	14H1402	SRR23915511	2014	F	54	Medical	Yes	Northeast	10046	CCN135	Non-clade 4	Other	Diploid	-	-	High	-	-	2	1/S	0.03/S	0.06/WT	0.06/WT	0.03/S	0.03/S	0.12/S	1/WT	0.06	
224	F3454	14Z2105	SRR23915339	2014	F	60	Surgical	No	East	848	CCN32	Non-clade 4	Other	Diploid	-	-	2,R	Y257H	Homozygous	2	4/SDD	0.25/I	0.5/WT	0.5/NWT	0.03/S	0.03/S	0.06/S	0.5/WT	0.06	
225	F9511	11HX129	SRR23915240	2011	M	47	Medical	No	Southwest	10113	CCN32	Non-clade 4	Other	Diploid	-	-	2,5,R	Y257H	Homozygous	2	8/R	1/R	0.5/WT	0.5/NWT	0.03/S	0.03/S	0.12/S	1/WT	0.06	
226	F3484	14HX766	SRR23915435	2014	F	24	ICU	Yes	Southwest	848	CCN32	Non-clade 4	Other	Diploid	-	-	2,R	Y257H	Homozygous	2	8/R	0.5/I	0.25/WT	0.5/NWT	0.015/S	0.015/S	0.015/S	1/WT	0.06	
227	F9513	11HX197	SRR23915238	2011	F	57	Medical	Yes	Southwest	848	CCN32	Non-clade 4	Other	Diploid	-	-	1,2,R	Y257H	Homozygous	2	8/R	0.5/I	0.25/WT	0.25/WT	0.03/S	0.015/S	0.03/S	0.5/WT	≤0.06	
228	F4070	14GX061	SRR23915264	2014	F	28	Medical	Yes	South	10091	CCN125	Non-clade 4	Other	Diploid	-	-	2,R	Y257H	Homozygous	2	16/R	2/R	1/NWT	1/NWT	0.015/S	0.03/S	0.06/S	1/WT	0.12	
229	4907	21GZ1339	SRR38060799	2021	M	62	Other wards	No	South	848	CCN32	Non-clade 4	Other	Diploid	-	-	2,4,R	Y257H	Homozygous	2	16/R	0.5/I	0.25/WT	0.5/NWT	0.03/S	0.06/S	0.06/S	0.5/WT	≤0.06	Yes
230	5031	21GZ1463	SRR38060798	2021	M	58	Surgical	No	South	1372	CCN26	Non-clade 4	Other	Diploid	-	-	1,2,4,R	-	-	2	1/S	0.06/S	0.12/WT	0.12/WT	0.06/S	0.03/S	0.12/S	1/WT	≤0.06	Yes
231	3133	21SJ803	SRR38060797	2021	F	24	Other wards	No	Northeast	12008	CCN18	Non-clade 4	Other	Diploid	-	-	1,R	-	-	2	1/S	0.12/S	0.12/WT	0.12/WT	0.06/S	0.06/S	0.12/S	1/WT	≤0.06	Yes
232	Z3643	21XH643	SRR38060796	2021	M	58	Surgical	Yes	Central	349	CCN2	Non-clade 4	Other	Diploid	-	-	1,6,R	-	-	2	2/S	0.06/S	0.12/WT	0.25/NWT	0.12/S	0.06/S	0.25/S	1/WT	≤0.06	Yes
233	Z3648	21XH648	SRR38060795	2021	M	58	Surgical	Yes	Central	12012	CCN2	Non-clade 4	Other	Diploid	Isochr 6p-2×	-	1,5,6,R	-	-	2	2/S	0.12/S	0.25/WT	0.25/NWT	0.06/S	0.03/S	0.12/S	1/WT	≤0.06	Yes
234	Z3644	21XH644	SRR38060794	2021	M	58	ICU	No	Central	349	CCN2	Non-clade 4	Other	Diploid	-	-	1,6,R	-	-	2	2/S	0.12/S	0.12/WT	0.12/WT	0.12/S	0.06/S	0.25/S	1/WT	≤0.06	Yes
235	2595	21PU1752	SRR38060792	2021	F	57	ICU	Yes	North	726	CCN24	Non-clade 4	Other	Diploid	chr R-1.5×	chr 1-pos 2-1.5×	2,3,6,R	-	-	2	4/SDD	0.06/S	0.12/WT	0.06/WT	0.06/S	0.06/S	0.25/S	1/WT	≤0.06	Yes
236	4932	21GZ1364	SRR38060791	2021	F	45	ICU	No	South	12009	CCN54	Non-clade 4	Other	Diploid	-	-	-	-	-	2	2/S	0.12/S	0.25/WT	0.12/WT	0.06/S	0.06/S	0.5/I	1/WT	≤0.06	Yes
237	F3550	15LZ083	SRR23915658	2015	M	70	Surgical	No	Northwest	1384	CCN120	Non-clade 4	Other	Diploid	-	-	1,2,4,R	-	-	2	2/S	0.12/S	0.25/WT	0.25/WT	0.03/S	0.03/S	0.03/S	1/WT	0.06	
238	F3426	15GH462	SRR23915534	2015	M	45	Outpatient/Emergency	No	North	1384	CCN120	Non-clade 4	Other	Diploid	-	-	R	-	-	2	0.25/S	0.015/S	0.06/WT	0.03/WT	0.06/S	0.015/S	0.03/S	0.5/WT	0.06	
239	F3562	15JS380	SRR23915531	2015	F	50	Medical	Yes	East	10030	CCN30	Non-clade 4	Other	Diploid	-	-	1,5,R	-	-	2	1/S	0.06/S	0.12/WT	0.12/WT	0.03/S	0.03/S	0.06/S	0.5/WT	0.06	
240	F3464	16DP080	SRR23915334	2016	F	62	Medical	No	Southwest	10030	CCN30	Non-clade 4	Other	Diploid	-	-	1,5,R	-	-	2	2/S	0.12/S	0.25/WT	0.25/WT	0.06/S	0.03/S	0.12/S	0.5/WT	0.06	
241	F3396	16DP104	SRR23915546	2016	F	62	Medical	Yes	Southwest	10019	CCN30	Non-clade 4	Other	Diploid	-	-	1,5,R	-	-	2	0.25/S	0.008/S	0.06/WT	0.03/WT	0.06/S	0.015/S	0.06/S	0.25/WT	0.12	
242	F4130	14SF033	SRR23915560	2014	M	66	Surgical	Yes	North	10097	CCN31	Non-clade 4	Other	Diploid	-	-	1,2,4,R	-	-	2	1/S	0.25/I	0.25/WT	0.25/WT	0.06/S	0.03/S	0.25/S	1/WT	0.06	
243	F3442	16HN015	SRR23915344	2016	M	59	ICU	Yes	Central	10014	CCN31	Non-clade 4	Other	Diploid	-	-	1,2,R	G464S	Heterozygous	2	16/R	1/R	16/NWT	1/NWT	0.06/S	0.03/S	0.12/S	1/WT	0.06	
244	F3356	16HN013	SRR23915590	2016	F	64	Surgical	No	Central	10014	CCN31	Non-clade 4	Other	Diploid	-	-	1,2,R	-	-	2	2/S	0.25/I	0.5/WT	0.25/WT	0.06/S	0.03/S	0.12/S	1/WT	0.06	
245	F3558	15JS391	SRR23915653	2015	M	46	Medical	Yes	East	10040	CCN121	Non-clade 4	Other	Diploid	-	chr 4-pos 1-4×	4,R	-	-	2	0.5/S	0.015/S	0.06/WT	0.03/WT	0.015/S	0.008/S	0.015/S	0.25/WT	0.06	
246	F3496	15FJ428	SRR23915429	2015	F	7	Outpatient/Emergency	Yes	East	1352	CCN122	Non-clade 4	Other	Diploid	-	-	R	-	-	2	2/S	0.25/I	0.5/WT	0.5/NWT	0.03/S	0.06/S	0.03/S	1/WT	0.06	
247	F3892	15DZ040	SRR23915374	2015	F	88	Outpatient/Emergency	Yes	Northeast	10075	CCN123	Non-clade 4	Other	Diploid	-	-	6,R	-	-	2	1/S	0.06/S	0.25/WT	0.25/WT	0.03/S	0.03/S	0.12/S	1/WT	0.06	
248	F4152	15Z2132	SRR23915366	2015	F	44	Surgical	Yes	East	10075	CCN123	Non-clade 4	Other	Diploid	-	-	1,R	-	-	2	1/S	0.06/S	0.12/WT	0.12/WT	0.03/S	0.015/S	0.03/S	1/WT	0.06	
249	F3918	16PU1094	SRR23915476	2016	F	61	ICU	Yes	North	10075	CCN123	Non-clade 4	Other	Diploid	-	-	1,R	-	-	2	256/R	8/R	1/NWT	1/NWT	0.06/S	0.03/S	0.06/S	2/WT	0.06	
250	F3510	15Z2158	SRR23915423	2015	F	77	Surgical	Yes	East	10035	CCN2	Non-clade 4	Other	Diploid	-	-	1,2,3,4,6,R	-	-	2	1/S	0.06/S	0.12/WT	0.12/WT	0.06/S	0.06/S	0.12/S	1/WT	0.06	
251	F3452	14Z2110	SRR23915340	2014	M	85	Medical	Yes	East	1378	CCN124	Non-clade 4	Other	Diploid	-	-	3,4	-	-	2	0.5/S	0.03/S	0.12/WT	0.12/WT	0.03/S	0.03/S	0.06/S	0.5/WT	0.06	
252	F4068	16GH555	SRR23915266	2016	F	73	Surgical	No	North	983	CCN116	Non-clade 4	Other	Diploid	-	-	1,2,3,R	-	-	2	2/S	0.12/S	0.25/WT	0.25/WT	0.03/S	0.03/S	0.12/S	0.5/WT	0.06	
253	F4124	16QL030	SRR23915562	2016	M	45	ICU	No	East	439	CCN13	Non-clade 4	Other	Diploid	-	chr 4-pos 1-1.5×	4,R	-	-	2	1/S	0.12/S	0.12/WT	0.12/WT	0.03/S	0.06/S	0.06/S	0.5/WT	0.06	
254	F4140	14XY232	SRR23915371	2014	F	58	Medical	Yes	Central	10099	CCN13	Non-clade 4	Other	Diploid	-	-	5,R	-	-	2	2/S	0.12/S	0.25/WT	0.25/WT	0.03/S	0.03/S	0.06/S	1/WT	0.06	
255	F3530	15HX814	SRR23915669	2015	M	46	ICU	Yes	Southwest	10039	CCN117	Non-clade 4	Other	Diploid	-	-	R	-	-	2	1/S	0.06/S	0.12/WT	0.12/WT	0.03/S	0.03/S	0.03/S	1/WT	0.06	
256	F3680	15Z2143	SRR23915414	2015	F	61	Surgical	No	East	197	CCN118	Non-clade 4	Other	Diploid	-	-	3,R	-	-	2	1/S	0.015/S	0.12/WT	0.06/WT	0.5/I	1/R	1/R	2/WT	0.06	
257	F3492	14SS001	SRR23915430	2014	F	31	Medical	Yes	North	520	CCN27	Non-clade 4	Other	Diploid	-	-	1,3,6,R	-	-	2	4/SDD	0.25/I	0.5/WT	0.5/NWT	0.03/S	0.03/S	0.03/S	1/WT	0.06	
258	F9472	10BD101	SRR23915355	2010	M	59	Outpatient/Emergency	No	North	10103	CCN27	Non-clade 4	Other	Diploid	-	-	3,R	-	-	2	2/S	0.12/S	0.5/WT	0.25/WT	0.06/S	0.03/S	0.12/S	0.5/WT	≤0.06	
259	F3366	14Z2112	SRR23915570	2014	M	66	ICU	No	East	520	CCN27	Non-clade 4	Other	Diploid	-	-	1,2,3,R	-	-	2	2/S	0.12/S	0.25/WT	0.25/WT	0.03/S	0.015/S	0.03/S	1/WT	0.06	
260	F4164	14ZZ140	SRR23915359	2014	M	11	Medical	Yes	Central	10101	CCN28	Non-clade 4	Other	Diploid	-	-	2,4,5,R	-	-	2	2/S	0.25/I	0.25/WT	0.25/WT	0.12/S	0.03/S	0.25/S	1/WT	0.12	
261	F3566	15PU990	SRR23915529	2015	F	47	Medical	Yes	North	10041	CCN28	Non-clade 4	Other	Diploid	-	-	R	-	-	2	1/S	0.12/S	0.25/WT	0.25/WT	0.03/S	0.03/S	0.015/S	1/WT	0.06	
262	F3422	15H4193	SRR23915536	2015	M	59	Surgical	No	Northeast	10025	CCN29	Non-clade 4	Other	Diploid	-	-	1,2,6,R	-	-	2	2/S	0.25/I	0.5/WT	0.5/NWT	0.015/S	0.03/S	0.03/S	1/WT	0.12	
263	F9520	12HX507	SRR23915230	2012	F	49	Medical	No	Southwest	10025	CCN29	Non-clade 4	Other	Diploid	-	-	1,R	-	-	2	2/S	0.12/S	0.25/WT	0.25/WT	0.06/S	0.03/S	0.12/S	1/WT	≤0.06	
264	F9493	12H1200	SRR23915443	2012	M	46	ICU	Yes	Northeast	10025	CCN29	Non-clade 4	Other	Diploid	-	-	1,R	-	-	2	2/S	0.06/S	0.25/WT	0.25/WT	0.03/S	0.03/S	0.03/S	0.5/WT	≤0.06	
265	F9502	09HX010	SRR23915250	2009	M	82	ICU	Yes	Southwest	10011	CCN0	Non-clade 4	Other	Diploid	-	-	1,2,3,4,R	-	-	2	1/S	0.06/S	0.25/WT	0.25/WT	0.03/S	0.015/S	0.015/S	1/WT	≤0.06	
266	F9506	10HX030	SRR23915245	2010	F	53	Surgical	Yes	Southwest	10011	CCN0	Non-clade 4	Other	Diploid	-	-	1,3,4,R	-	-	2	2/S	0.12/S	0.25/WT	0.25/WT	0.03/S	0.015/S	0.015/S	0.5/WT	≤0.06	
267	F3342	15GH467	SRR23915389	2015	M	17	Medical	Yes	North	10011	CCN0	Non-clade 4	Other	Diploid	-	-	1,3,4,R	-	-	2	1/S	0.06/S	0.12/WT	0.12/WT	0.03/S	0.015/S	0.015/S	0.5/WT	0.06	
268	F4134	11XY004	SRR23915557	2011	F	70	Surgical	No	Central	10098	CCN0	Non-clade 4	Other	Diploid	-	-	1,3,4,R	-	-	2	2/S	0.06/S	0.25/WT	0.25/WT	0.015/S	0.015/S	0.015/S	1/WT	0.06	
269	F3628	13J3032	SRR23915645	2013	F	78	ICU	Yes	East	10011	CCN0	Non-clade 4	Other	Diploid	-	-	1,R	-	-	2	1/S	0.06/S	0.25/WT	0.25/WT	0.03/S	0.015/S	0.015/S	1/WT	0.06	
270	F3732	15SJ163	SRR23915498	2015	F	47	Surgical	Yes	Northeast	10011	CCN0	Non-clade 4	Other	Diploid	-	-	1,2,R	-	-	2	2/S	0.25/I	0.5/WT	0.5/NWT	0.015/S	0.015/S	0.015/S	1/WT	0.06	
271	F3788	15SJ164	SRR23915308	2015	M	77	Surgical	Yes	Northeast	10011	CCN0	Non-clade 4	Other	Diploid	-	-	1,2,R	-	-	2	2/S	0.25/I	0.5/WT	0.5/NWT	0.015/S	0.03/S	0.015/S	1/WT	0.06	
272	F3480	14ZD331	SRR23915326	2014	M	39	Surgical	Yes	East	10011	CCN0	Non-clade 4	Other	Diploid	-	-	1,4,R	-	-	2	2/S	0.25/I	0.5/WT	0.5/NWT	0.03/S	0.015/S	0.03/S	1/WT	0.06	
273	F3762	15Z2129	SRR23915321	2015	M	47	ICU	Yes	East	10011	CCN0	Non-clade 4	Other	Diploid	-	-	1,6,R	-	-	2	1/S	0.06/S	0.12/WT	0.12/WT	0.03/S	0.03/S	0.06/S	1/WT	0.06	
274	F3398	16DP097	SRR23915545	2016	M	66	ICU	Yes	Southwest	426	CCN0	Non-clade 4	Other	Diploid	-	-	2,5,R	-	-	2	2/S	0.12/S	0.25/WT	0.25/WT	0.25/S	0.03/S	0.12/S	0.5/WT	0.06	
275	F3468	15HB052	SRR23915331	2015	F	59	Surgical	Yes	North	426	CCN0	Non-clade 4	Other	Diploid	-	-	2,5,R	-	-	2	1/S	0.03/S	0.12/WT	0.12/WT	0.03/S	0.008/S	0.015/S	1/WT	0.06	
276	F3800	14DZ011	SRR23915303	2014	F	58	Outpatient/Emergency	Yes	Northeast	426	CCN0	Non-clade 4	Other	Diploid	-	-	2,5,R	-	-	2	0.5/S	0.06/S	0.25/WT	0.25/WT	0.12/S	0.015/S	0.12/S	0.5/WT	0.06	
277	F9480	13BD273	SRR23915456	2013	F	52	Outpatient/Emergency	No	North	426	CCN0	Non-clade 4	Other	Diploid	-	-	2,4,5,R	-	-	2	2/S	0.12/S	0.25/WT	0.25/WT	0.06/S	0.03/S	0.12/S	1/WT	≤0.06	
278	4930	21GZ1362	SRR38060790	2021	F	61	Surgical	No	South	12021	CCN14	Non-clade 4	Other	Diploid	-	-	R	-	-	2	2/S	0.06/S	0.12/WT	0.12/WT	0.06/S	0.06/S	0.25/S	1/WT	≤0.06	Yes
279	2695	21PU1852	SRR38060789	2021	M	58	Outpatient/Emergency	No	North	1277	CCN10	Non-clade 4	Other	Diploid	-	-	1,R	-	-	2	1/S	0.06/S	0.12/WT	0.06/WT	0.06/S	0.06/S	0.12/S	0.5/WT	≤0.06	Yes
280	4923	21GZ1355	SRR38060788	2021	M	60	Other wards	No	South	12015	CCN19	Non-clade 4	Other	Diploid	-	-	4,6,R	-	-	2	2/S	0.12/S	0.12/WT	0.25/NWT	0.03/S	0.06/S	≤0.015/S	1/WT	≤0.06	Yes
281	5054	21GZ1486	SRR38060787	2021	M	82	Surgical	Yes	South	12027	CCN19	Non-clade 4	Other	Diploid	-	-	4,6,R	-	-	2	1/S	0.06/S	0.12/WT	0.12/WT	0.06/S	0.06/S	0.06/S	1/WT	≤0.06	Yes
282	4918	21GZ1350	SRR38060786	2021	M	68	ICU	No	South	12015	CCN19	Non-clade 4	Other	Diploid	-	-	4,6,R	-	-	2	1/S	0.06/S	0.12/WT	0.25/NWT	0.06/S	0.06/S	≤0.015/S	0.5/WT	≤0.06	Yes
283	Z4341	21QH685	SRR38060785	2021	M	67	Surgical	No	East	520	CCN27	Non-clade 4	Other	Diploid	-	-	R	-	-	2	4/SDD	0.25/I	1/NWT	1/NWT	0.06/S	0.06/S	0.25/S	1/WT	≤0.06	Yes
284	F3502	15G1194	SRR23915426	2015	M	78	ICU	No	South	10034	CCN119	Non-clade 4	Other	Diploid	-	-	1,3,4,R	Y132F, S154F	Heterozygous	2	32/R	2/R	0.5/WT	0.5/NWT	0.03/S	0.03/S	0.03/S	1/WT	0.06	
285	F3674	15J2053	SRR23915418	2015	F	66	Surgical	No	East	421	CCN2	Non-clade 4	Other	Diploid	-	-	R	-	-	2	256/R	8/R	1/NWT	1/NWT	0.03/S	0.03/S	0.03/S	1/WT	0.06	
286	F3624	14GX058	SRR23915648	2014	M	26	Medical	Yes	South	421	CCN2	Non-clade 4	Other	Diploid	-	-	3,R	-	-	2	8/R	1/R	0.5/WT	1/NWT	0.015/S	0.015/S	0.015/S	1/WT	0.06	
287	F4162	14ZZ139	SRR23915360	2014	M	52	ICU	No	Central	1361	CCN2	Non-clade 4	Other	Diploid	-	-	1,3,R	-	-	2	256/R	0.5/I	0.5/WT	0.5/NWT	0.25/S	0.03/S	0.12/S	1/WT	0.06	
288	3663	21XH663	SRR38060784	2021	M	34	ICU	Yes	Central	149	CCN2	Non-clade 4	Other	Diploid	-	-	R	-	-	2	2/S	0.12/S	0.25/WT	0.12/WT	0.06/S	0.03/S	0.06/S	1/WT	≤0.06	Yes
289	3661	21XH661	SRR38060783	2021	M	53	Surgical	No	Central	522	CCN2	Non-clade 4	Other	Diploid	-	-	R	-	-	2	4/SDD	0.12/S	0.25/WT	0.25/NWT	0.03/S	0.03/S	0.03/S	1/WT	≤0.06	Yes
290	L3724	23XH874	SRR38060829	2023	M	56	Surgical	No	Central	522	CCN2	Non-clade 4	Other	Diploid	-	-	R	-	-	2	4/SDD	0.12/S	0.25/WT	0.25/NWT	0.12/S	0.03/S	0.12/S	1/WT	≤0.06	Yes
291	A582	22GZ1661	SRR38060828	2022	M	38	Surgical	No	South	522	CCN2	Non-clade 4	Other	Diploid	-	-	1,2,3,5,R	-	-	2	2/S	0.25/I	0.25/WT	0.12/WT	4/R	2/R	0.25/S	1/WT	0.12	
292	3015	21SJ685	SRR38060827	2021	M	33	Medical	Yes	Northeast	337	CCN2	Non-clade 4	Other	Diploid	-	-	5,6,R	-	-	2	2/S	0.12/S	0.25/WT	0.25/NWT	0.06/S	0.03/S	0.03/S	2/WT	≤0.06	Yes
293	F9508	10HX058	SRR23915243	2010	F	59	Medical	Yes	Southwest	1361	CCN2	Non-clade 4	Other	Diploid	-	-	R	-	-	2	1/S	0.06/S	0.25/WT	0.12/WT	0.03/S	0.015/S	0.015/S	1/WT	≤0.06	
294	F3430	15TJ707	SRR23915349	2015	F	41	Surgical	No	Central	1361	CCN2	Non-clade 4	Other	Diploid	-	-	5,6,R	-	-	2	2/S	0.12/S	0.12/WT	0.12/WT	0.03/S	0.03/S	0.03/S	1/WT	0.25	
295	F4120	15PU1031	SRR23915564	2015	M	51	Other wards	No	North	1361	CCN2	Non-clade 4	Other	Diploid	-	-	2,R	-	-	2	2/S	0.12/S	0.25/WT	0.25/WT	0.03/S	0.03/S	0.03/S	1/WT	0.06	
296	F3890	15FJ420	SRR23915375	2015	M	80	ICU	No	East	1361	CCN2	Non-clade 4	Other	Diploid	-	chr 4-pos 1-2×	1,4,R	-	-	2	2/S	0.25/I	0.25/WT	0.25/WT	0.03/S	0.03/S	0.12/S	0.5/WT	0.06	
297	F3308	13XJ048	SRR23915406	2013	F	54	Medical	Yes	Northwest	1361	CCN2	Non-clade 4	Other	Diploid	-	-	1,R	-	-	2	2/S	0.12/S	0.25/WT	0.25/WT	0.03/S	0.015/S	0.03/S	1/WT	0.06	
298	F4030	15NX138	SRR23915589	2015	M	49	Medical	Yes	Northwest	421	CCN2	Non-clade 4	Other	Diploid	-	chr 4-pos 1-1.5×	R	-	-	2	1/S	0.12/S	0.25/WT	0.25/WT	0.03/S	0.03/S	0.03/S	1/WT	0.06	
299	F3620	14CQ080	SRR23915650	2014	F	45	ICU	Yes	Southwest	421	CCN2	Non-clade 4	Other	Diploid	-	-	2,R	-	-	2	1/S	0.12/S	0.12/WT	0.12/WT	0.06/S	0.015/S	0.03/S	0.5/WT	0.06	
300	F3518	15GX070	SRR23915674	2015	M	63	Medical	Yes	South	10037	CCN2	Non-clade 4	Other	Diploid	-	-	2,5,R	-	-	2	2/S	0.25/I	0.25/WT	0.25/WT	0.03/S	0.03/S	0.03/S	0.5/WT	0.06	
301	F4092	15HX888	SRR23915579	2015	M	56	Surgical	No	Southwest	1361	CCN2	Non-clade 4	Other	Diploid	-	chr 3-pos 1-2×	3,5,6,R	-	-	2	16/R	1/R	0.5/WT	0.5/NWT	0.06/S	0.06/S	0.12/S	1/WT	0.12	
302	F3612	14SJ123	SRR23915509	2014	M	28	ICU	No	Northeast	421	CCN2	Non-clade 4	Other	Diploid	-	-	4,R	-	-	2	1/S	0.12/S	0.25/WT	0.25/WT	0.03/S	0.015/S	0.03/S	1/WT	0.06	
303	F4146	11XY038	SRR23915369	2011	F	28	Surgical	No	Central	421	CCN2	Non-clade 4	Other	Diploid	-	-	6,R	-	-	2	2/S	0.12/S	0.25/WT	0.25/WT	0.03/S	0.015/S	0.015/S	1/WT	0.06	
304	F4144	11XY037	SRR23915370	2011	F	69	Medical	Yes	Central	421	CCN2	Non-clade 4	Other	Diploid	-	chr 4-pos 1-4×	6,R	-	-	2	2/S	0.12/S	0.25/WT	0.25/WT	0.03/S	0.015/S	0.015/S	1/WT	0.06	
305	F4148	11XY053	SRR23915368	2011	M	56	Surgical	Yes	Central	421	CCN2	Non-clade 4	Other	Diploid	-	-	6,R	-	-	2	2/S	0.25/I	0.25/WT	0.25/WT	0.03/S	0.015/S	0.015/S	1/WT	0.06	
306	F3386	16GS043	SRR23915552	2016	M	62	Surgical	Yes	Northwest	421	CCN2	Non-clade 4	Other	Diploid	-	-	6,R	-	-	2	2/S	0.12/S	0.5/WT	0.5/NWT	0.03/S	0.03/S	0.12/S	1/WT	0.06	
307	4952	21GZ1384	SRR38060826	2021	M	57	Surgical	No	South	1361	CCN2	Non-clade 4	Other	Diploid	-	-	1,R	-	-	2	2/S	0.06/S	0.12/WT	0.06/WT	0.06/S	0.03/S	0.03/S	0.5/WT	≤0.06	Yes
308	4974	21GZ1406	SRR38060825	2021	M	48	Other wards	No	South	522	CCN2	Non-clade 4	Other	Diploid	-	-	3,6,R	-	-	2	2/S	0.06/S	0.12/WT	0.12/WT	0.06/S	0.03/S	0.03/S	0.5/WT	≤0.06	Yes
309	4906	21GZ1338	SRR38060824	2021	M	63	Surgical	No	South	12034	CCN2	Non-clade 4	Other	Diploid	-	-	1,4,R	-	-	2	1/S	0.12/S	0.12/WT	0.12/WT	0.03/S	0.03/S	≤0.015/S	1/WT	≤0.06	Yes
310	5005	21GZ1437	SRR38060823	2021	F	50	Surgical	No	South	1313	CCN60	Non-clade 4	Other	Diploid	-	-	1	Y132F,S154F	Heterozygous	2	64/R	4/R	0.5/WT	0.5/NWT	0.12/S	0.03/S	0.25/S	1/WT	≤0.06	Yes
311	F3466	16DP094	SRR23915333	2016	M	62	Medical	Yes	Southwest	10031	CCN112	Non-clade 4	Other	Triploid	-	-	R	-	-	2	1/S	0.06/S	0.25/WT	0.12/WT	0.12/S	0.06/S	0.12/S	0.5/WT	0.06	
312	F3404	14GX029	SRR23915544	2014	M	66	ICU	Yes	South	10020	CCN113	Non-clade 4	Other	Diploid	-	-	R	-	-	2	1/S	0.12/S	0.25/WT	0.25/WT	0.06/S	0.03/S	0.12/S	1/WT	0.12	
313	F4058	15GH492	SRR23915271	2015	F	30	ICU	Yes	North	10065	CCN26	Non-clade 4	Other	Diploid	-	-	1,R	-	-	2	1/S	0.06/S	0.06/WT	0.06/WT	0.03/S	0.03/S	0.06/S	0.5/WT	0.06	
314	F3816	15PU999	SRR23915622	2015	M	48	Surgical	No	North	10065	CCN26	Non-clade 4	Other	Diploid	-	chr 4-pos 1-1.5×	1,6,R	-	-	2	2/S	0.5/I	0.5/WT	0.5/NWT	0.03/S	0.03/S	0.03/S	2/WT	0.06	
315	F3992	15HZ032	SRR23915279	2015	M	0	ICU	Yes	Central	10085	CCN114	Non-clade 4	Other	Diploid	-	-	2,R	-	-	2	2/S	0.12/S	0.25/WT	0.25/WT	0.06/S	0.03/S	0.12/S	2/WT	0.12	
316	F3652	14SJ104	SRR23915632	2014	M	39	Surgical	Yes	Northeast	10051	CCN10	Non-clade 4	Other	Diploid	-	-	R	-	-	2	1/S	0.06/S	0.12/WT	0.12/WT	0.03/S	0.06/S	0.25/S	0.5/WT	0.12	
317	F9512	11HX195	SRR23915239	2011	M	29	Medical	Yes	Southwest	10051	CCN10	Non-clade 4	Other	Diploid	-	-	1,R	-	-	2	1/S	0.06/S	0.12/WT	0.12/WT	0.06/S	0.03/S	0.12/S	1/WT	0.06	
318	F3942	14AH065	SRR23915466	2014	M	65	ICU	Yes	East	10081	CCN10	Non-clade 4	Other	Diploid	-	-	R	-	-	2	1/S	0.12/S	0.25/WT	0.25/WT	0.06/S	0.06/S	0.5/I	1/WT	0.12	
319	F3878	16XY279	SRR23915382	2016	F	19	Outpatient/Emergency	Yes	Central	10051	CCN10	Non-clade 4	Other	Diploid	-	-	1,R	-	-	2	2/S	0.12/S	0.25/WT	0.25/WT	0.12/S	0.06/S	0.12/S	0.5/WT	0.06	
320	F4056	15GH485	SRR23915272	2015	M	81	Medical	No	North	10051	CCN10	Non-clade 4	Other	Diploid	-	-	R	-	-	2	1/S	0.03/S	0.06/WT	0.06/WT	0.03/S	0.03/S	0.03/S	0.5/WT	0.06	
321	F3872	15HX847	SRR23915384	2015	M	42	Outpatient/Emergency	No	Southwest	10051	CCN10	Non-clade 4	Other	Diploid	-	-	1,R	-	-	2	1/S	0.06/S	0.12/WT	0.12/WT	0.25/S	0.06/S	0.25/S	1/WT	0.12	
322	F3882	16TJ823	SRR23915380	2016	M	52	Medical	Yes	Central	1269	CCN92	Non-clade 4	Other	Diploid	-	chr 4-pos 1-4×	1,4,5,R	-	-	2	1/S	0.06/S	0.12/WT	0.12/WT	0.06/S	0.03/S	0.06/S	1/WT	0.06	
323	F3936	15JS513	SRR23915470	2015	M	36	Medical	Yes	East	10079	CCN93	Non-clade 4	Other	Diploid	-	-	R	-	-	2	0.5/S	0.015/S	0.06/WT	0.03/WT	0.06/S	0.03/S	0.06/S	0.5/WT	0.12	
324	F9481	13BD278	SRR23915455	2013	M	64	Outpatient/Emergency	No	North	10090	CCN25	Non-clade 4	Other	Diploid	-	-	1,R	-	-	2	1/S	0.12/S	0.12/WT	0.12/WT	0.06/S	0.03/S	0.12/S	1/WT	≤0.06	
325	F4050	15GH483	SRR23915275	2015	F	36	Surgical	No	North	10090	CCN25	Non-clade 4	Other	Diploid	-	-	1,R	-	-	2	1/S	0.06/S	0.12/WT	0.12/WT	0.03/S	0.03/S	0.03/S	0.5/WT	0.06	
326	F3916	16PU1111	SRR23915477	2016	M	73	Surgical	No	North	10077	CCN24	Non-clade 4	Other	Diploid	-	-	1,6,R	-	-	2	2/S	0.12/S	0.25/WT	0.25/WT	0.03/S	0.03/S	0.03/S	1/WT	0.12	
327	F4114	15PU1020	SRR23915567	2015	F	20	ICU	No	North	511	CCN25	Non-clade 4	Other	Diploid	-	-	R	-	-	2	2/S	0.12/S	0.12/WT	0.25/WT	0.06/S	0.03/S	0.06/S	2/WT	0.06	
328	F3814	15PU1044	SRR23915623	2015	F	20	ICU	No	North	511	CCN25	Non-clade 4	Other	Diploid	-	-	R	-	-	2	2/S	0.12/S	0.12/WT	0.12/WT	0.03/S	0.03/S	0.06/S	1/WT	0.06	
329	F3638	16HS341	SRR23915640	2016	M	29	Surgical	No	East	10049	CCN94	Non-clade 4	Other	Diploid	-	-	1,4,R	-	-	2	32/R	2/R	0.25/WT	0.12/WT	0.12/S	0.03/S	0.25/S	0.25/WT	0.06	
330	F3498	15FJ435	SRR23915428	2015	F	64	Medical	No	East	10017	CCN95	Non-clade 4	Other	Diploid	-	-	-	-	-	2	2/S	0.25/I	0.25/WT	0.25/WT	0.03/S	0.03/S	0.03/S	0.5/WT	0.06	
331	F3384	16GY012	SRR23915553	2016	M	64	ICU	No	Southwest	10017	CCN95	Non-clade 4	Other	Diploid	-	-	R	-	-	2	2/S	0.12/S	0.25/WT	0.25/WT	0.03/S	0.03/S	0.015/S	0.5/WT	0.06	
332	F3438	15GF028	SRR23915346	2015	M	43	ICU	Yes	South	10028	CCN96	Non-clade 4	Other	Diploid	-	-	6	-	-	2	1/S	0.06/S	0.06/WT	0.06/WT	0.03/S	0.03/S	0.015/S	1/WT	0.06	
333	F3340	15GH476	SRR23915607	2015	M	39	Outpatient/Emergency	No	North	10010	CCN97	Non-clade 4	Other	Diploid	-	-	-	-	-	2	1/S	0.06/S	0.12/WT	0.12/WT	0.03/S	0.03/S	0.03/S	0.5/WT	0.06	
334	F3666	16GY022	SRR23915629	2016	F	61	ICU	Yes	Southwest	169	CCN3	Non-clade 4	Other	Diploid	-	-	2,6,R	-	-	2	2/S	0.12/S	0.5/WT	0.25/WT	0.03/S	0.06/S	0.12/S	1/WT	0.06	
335	F4160	13ZR020	SRR23915361	2013	M	56	Medical	No	Northeast	169	CCN3	Non-clade 4	Other	Diploid	-	-	1,2,4,6,R	-	-	2	4/SDD	0.5/I	0.5/WT	0.5/NWT	0.25/S	0.06/S	0.5/I	2/WT	0.12	
336	F3296	15X1061	SRR23915417	2015	F	35	ICU	Yes	Northwest	169	CCN3	Non-clade 4	Other	Diploid	-	-	2,6,R	-	-	2	0.25/S	0.008/S	0.06/WT	0.03/WT	0.06/S	0.015/S	0.06/S	1/WT	0.12	
337	F3338	15GH481	SRR23915618	2015	F	39	Medical	No	North	169	CCN3	Non-clade 4	Other	Diploid	-	-	2,6,R	-	-	2	1/S	0.06/S	0.12/WT	0.06/WT	0.06/S	0.03/S	0.12/S	1/WT	0.06	
338	F3636	14HB039	SRR23915641	2014	M	67	Medical	Yes	North	169	CCN3	Non-clade 4	Other	Diploid	-	-	2,6,R	-	-	2	1/S	0.06/S	0.06/WT	0.06/WT	0.03/S	0.03/S	0.015/S	1/WT	0.06	
339	F3724	15HB050	SRR23915502	2015	M	63	Medical	Yes	North	169	CCN3	Non-clade 4	Other	Diploid	-	chr 4-pos 1-1.5×	2,6,R	-	-	2	1/S	0.06/S	0.12/WT	0.06/WT	0.015/S	0.015/S	0.015/S	1/WT	0.06	
340	F3844	15A1052	SRR23915606	2015	M	63	Medical	No	Northwest	169	CCN3	Non-clade 4	Other	Diploid	-	-	2,6,R	-	-	2	1/S	0.12/S	0.25/WT	0.25/WT	0.03/S	0.03/S	0.12/S	1/WT	0.06	
341	F3978	14LR214	SRR23915287	2014	M	59	ICU	Yes	Northeast	169	CCN3	Non-clade 4	Other	Diploid	-	-	2,6,R	-	-	2	1/S	0.06/S	0.12/WT	0.12/WT	0.06/S	0.03/S	0.03/S	1/WT	0.06	
342	F9490	11H1164	SRR23915446	2011	M	37	Medical	Yes	Northeast	169	CCN3	Non-clade 4	Other	Diploid	-	-	2,6,R	-	-	2	1/S	0.06/S	0.25/WT	0.25/WT	0.03/S	0.015/S	0.015/S	0.5/WT	≤0.06	
343	F4046	15DL070	SRR23915581	2015	F	48	ICU	Yes	Northeast	169	CCN3	Non-clade 4	Other	Diploid	-	-	2,6,R	-	-	2	2/S	0.12/S	0.12/WT	0.25/WT	0.06/S	0.06/S	0.12/S	0.5/WT	0.06	
344	F3364	14XJ086	SRR23915254	2014	F	62	Medical	Yes	Northwest	169	CCN3	Non-clade 4	Other	Diploid	-	-	2,6,R	-	-	2	2/S	0.25/I	0.25/WT	0.5/NWT	0.03/S	0.03/S	0.03/S	2/WT	0.06	
345	F3586	14SS009	SRR23915521	2014	M	58	Medical	Yes	North	169	CCN3	Non-clade 4	Other	Diploid	-	-	2,5,6,R	-	-	2	1/S	0.12/S	0.25/WT	0.25/WT	0.06/S	0.03/S	0.06/S	1/WT	0.06	
346	F3836	15PU1000	SRR23915611	2015	F	15	Outpatient/Emergency	Yes	North	169	CCN3	Non-clade 4	Other	Diploid	-	-	2,6,R	-	-	2	16/R	0.5/I	1/NWT	2/NWT	0.03/S	0.03/S	0.015/S	1/WT	0.06	
347	4947	21GZ1379	SRR38060822	2021	M	40	ICU	No	South	169	CCN3	Non-clade 4	Other	Diploid	-	-	2,6,R	-	-	2	2/S	0.06/S	0.06/WT	0.06/WT	0.06/S	0.06/S	0.12/S	1/WT	≤0.06	Yes
348	F3822	15Z2146	SRR23915619	2015	M	75	Surgical	No	East	10067	CCN3	Non-clade 4	Other	Diploid	-	-	2,6,R	-	-	2	1/S	0.06/S	0.12/WT	0.12/WT	0.03/S	0.03/S	0.03/S	1/WT	0.06	
349	F3840	15A1033	SRR23915609	2015	M	40	ICU	No	Northwest	169	CCN3	Non-clade 4	Other	Diploid	-	chr 4-pos 1-2×	2,6,R	-	-	2	2/S	0.12/S	0.25/WT	0.25/WT	0.03/S	0.03/S	0.06/S	1/WT	0.06	
350	F3154	14H1409	SRR23915297	2014	M	20	Medical	Yes	Northeast	415	CCN3	Non-clade 4	Other	Diploid	-	-	2,3,6,R	-	-	2	0.25/S	0.008/S	0.015/WT	0.015/WT	0.015/S	0.015/S	0.015/S	1/WT	0.06	
351	F3954	11XY014	SRR23915301	2011	F	66	ICU	No	Central	415	CCN3	Non-clade 4	Other	Diploid	-	-	3,R	-	-	2	1/S	0.12/S	0.25/WT	0.25/WT	0.03/S	0.03/S	0.03/S	1/WT	0.06	
352	F3310	13XJ053	SRR23915506	2013	F	26	ICU	Yes	Northwest	169	CCN3	Non-clade 4	Other	Diploid	-	chr 4-pos 1-3.5×	1,2,3,4,6,R	-	-	2	2/S	0.12/S	0.25/WT	0.12/WT	0.03/S	0.03/S	0.06/S	1/WT	0.06	
353	F3704	15GH489	SRR23915402	2015	F	36	Surgical	Yes	North	169	CCN3	Non-clade 4	Other	Diploid	-	-	2,4,6,R	-	-	2	1/S	0.03/S	0.06/WT	0.06/WT	0.03/S	0.03/S	0.03/S	0.5/WT	0.06	
354	F4128	14SF023	SRR23915561	2014	F	20	Surgical	Yes	North	169	CCN3	Non-clade 4	Other	Diploid	-	-	2,6,R	-	-	2	2/S	0.12/S	0.12/WT	0.25/WT	0.03/S	0.03/S	0.12/S	1/WT	0.06	
355	F3698	15H1434	SRR23915405	2015	M	44	ICU	No	Northeast	174	CCN3	Non-clade 4	Other	Diploid	-	-	1,2,3,6,R	-	-	2	1/S	0.06/S	0.06/WT	0.12/WT	0.015/S	0.015/S	0.015/S	1/WT	0.06	
356	F9475	12BD183	SRR23915350	2012	M	80	ICU	No	North	10105	CCN3	Non-clade 4	Other	Diploid	-	-	2,6,R	-	-	2	2/S	0.06/S	0.12/WT	0.12/WT	0.03/S	0.03/S	0.015/S	1/WT	≤0.06	
357	F9523	12HX598	SRR23915554	2012	M	39	Medical	Yes	Southwest	169	CCN3	Non-clade 4	Other	Diploid	-	-	2,6,R	-	-	2	2/S	0.12/S	0.25/WT	0.25/WT	0.03/S	0.03/S	0.12/S	0.5/WT	≤0.06	
358	F3760	15Z2125	SRR23915322	2015	F	78	Outpatient/Emergency	Yes	East	169	CCN3	Non-clade 4	Other	Diploid	-	-	2,6,R	-	-	2	1/S	0.06/S	0.12/WT	0.12/WT	0.06/S	0.03/S	0.06/S	1/WT	0.06	
359	F3642	15H1469	SRR23915638	2015	M	0	ICU	Yes	Northeast	169	CCN3	Non-clade 4	Other	Diploid	-	-	2,6,R	-	-	2	2/S	0.12/S	0.25/WT	0.25/WT	0.03/S	0.06/S	0.03/S	1/WT	0.06	
360	F3644	15H1470	SRR23915637	2015	M	0	ICU	Yes	Northeast	169	CCN3	Non-clade 4	Other	Diploid	-	-	2,6,R	-	-	2	2/S	0.12/S	0.25/WT	0.25/WT	0.03/S	0.03/S	0.03/S	1/WT	0.06	
361	F3818	15Z2150	SRR23915621	2015	M	72	Surgical	Yes	East	1298	CCN3	Non-clade 4	Other	Diploid	-	-	2,5,6,R	-	-	2	2/S	0.12/S	0.25/WT	0.12/WT	0.03/S	0.06/S	0.12/S	1/WT	0.06	
362	F4032	15LZ100	SRR23915588	2015	F	41	ICU	Yes	Northwest	169	CCN3	Non-clade 4	Other	Diploid	-	-	2,6,R	-	-	2	1/S	0.06/S	0.12/WT	0.12/WT	0.03/S	0.03/S	0.06/S	1/WT	0.06	
363	F4064	16GH544	SRR23915268	2016	M	65	Surgical	No	North	169	CCN3	Non-clade 4	Other	Diploid	-	-	2,6,R	-	-	2	4/SDD	0.25/I	0.5/WT	0.5/NWT	0.12/S	0.03/S	0.03/S	0.5/WT	0.06	
364	F3734	15G1208	SRR23915497	2015	M	26	ICU	No	South	169	CCN3	Non-clade 4	Other	Diploid	-	-	2,6,R	-	-	2	1/S	0.12/S	0.25/WT	0.25/WT	0.03/S	0.03/S	0.03/S	1/WT	0.06	
365	F3556	15JS389	SRR23915654	2015	M	37	Medical	Yes	East	169	CCN3	Non-clade 4	Other	Diploid	-	-	2,6,R	-	-	2	1/S	0.06/S	0.12/WT	0.12/WT	0.06/S	0.03/S	0.06/S	1/WT	0.06	
366	Z4119	21ZR325	SRR38060821	2021	F	30	Surgical	No	Northeast	415	CCN3	Non-clade 4	Other	Diploid	-	-	2,4,6,R	-	-	2	2/S	0.12/S	0.12/WT	0.12/WT	0.03/S	0.06/S	0.12/S	0.5/WT	≤0.06	Yes
367	Z4117	21ZR323	SRR38060820	2021	F	30	Surgical	No	Northeast	415	CCN3	Non-clade 4	Other	Diploid	-	-	2,6,R	-	-	2	2/S	0.12/S	0.12/WT	0.12/WT	0.06/S	0.06/S	0.25/S	1/WT	≤0.06	Yes
368	Z5327	20T1376	SRR38060818	2020	M	68	Surgical	No	North	1376	CCN17	Non-clade 4	Other	Diploid	-	-	2	-	-	2	4/SDD	0.25/I	0.25/WT	0.25/NWT	0.06/S	0.06/S	0.12/S	1/WT	0.12	Yes
369	Z5312	20T1361	SRR38060817	2020	M	48	Surgical	No	North	1376	CCN17	Non-clade 4	Other	Diploid	-	-	2	-	-	2	2/S	0.12/S	0.25/WT	0.25/NWT	0.06/S	0.06/S	0.12/S	2/WT	0.12	Yes
370	2683	21PU1840	SRR38060816	2021	M	65	Outpatient/Emergency	No	North	12032	CCN17	Non-clade 4	Other	Diploid	-	-	2,3	-	-	2	1/S	0.06/S	0.12/WT	0.12/WT	0.06/S	0.06/S	0.12/S	1/WT	≤0.06	Yes
371	4868	21GZ1300	SRR38060815	2021	M	54	Surgical	No	South	1376	CCN17	Non-clade 4	Other	Diploid	-	-	2	-	-	2	2/S	0.12/S	0.12/WT	0.12/WT	0.06/S	0.06/S	0.12/S	0.5/WT	≤0.06	Yes
372	4866	21GZ1298	SRR38060814	2021	M	47	Surgical	No	South	1376	CCN17	Non-clade 4	Other	Diploid	-	-	2	-	-	2	2/S	0.12/S	0.12/WT	0.12/WT	0.06/S	0.06/S	0.12/S	1/WT	≤0.06	Yes
373	5047	21GZ1479	SRR38060813	2021	M	51	ICU	No	South	12033	CCN61	Non-clade 4	Other	Diploid	chr 3-1.5×	-	2,3,4,5,6,R	-	-	2	0.5/S	0.03/S	0.06/WT	0.06/WT	0.03/S	0.06/S	0.03/S	0.5/WT	≤0.06	Yes
374	4982	21GZ1414	SRR38060812	2021	M	0	ICU	No	South	1636	CCN56	Non-clade 4	Other	Diploid	-	-	1,2,3,4,5,R	-	-	2	2/S	0.12/S	0.12/WT	0.12/WT	0.03/S	0.03/S	≤0.015/S	0.5/WT	≤0.06	Yes
375	4971	21GZ1403	SRR38060811	2021	F	61	Surgical	No	South	10083	CCN10	Non-clade 4	Other	Diploid	-	-	2,5,R	-	-	2	2/S	0.12/S	0.12/WT	0.06/WT	0.03/S	0.03/S	0.12/S	1/WT	0.5	Yes
376	4931	21GZ1363	SRR38060810	2021	M	84	Surgical	No	South	12031	CCN53	Non-clade 4	Other	Octoploid	-	-	-	-	-	2	2/S	0.12/S	0.12/WT	0.12/WT	0.06/S	0.06/S	0.25/S	0.5/WT	≤0.06	Yes
377	3695	21XH695	SRR38060809	2021	M	60	Surgical	Yes	Central	1368	CCN20	Non-clade 4	Other	Diploid	-	-	4,R	-	-	2	4/SDD	0.12/S	0.12/WT	0.25/NWT	0.12/S	0.06/S	0.12/S	1/WT	≤0.06	Yes
378	4822	21GZ1254	SRR38060807	2021	M	31	ICU	Yes	South	12016	CCN52	Non-clade 4	Other	Diploid	-	-	1,6,R	-	-	2	0.5/S	0.015/S	≤0.015/WT	0.015/WT	0.03/S	0.015/S	≤0.015/S	0.5/WT	≤0.06	Yes
379	F4100	15JD067	SRR23915575	2015	F	50	Surgical	No	Northeast	10094	CCN5	Non-clade 4	Other	Diploid	-	-	1,2,3,6,R	-	-	2	1/S	0.06/S	0.12/WT	0.12/WT	0.25/S	0.03/S	0.25/S	1/WT	0.12	
380	F4054	16GH564	SRR23915273	2016	M	63	Surgical	No	North	134	CCN5	Non-clade 4	Other	Diploid	-	-	1,2,3,6,R	-	-	2	1/S	0.12/S	0.12/WT	0.12/WT	0.06/S	0.03/S	0.12/S	0.5/WT	0.12	
381	F3626	13J3019	SRR23915647	2013	M	84	Surgical	No	East	10048	CCN5	Non-clade 4	Other	Diploid	-	-	1,2,3,4,5,6,R	-	-	2	4/SDD	0.25/I	0.25/WT	0.25/WT	0.03/S	0.03/S	0.03/S	0.5/WT	0.06	
382	F3640	16HS348	SRR23915639	2016	M	74	Surgical	No	East	1344	CCN5	Non-clade 4	Other	Diploid	-	-	1,3,4,5,6,R	-	-	2	1/S	0.06/S	0.12/WT	0.12/WT	0.25/S	0.06/S	0.25/S	0.25/WT	0.06	
383	Z5604	21H4679	SRR38060839	2021	M	48	Medical	No	Northeast	12019	CCN43	Non-clade 4	Other	Diploid	-	-	1,2,3,4,6,R	-	-	2	1/S	0.03/S	0.12/WT	0.03/WT	0.06/S	0.06/S	0.12/S	1/WT	≤0.06	Yes
384	Z5599	21H4674	SRR38060838	2021	M	48	Medical	No	Northeast	12019	CCN43	Non-clade 4	Other	Diploid	-	-	1,2,3,4,6,R	-	-	2	2/S	0.06/S	0.12/WT	0.03/WT	0.12/S	0.06/S	0.12/S	1/WT	≤0.06	Yes
385	F9479	13BD268	SRR23915457	2013	M	61	Surgical	No	North	134	CCN5	Non-clade 4	Other	Diploid	-	-	1,2,3,6,R	-	-	2	8/R	0.5/I	0.25/WT	0.25/WT	0.03/S	0.03/S	0.06/S	1/WT	0.25	
386	F3614	14JD054	SRR23915652	2014	M	66	Medical	No	Northeast	134	CCN5	Non-clade 4	Other	Diploid	-	-	1,3,4,6,R	-	-	2	1/S	0.12/S	0.25/WT	0.25/WT	0.03/S	0.06/S	0.12/S	1/WT	0.5	
387	F3776	16LR314	SRR23915314	2016	F	81	Surgical	No	Northeast	10061	CCN5	Non-clade 4	Other	Diploid	-	-	1,3,4,6,R	-	-	2	1/S	0.06/S	0.12/WT	0.12/WT	0.06/S	0.06/S	0.12/S	1/WT	0.12	
388	F3802	14DZ013	SRR23915628	2014	M	91	ICU	Yes	Northeast	682	CCN5	Non-clade 4	Other	Diploid	-	-	2,3,4,5,6,R	-	-	2	2/S	0.12/S	0.25/WT	0.25/WT	0.25/S	0.03/S	0.25/S	0.5/WT	0.06	
389	F9494	12H1213	SRR23915441	2012	M	42	Surgical	Yes	Northeast	10108	CCN62	Non-clade 4	Other	Diploid	-	-	1,2,3,4,6,R	-	-	2	1/S	0.06/S	0.12/WT	0.06/WT	0.12/S	0.03/S	0.25/S	1/WT	0.12	
390	F3152	14H1405	SRR23915495	2014	M	73	Medical	Yes	Northeast	10004	CCN63	Non-clade 4	Other	Diploid	-	-	1,2,3,4,6,R	-	-	2	0.25/S	0.008/S	0.015/WT	0.015/WT	0.015/S	0.008/S	0.015/S	1/WT	0.12	
391	F3968	15K1071	SRR23915292	2015	F	53	Medical	No	Southwest	1171	CCN5	Non-clade 4	Other	Diploid	-	-	1,2,3,4,5,6,R	-	-	2	0.5/S	0.03/S	0.06/WT	0.03/WT	0.06/S	0.03/S	0.12/S	0.25/WT	0.06	
392	4846	21GZ1278	SRR38060837	2021	F	56	Other wards	No	South	1171	CCN5	Non-clade 4	Other	Diploid	-	-	2,3,4,5,6,R	-	-	2	1/S	0.03/S	≤0.015/WT	0.015/WT	0.06/S	0.03/S	0.12/S	0.5/WT	≤0.06	Yes
393	F3436	15GF029	SRR23915347	2015	F	74	Medical	Yes	South	10027	CCN68	Non-clade 4	Other	Diploid	-	-	R	-	-	2	0.5/S	0.03/S	0.06/WT	0.06/WT	0.015/S	0.03/S	0.015/S	0.5/WT	0.06	
394	F3598	15GF001	SRR23915515	2015	M	52	ICU	Yes	South	10045	CCN69	Non-clade 4	Other	Diploid	-	-	1,3,6,R	-	-	2	0.12/S	0.03/S	0.06/WT	0.06/WT	0.015/S	0.015/S	0.015/S	0.5/WT	0.06	
395	F3980	15DP066	SRR23915286	2015	F	60	Surgical	Yes	Southwest	1000	CCN70	4	Other	Diploid	-	-	1,3,4,6,R	-	-	2	1/S	0.06/S	0.25/WT	0.12/WT	0.03/S	0.06/S	0.12/S	0.5/WT	0.06	
396	F3650	14SF039	SRR23915633	2014	M	59	ICU	No	North	10050	CCN71	Non-clade 4	Other	Diploid	-	-	6,R	-	-	2	1/S	0.06/S	0.12/WT	0.06/WT	0.03/S	0.06/S	0.25/S	1/WT	0.06	
397	F3420	15H4189	SRR23915538	2015	M	73	Medical	No	Northeast	10024	CCN72	Non-clade 4	Other	Diploid	-	-	R	-	-	2	1/S	0.12/S	0.12/WT	0.12/WT	0.015/S	0.03/S	0.03/S	1/WT	0.06	
398	F3884	16TJ822	SRR23915379	2016	F	51	Other wards	Yes	Central	10073	CCN73	Non-clade 4	Other	Diploid	-	-	1,2,3,4,5,6,R	-	-	2	1/S	0.12/S	0.25/WT	0.25/WT	0.03/S	0.03/S	0.03/S	1/WT	0.12	
399	F3780	15TJ676	SRR23915311	2015	M	50	Surgical	Yes	Central	991	CCN75	Non-clade 4	Other	Diploid	-	-	R	-	-	2	2/S	0.5/I	0.5/WT	0.5/NWT	0.03/S	0.06/S	0.12/S	1/WT	0.12	
400	F4096	12J3001	SRR23915577	2012	M	29	Other wards	No	East	991	CCN75	Non-clade 4	Other	Diploid	-	-	R	-	-	2	1/S	0.06/S	0.12/WT	0.12/WT	0.03/S	0.03/S	0.03/S	1/WT	0.06	
401	F3952	13ZR019	SRR23915461	2013	M	49	Medical	Yes	Northeast	991	CCN75	Non-clade 4	Other	Diploid	-	-	R	-	-	2	1/S	0.03/S	0.25/WT	0.12/WT	0.03/S	0.03/S	0.015/S	1/WT	0.06	
402	F4000	15LZ098	SRR23915602	2015	F	59	ICU	No	Northwest	991	CCN75	Non-clade 4	Other	Diploid	-	-	R	-	-	2	1/S	0.06/S	0.06/WT	0.06/WT	0.03/S	0.03/S	0.06/S	1/WT	0.06	
403	F4060	15GH493	SRR23915270	2015	M	69	Surgical	No	North	10007	CCN74	Non-clade 4	Other	Diploid	-	-	2,3,R	-	-	2	1/S	0.03/S	0.12/WT	0.06/WT	0.06/S	0.03/S	0.015/S	0.5/WT	0.06	
404	F4118	15PU1027	SRR23915565	2015	M	77	ICU	No	North	10007	CCN74	Non-clade 4	Other	Diploid	-	-	2,3,R	-	-	2	1/S	0.12/S	0.25/WT	0.25/WT	0.03/S	0.03/S	0.03/S	2/WT	0.06	
405	F3200	15K1054	SRR23915655	2015	M	67	Medical	Yes	Southwest	10007	CCN74	Non-clade 4	Other	Diploid	-	-	2,3,R	-	-	2	0.25/S	0.008/S	0.03/WT	0.03/WT	0.03/S	0.015/S	0.06/S	0.25/WT	64	
406	Z375	20CQ484	SRR38060836	2020	F	75	ICU	No	Southwest	12022	CCN36	Non-clade 4	Other	Diploid	-	-	1,2,5,6,R	-	-	2	0.5/S	0.03/S	0.06/WT	0.06/WT	0.03/S	0.03/S	0.12/S	0.5/WT	≤0.06	Yes
407	4966	21GZ1398	SRR38060835	2021	M	59	ICU	No	South	12011	CCN18	Non-clade 4	Other	Diploid	-	-	2,R	-	-	2	2/S	0.06/S	0.12/WT	0.12/WT	0.03/S	0.03/S	0.12/S	0.5/WT	≤0.06	Yes
408	3021	21SJ691	SRR38060834	2021	F	9	Medical	Yes	Northeast	1907	CCN46	Non-clade 4	Other	Diploid	-	-	1,2	-	-	2	2/S	0.25/I	0.25/WT	0.5/NWT	0.06/S	0.06/S	0.25/S	1/WT	≤0.06	Yes
409	4972	21GZ1404	SRR38060833	2021	M	61	Surgical	No	South	12025	CCN55	Non-clade 4	Other	Diploid	-	-	R	-	-	2	2/S	0.12/S	0.12/WT	0.12/WT	0.06/S	0.06/S	0.06/S	0.5/WT	≤0.06	Yes
410	Z2730	20QQ031	SRR38060832	2020	F	74	Surgical	No	East	399	CCN29	Non-clade 4	Other	Diploid	-	-	3,R	-	-	2	2/S	0.12/S	0.25/WT	0.25/WT	0.03/S	0.06/S	0.12/S	0.5/WT	≤0.06	Yes
411	4844	21GZ1276	SRR38060831	2021	F	64	ICU	No	South	399	CCN29	Non-clade 4	Other	Diploid	-	-	1,3,4,R	-	-	2	1/S	0.12/S	0.12/WT	0.12/WT	0.06/S	0.03/S	0.12/S	1/WT	≤0.06	Yes
412	L5713	22X1180	SRR38060863	2022	F	10	ICU	Yes	Northwest	1557	CCN44	Non-clade 4	Other	Diploid	-	-	R	-	-	2	4/SDD	0.25/I	0.25/WT	0.25/NWT	0.12/S	0.06/S	0.25/S	2/WT	0.12	Yes
413	F3722	15HB049	SRR23915503	2015	F	37	Medical	Yes	North	10005	CCN76	Non-clade 4	Other	Diploid	-	-	2,3,R	-	-	2	0.5/S	0.03/S	0.06/WT	0.06/WT	0.06/S	0.03/S	0.06/S	1/WT	0.06	
414	F3160	14H1414	SRR23915354	2014	F	56	Medical	Yes	Northeast	10005	CCN76	Non-clade 4	Other	Diploid	-	-	2,6	-	-	2	1/S	0.06/S	0.06/WT	0.06/WT	0.06/S	0.03/S	0.06/S	1/WT	0.06	
415	F3618	14CQ077	SRR23915651	2014	F	45	ICU	Yes	Southwest	110	CCN67	Non-clade 4	Other	Diploid	-	-	1,2,3,R	-	-	2	2/S	0.06/S	0.06/WT	0.06/WT	0.06/S	0.03/S	0.12/S	0.5/WT	0.06	
416	Z5310	20T1359	SRR38060862	2020	M	56	Surgical	No	North	12013	CCN40	Non-clade 4	Other	Diploid	-	-	-	-	-	2	2/S	0.12/S	0.12/WT	0.12/WT	0.03/S	0.06/S	≤0.015/S	2/WT	0.12	Yes
417	Z5347	20T1396	SRR38060861	2020	M	56	Surgical	No	North	12013	CCN40	Non-clade 4	Other	Diploid	-	-	-	-	-	2	2/S	0.12/S	0.12/WT	0.12/WT	0.03/S	0.06/S	0.03/S	1/WT	0.12	Yes
418	Z5314	20T1363	SRR38060860	2020	M	56	Surgical	No	North	12013	CCN40	Non-clade 4	Other	Diploid	-	-	-	-	-	2	2/S	0.12/S	0.12/WT	0.25/NWT	0.03/S	0.06/S	0.03/S	1/WT	0.12	Yes
419	3715	21XH715	SRR38060859	2021	F	53	Medical	Yes	Central	1138	CCN42	Non-clade 4	Other	Diploid	-	-	4,5,6,R	-	-	2	2/S	0.12/S	0.12/WT	0.06/WT	0.06/S	0.06/S	0.06/S	1/WT	≤0.06	Yes
420	Z2405	21NX666	SRR38060858	2021	F	52	Surgical	No	Northwest	1138	CCN42	Non-clade 4	Other	Diploid	-	-	5,6,R	-	-	2	1/S	0.06/S	0.12/WT	0.12/WT	0.03/S	0.06/S	0.06/S	2/WT	≤0.06	Yes
421	Z2326	21NX587	SRR38060857	2021	F	52	Surgical	No	Northwest	1138	CCN42	Non-clade 4	Other	Diploid	-	-	5,6,R	-	-	2	1/S	0.06/S	0.12/WT	0.12/WT	0.03/S	0.06/S	0.06/S	0.5/WT	≤0.06	Yes

422	3128	21SJ798	SRR38060856	2021	M	44	Surgical	No	Northeast	489	CCN49	Non-clade 4	Other	Diploid	-	-	1,5,6,R	-	-	2	>256/R	1/R	1/NWT	0.5/NWT	0.25/S	0.06/S	0.25/S	2/WT	0.12	Yes
423	F4154	15ZD403	SRR23915364	2015	M	82	Surgical	Yes	East	10100	CCN64	Non-clade 4	Other	Diploid	-	-	2,R	-	-	2	1/S	0.12/S	0.12/WT	0.25/WT	0.03/S	0.03/S	0.03/S	0.5/WT	0.06	
424	F3490	14G1188	SRR23915431	2014	M	38	Medical	Yes	South	10032	CCN65	Non-clade 4	Other	Diploid	-	-	1,2,R	-	-	2	1/S	0.06/S	0.12/WT	0.12/WT	0.03/S	0.03/S	0.06/S	0.5/WT	0.06	
425	F3524	15FJ427	SRR23915672	2015	F	27	Medical	Yes	East	10038	CCN20	Non-clade 4	Other	Diploid	-	-	5,R	-	-	2	2/S	0.12/S	0.25/WT	0.12/WT	0.06/S	0.06/S	0.12/S	1/WT	0.06	
426	F4074	14AH091	SRR23915262	2014	M	62	Surgical	No	East	192	CCN66	Non-clade 4	Other	Diploid	-	-	1,R	-	-	2	1/S	0.06/S	0.12/WT	0.12/WT	0.06/S	0.03/S	0.03/S	1/WT	0.06	
427	F3610	14SJ120	SRR23915510	2014	M	68	ICU	No	Northeast	10047	CCN98	Non-clade 4	Other	Diploid	-	-	1,2,4,R	-	-	2	1/S	0.015/S	0.06/WT	0.06/WT	0.5/I	1/R	0.5/I	0.25/WT	0.06	
428	F3388	16GF093	SRR23915551	2016	F	61	Medical	Yes	South	10018	CCN99	Non-clade 4	Other	Triploid	-	-	R	-	-	2	1/S	0.06/S	0.25/WT	0.06/WT	0.06/S	0.03/S	0.12/S	0.5/WT	0.12	
429	F3910	15CQ230	SRR23915480	2015	M	30	Medical	Yes	Southwest	10076	CCN100	Non-clade 4	Other	Diploid	-	-	R	-	-	2	2/S	0.12/S	0.25/WT	0.5/NWT	0.12/S	0.06/S	0.12/S	0.5/WT	0.06	
430	F3378	15FJ461	SRR23915442	2015	M	35	Medical	Yes	East	10016	CCN101	Non-clade 4	Other	Triploid	-	-	R	-	-	2	2/S	0.12/S	0.25/WT	0.25/WT	0.06/S	0.03/S	0.12/S	0.5/WT	0.12	
431	F3934	15FJ458	SRR23915471	2015	M	35	Medical	Yes	East	10016	CCN101	Non-clade 4	Other	Triploid	-	-	R	-	-	2	1/S	0.12/S	0.25/WT	0.25/WT	0.03/S	0.03/S	0.12/S	0.5/WT	0.25	
432	F3846	15FJ459	SRR23915605	2015	M	35	Medical	Yes	East	10016	CCN101	Non-clade 4	Other	Triploid	-	-	R	-	-	2	2/S	0.25/I	0.25/WT	0.25/WT	0.06/S	0.06/S	0.12/S	0.5/WT	0.12	
433	F3748	15A1027	SRR23915488	2015	F	31	Medical	No	Northwest	10057	CCN102	Non-clade 4	Other	Diploid	-	chr 4-pos 1-1.5×	3,R	-	-	2	2/S	0.12/S	0.25/WT	0.25/WT	0.03/S	0.03/S	0.06/S	1/WT	0.12	
434	F3702	15GH487	SRR23915403	2015	M	26	Medical	Yes	North	10016	CCN101	Non-clade 4	Other	Triploid	-	chr 4-pos 1-3×	R	-	-	2	1/S	0.06/S	0.12/WT	0.06/WT	0.03/S	0.03/S	0.06/S	0.5/WT	0.06	
435	F3864	16DL080	SRR23915388	2016	M	79	ICU	No	Northeast	730	CCN12	Non-clade 4	Other	Diploid	-	-	R	-	-	2	1/S	0.06/S	0.12/WT	0.12/WT	0.06/S	0.06/S	0.12/S	0.5/WT	0.12	
436	F3930	16SS023	SRR23915473	2016	M	74	Medical	No	North	730	CCN12	Non-clade 4	Other	Diploid	-	-	R	-	-	2	2/S	0.25/I	0.25/WT	0.25/WT	0.06/S	0.03/S	0.12/S	0.5/WT	0.12	
437	F4088	16HS472	SRR23915255	2016	F	18	Surgical	No	East	730	CCN12	Non-clade 4	Other	Diploid	-	-	2,3,R	-	-	2	1/S	0.06/S	0.12/WT	0.12/WT	0.25/S	0.06/S	0.25/S	1/WT	0.12	
438	F4086	16HS467	SRR23915256	2016	F	18	Surgical	No	East	10093	CCN12	Non-clade 4	Other	Diploid	-	-	2,3,R	-	-	2	1/S	0.06/S	0.12/WT	0.12/WT	0.25/S	0.06/S	0.25/S	1/WT	0.12	
439	F3564	15NX182	SRR23915530	2015	M	51	ICU	No	Northwest	977	CCN12	Non-clade 4	Other	Diploid	-	-	3	-	-	2	1/S	0.06/S	0.12/WT	0.12/WT	0.03/S	0.06/S	0.25/S	1/WT	0.12	
440	F3486	15TJ683	SRR23915434	2015	M	19	Medical	Yes	Central	977	CCN12	Non-clade 4	Other	Diploid	-	-	1,2,3,R	-	-	2	2/S	0.12/S	0.25/WT	0.25/WT	0.06/S	0.03/S	0.015/S	1/WT	0.12	
441	F9500	13H1294	SRR23915252	2013	M	72	ICU	No	Northeast	10110	CCN103	Non-clade 4	Other	Diploid	-	chr 3-pos 3-0.5×	1,2,3,4,5,R	-	-	2	2/S	0.12/S	0.12/WT	0.12/WT	0.06/S	0.03/S	0.25/S	0.5/WT	≤0.06	
442	F3774	16LR315	SRR23915315	2016	M	76	ICU	Yes	Northeast	10060	CCN104	Non-clade 4	Other	Diploid	-	-	-	-	-	2	0.5/S	0.06/S	0.12/WT	0.12/WT	0.06/S	0.06/S	0.06/S	0.5/WT	0.06	
443	F3940	13J2020	SRR23915468	2013	M	40	Medical	Yes	East	10080	CCN105	Non-clade 4	Other	Diploid	-	chr 6-pos 1-2×	1,5,6,R	-	-	2	2/S	0.12/S	0.12/WT	0.12/WT	0.03/S	0.03/S	0.03/S	1/WT	0.06	
444	F3188	15H4210	SRR23915332	2015	M	83	Medical	Yes	Northeast	10006	CCN4	Non-clade 4	Other	Diploid	-	-	1,3,4,6,R	-	-	2	0.5/S	0.008/S	0.12/WT	0.03/WT	0.12/S	0.015/S	0.015/S	0.5/WT	0.12	
445	F9499	13H1279	SRR23915253	2013	F	48	Medical	Yes	Northeast	160	CCN4	Non-clade 4	Other	Diploid	-	-	1,4,6,R	-	-	2	1/S	0.12/S	0.12/WT	0.12/WT	0.06/S	0.03/S	0.25/S	0.5/WT	0.12	
446	F9498	13H1276	SRR23915437	2013	M	48	Medical	Yes	Northeast	1351	CCN4	Non-clade 4	Other	Diploid	-	-	1,2,6,R	-	-	2	0.5/S	0.03/S	0.12/WT	0.12/WT	0.03/S	0.015/S	0.12/S	0.25/WT	0.12	
447	F4038	15A1081	SRR23915585	2015	F	23	Medical	Yes	Northwest	10088	CCN4	Non-clade 4	Other	Diploid	-	chr 4-pos 1-1.5×	1,R	-	-	2	16/R	1/R	8/NWT	8/NWT	0.06/S	0.03/S	0.12/S	0.5/WT	0.12	
448	3137	21SJ807	SRR38060855	2021	M	34	Surgical	Yes	Northeast	10006	CCN4	Non-clade 4	Other	Diploid	-	-	1,3,4,6,R	-	-	2	2/S	0.12/S	0.25/WT	0.25/NWT	0.12/S	0.06/S	0.25/S	1/WT	0.12	Yes
449	F3854	15FJ438	SRR23915394	2015	F	71	Surgical	Yes	East	1351	CCN4	Non-clade 4	Other	Diploid	-	-	1,2,R	-	-	2	2/S	0.25/I	0.5/WT	0.5/NWT	0.06/S	0.03/S	0.12/S	0.5/WT	0.06	
450	F9501	13H1300	SRR23915251	2013	M	44	Medical	Yes	Northeast	1351	CCN4	Non-clade 4	Other	Diploid	-	-	1,2,4,5,6,R	-	-	2	2/S	0.12/S	0.12/WT	0.12/WT	0.12/S	0.03/S	0.25/S	1/WT	0.06	
451	F3546	15JD085	SRR23915660	2015	M	26	Medical	Yes	Northeast	10006	CCN4	Non-clade 4	Other	Diploid	-	-	1,2,3,4,6,R	-	-	2	2/S	0.25/I	0.25/WT	0.5/NWT	0.06/S	0.06/S	0.12/S	1/WT	0.25	
452	F3690	14JD038	SRR23915410	2014	M	72	Surgical	No	Northeast	10006	CCN4	Non-clade 4	Other	Diploid	-	-	1,2,3,4,6,R	-	-	2	2/S	0.25/I	0.25/WT	0.5/NWT	0.06/S	0.06/S	0.12/S	0.5/WT	0.12	
453	F3956	15JD109	SRR23915300	2015	F	60	Surgical	No	Northeast	10006	CCN4	Non-clade 4	Other	Diploid	-	-	1,2,3,4,6,R	-	-	2	4/SDD	0.5/I	0.5/WT	0.5/NWT	0.03/S	0.06/S	0.06/S	0.5/WT	0.25	
454	F3678	14Z2106	SRR23915415	2014	F	45	ICU	No	East	10053	CCN4	Non-clade 4	Other	Diploid	-	-	1,6,R	-	-	2	0.5/S	0.03/S	0.12/WT	0.06/WT	0.12/S	0.03/S	0.12/S	0.5/WT	0.06	
455	F4104	12NX080	SRR23915573	2012	M	62	Surgical	No	Northwest	10096	CCN4	Non-clade 4	Other	Diploid	-	-	1,2,6,R	-	-	2	1/S	0.06/S	0.12/WT	0.12/WT	0.03/S	0.03/S	0.015/S	1/WT	0.06	
456	F3830	16SJ251	SRR23915614	2016	F	83	ICU	Yes	Northeast	10069	CCN4	Non-clade 4	Other	Diploid	-	-	1,2,3,4,6,R	-	-	2	2/S	0.25/I	0.25/WT	0.25/WT	0.06/S	0.03/S	0.06/S	0.25/WT	0.06	
457	3092	21SJ762	SRR38060854	2021	M	64	ICU	No	Northeast	160	CCN4	Non-clade 4	Other	Diploid	-	-	1,3,4,5,R	-	-	2	4/SDD	0.25/I	0.25/WT	0.25/NWT	0.06/S	0.06/S	0.12/S	1/WT	≤0.06	Yes
458	3065	21SJ735	SRR38060852	2021	M	38	ICU	Yes	Northeast	160	CCN4	Non-clade 4	Other	Diploid	-	-	1,3,4,5,R	-	-	2	16/R	0.5/I	0.5/WT	0.5/NWT	0.015/S	0.03/S	0.12/S	1/WT	≤0.06	Yes
459	5049	21GZ1481	SRR38060851	2021	M	57	Medical	Yes	South	1381	CCN10	Non-clade 4	AZR-ADJ	Diploid	-	-	1,2,4,R	Y132F,S154F	Homozygous	2	64/R	2/R	0.5/WT	0.5/NWT	0.06/S	0.06/S	0.12/S	1/WT	≤0.06	Yes
460	5004	21GZ1436	SRR38060850	2021	M	77	Surgical	Yes	South	12018	CCN59	Non-clade 4	AZR-ADJ	Diploid	-	-	-	Y132F,F145L,S154F	Heterozygous	2	16/R	0.5/I	0.12/WT	0.12/WT	0.06/S	0.03/S	0.12/S	0.5/WT	0.12	Yes
461	3090	21SJ760	SRR38060849	2021	M	15	Medical	Yes	Northeast	959	CCN48	Non-clade 4	AZR-ADJ	Diploid	-	-	4,R	F145L	Heterozygous	2	16/R	1/R	0.5/WT	1/NWT	0.06/S	0.06/S	0.5/I	1/WT	0.12	Yes
462	F3376	15FJ464	SRR23915453	2015	M	21	Medical	Yes	East	10012	CCN106	Non-clade 4	Other	Diploid	-	-	1,R	-	-	2	2/S	0.25/I	0.5/WT	0.5/NWT	0.06/S	0.06/S	0.12/S	0.25/WT	0.06	
463	F3352	16DL078	SRR23915285	2016	M	79	ICU	Yes	Northeast	10012	CCN106	Non-clade 4	Other	Diploid	-	-	R	-	-	2	2/S	0.25/I	0.25/WT	0.5/NWT	0.12/S	0.06/S	0.12/S	1/WT	0.06	
464	F9492	11H1183	SRR23915444	2011	M	69	ICU	Yes	Northeast	10107	CCN111	Non-clade 4	AZR-ADJ	Diploid	-	-	5	Y132F, S154F	Homozygous	2	128/R	4/R	0.5/WT	0.5/NWT	0.03/S	0.03/S	0.015/S	0.25/WT	≤0.06	
465	F9491	11H1175	SRR23915445	2011	M	48	ICU	Yes	Northeast	10107	CCN111	Non-clade 4	AZR-ADJ	Diploid	-	-	5	Y132F, S154F	Homozygous	2	128/R	2/R	0.5/WT	0.5/NWT	0.06/S	0.03/S	0.03/S	1/WT	≤0.06	
466	F3888	15F1086	SRR23915376	2015	M	28	ICU	Yes	East	10074	CCN108	Non-clade 4	AZR-ADJ	Diploid	-	-	R	Y132F, S154F	Homozygous	2	64/R	4/R	0.5/WT	0.5/NWT	0.03/S	0.06/S	0.12/S	1/WT	0.06	
467	F9486	09H1016	SRR23915449	2009	M	20	ICU	Yes	Northeast	10074	CCN108	Non-clade 4	AZR-ADJ	Diploid	-	-	2,6	Y132F, S154F	Homozygous	2	64/R	2/R	0.5/WT	0.5/NWT	0.03/S	0.03/S	0.03/S	1/WT	≤0.06	
468	F9485	09H1015	SRR23915450	2009	M	24	ICU	No	Northeast	10074	CCN108	Non-clade 4	AZR-ADJ	Diploid	-	-	1,2	Y132F, S154F	Homozygous	2	64/R	2/R	0.5/WT	0.5/NWT	0.03/S	0.03/S	0.03/S	1/WT	≤0.06	
469	F9509	10HX096	SRR23915242	2010	M	42	Outpatient/Emergency	No	Southwest	10111	CCN109	Non-clade 4	AZR-ADJ	Diploid	-	-	1,2	Y132F, S154F	Heterozygous	2	>256/R	8/R	1/NWT	1/NWT	0.06/S	0.03/S	0.12/S	0.5/WT	≤0.06	
470	F9484	13BD301	SRR23915451	2012	M	88	Outpatient/Emergency	Yes	North	521	CCN9	Non-clade 4	AZR-ADJ	Diploid	-	-	1,6,R	Y132F, S154F	Heterozygous	2	32/R	2/R	0.5/WT	0.5/NWT	0.03/S	0.03/S	0.25/S	0.5/WT	0.12	
471	F4090	15HX811	SRR23915580	2015	M	57	ICU	Yes	Southwest	521	CCN9	Non-clade 4	AZR-ADJ	Diploid	-	-	-	Y132F, S154F	Heterozygous	2	16/R	0.5/I	0.25/WT	0.12/WT	0.03/S	0.03/S	0.12/S	1/WT	0.12	
472	F3824	15Z2136	SRR23943251	2015	M	68	ICU	Yes	East	329	CCN110	Non-clade 4	AZR-ADJ	Diploid	-	-	2,R	-	-	2	2/S	0.12/S	0.25/WT	0.25/WT	0.12/S	0.03/S	0.12/S	1/WT	≤0.06	
473	F3520	14DL017	SRR23943253	2014	F	50	Medical	Yes	Northeast	329	CCN110	Non-clade 4	AZR-ADJ	Diploid	-	-	2,5,6,R	Y132F, S154F	Homozygous	2	256/R	8/R	1/NWT	1/NWT	0.25/S	0.03/S	0.12/S	1/WT	0.06	
474	F9496	12H1241	SRR23915439	2012	F	11	Medical	Yes	Northeast	329	CCN110	Non-clade 4	AZR-ADJ	Diploid	-	-	5,6,R	Y132F, S154F	Homozygous	2	32/R	2/R	0.25/WT	0.25/WT	0.06/S	0.03/S	0.25/S	1/WT	≤0.06	
475	F9489	10H1092	SRR23915447	2010	M	67	ICU	Yes	Northeast	329	CCN110	Non-clade 4	AZR-ADJ	Diploid	-	-	5,6,R	Y132F, S154F	Homozygous	2	64/R	2/R	0.25/WT	0.25/WT	0.03/S	0.015/S	0.015/S	1/WT	≤0.06	
476	F3162	15H1435	SRR23915548	2015	F	47	ICU	Yes	Northeast	329	CCN110	Non-clade 4	AZR-ADJ	Diploid	-	-	5,6,R	Y132F, S154F	Homozygous	2	64/R	1/R	0.12/WT	0.25/WT	0.03/S	0.03/S	0.03/S	1/WT	0.06	
477	3586	21XH586	SRR38060848	2021	F	55	Surgical	No	Central	12002	CCN50	Non-clade 4	AZR-ADJ	Diploid	-	chr 1-pos 3-0.5×	R	Y132F,S154F	Heterozygous	2	64/R	2/R	0.5/WT	0.5/NWT	0.03/S	0.06/S	0.12/S	1/WT	≤0.06	Yes
478	3031	21SJ701	SRR38060847	2021	M	17	Medical	Yes	Northeast	508	CCN9	4	AZR-ADJ	Diploid	-	-	-	Y132F,S154F	Homozygous	2	64/R	2/R	0.25/WT	0.25/NWT	0.06/S	0.06/S	0.12/S	1/WT	≤0.06	Yes
479	F3348	14SJ118	SRR23915467	2014	M	57	ICU	No	Northeast	508	CCN9	4	AZR-ADJ	Diploid	-	-	R	Y132F, S154F	Heterozygous	2	16/R	1/R	0.25/WT	0.12/WT	0.03/S	0.03/S	0.03/S	1/WT	0.06	
480	F9482	13BD291	SRR23915454	2012	M	64	Surgical	Yes	North	508	CCN9	4	AZR-ADJ	Diploid	-	-	6,R	Y132F, S154F	Heterozygous	2	16/R	1/R	0.25/WT	0.12/WT	0.03/S	0.03/S	0.12/S	1/WT	≤0.06	
481	F3706	11S1004	SRR23915401	2011	M	85	Medical	Yes	East	335	CCN9	4	AZR-ADJ	Diploid	-	-	3,5,R	Y132F, S154F	Homozygous	2	64/R	2/R	0.25/WT	0.25/WT	0.06/S	0.03/S	0.03/S	0.25/WT	0.06	
482	F3500	15FJ465	SRR23915427	2015	M	20	Medical	Yes	East	10033	CCN9	4	AZR-ADJ	Diploid	-	-	-	Y132F, S154F	Heterozygous	2	16/R	1/R	0.25/WT	0.25/WT	0.06/S	0.06/S	0.12/S	0.5/WT	0.06	
483	F3742	16ZD427	SRR23915491	2016	M	33	Surgical	Yes	East	508	CCN9	4	AZR-ADJ	Diploid	-	-	4,R	Y132F, S154F	Heterozygous	2	32/R	2/R	0.25/WT	0.25/WT	0.06/S	0.03/S	0.03/S	1/WT	0.06	
484	F7114	13PU618	SRR23915357	2013	M	60	Outpatient/Emergency	No	North	508	CCN9	4	AZR-ADJ	Diploid	-	-	5,R	Y132F, S154F	Homozygous	2	>256/R	>8/R	0.5/WT	0.5/NWT	0.25/S	0.03/S	0.25/S	1/WT	0.25	
485	F7113	13PU614	SRR23915358	2013	M	60	Outpatient/Emergency	No	North	508	CCN9	4	AZR-ADJ	Diploid	-	-	5,R	Y132F, S154F	Homozygous	2	>256/R	>8/R	0.5/WT	0.5/NWT	0.12/S	0.03/S	0.25/S	1/WT	0.12	
486	Z650	21FS828	SRR38060846	2021	F	56	ICU	Yes	East	508	CCN9	4	AZR-ADJ	Diploid	-	-	2,R	Y132F,S154F	Heterozygous	2	8/R	0.5/I	0.12/WT	0.12/WT	0.03/S	0.06/S	0.25/S	2/WT	≤0.06	Yes
487	F3924	16PU1084	SRR23915475	2016	M	36	Surgical	Yes	North	508	CCN9	4	AZR-ADJ	Diploid	-	-	2	Y132F, S154F	Heterozygous	2	4/SDD	0.25/I	0.25/WT	0.12/WT	0.06/S	0.03/S	0.12/S	1/WT	≤0.06	
488	F9507	10HX043	SRR23915244	2010	F	55	ICU	Yes	Southwest	508	CCN9	4	AZR-ADJ	Diploid	-	-	1,R	Y132F, S154F	Heterozygous	2	16/R	0.5/I	0.25/WT	0.12/WT	0.03/S	0.015/S	0.015/S	1/WT	≤0.06	
489	F3912	15CQ235	SRR23915479	2015	M	60	ICU	No	Southwest	508	CCN9	4	AZR-ADJ	Diploid	-	-	3,5	Y132F, S154F	Homozygous	2	32/R	2/R	0.25/WT	0.12/WT	0.03/S	0.015/S	0.12/S	0.5/WT	0.06	
490	F3428	15TJ708	SRR23915533	2015	F	47	Surgical	Yes	Central	615	CCN107	Non-clade 4	AZR-ADJ	Diploid	-	-	1,3,R	Y132F, S154F	Heterozygous	2	256/R	8/R	16/NWT	8/NWT	0.06/S	0.06/S	0.25/S	1/WT	0.06	
491	F9521	12HX526	SRR23915556	2012	F	62	Medical	Yes	Southwest	615	CCN107	Non-clade 4	AZR-ADJ	Diploid	-	-	R	Y132F, S154F	Heterozygous	2	32/R	2/R	0.5/WT	0.5/NWT	0.03/S	0.03/S	0.12/S	0.5/WT	0.12	
492	F3330	14HX771	SRR23915324	2014	M	21	Medical	Yes	Southwest	615	CCN107	Non-clade 4	AZR-ADJ	Diploid	-	-	R	Y132F, S154F	Heterozygous	2	16/R	1/R	0.25/WT	0.25/WT	0.03/S	0.03/S	0.03/S	1/WT	0.06	
493	Z3692	21XH692	SRR38060845	2021	M	67	Surgical	No	Central	12005	CCN39	4	AZR	Diploid	-	-	3	Y132F,S154F	Heterozygous	7	>256/R	>8/R	0.5/WT	0.5/NWT	0.12/S	0.12/S	0.25/S	1/WT	≤0.06	Yes
494	Z3677	21XH677	SRR38060844	2021	M	67	Surgical	No	Central	12005	CCN39	4	AZR	Diploid	-	-	3	Y132F,S154F	Heterozygous	6	>256/R	>8/R	1/NWT	1/NWT	0.12/S	0.06/S	0.25/S	1/WT	0.12	Yes
495	Z3675	21XH675	SRR38060843	2021	M	67	Surgical	No	Central	12005	CCN39	4	AZR	Diploid	-	-	3	Y132F,S154F	Heterozygous	6	256/R	8/R	0.5/WT	1/NWT	0.12/S	0.06/S	0.25/S	1/WT	≤0.06	Yes
496	Z2995	20SJ664	SRR38060841	2020	M	43	Surgical	Yes	Northeast	12036	CCN37	4	AZR	Tetraploid	-	-	-	Y132F,S154F	Heterozygous	3	256/R	8/R	0.12/WT	1/NWT	0.12/S	0.03/S	0.12/S	0.5/WT	≤0.06	Yes
497	Z3005	20SJ674	SRR38060840	2020	M	44	ICU	Yes	Northeast	12006	CCN38	4	AZR	Diploid	-	-	2	Y132F,S154F	Heterozygous	5	256/R	8/R	0.5/WT	1/NWT	0.03/S	0.03/S	0.12/S	1/WT	≤0.06	Yes
498	Z3002	20SJ671	SRR38060758	2020	M	44	ICU	Yes	Northeast	12006	CCN38	4	AZR	Diploid	-	-	2	Y132F,S154F	Heterozygous	4	256/R	8/R	0.5/WT	1/NWT	0.03/S	0.06/S	0.12/S	2/WT	≤0.06	Yes
499	Z2996	20SJ665	SRR38060757	2020	M	43	ICU	Yes	Northeast	12006	CCN38	4	AZR	Diploid	-	-	2	Y132F,S154F	Heterozygous	4	256/R	8/R	0.5/WT	1/NWT	0.03/S	0.03/S	0.12/S	1/WT	≤0.06	Yes
500	4873	21GZ1305	SRR38060756	2021	M	79	ICU	No	South	12010	CCN0	4	AZR	Diploid	-	-	1,3,6	Y132F,S154F	Heterozygous	7	256/R	8/R	0.5/WT	0.5/NWT	0.06/S	0.06/S	0.12/S	0.5/WT	≤0.06	Yes
501	Z2726	20QQ027	SRR38060755	2020	F	74	Surgical	No	East	12014	CCN35	4	AZR	Tetraploid	-	-	-	Y132F,S154F	Heterozygous	5	256/R	8/R	1/NWT	1/NWT	0.03/S	0.06/S	0.12/S	1/WT	≤0.06	Yes
502	F3514	15GX074	SRR23915676	2015	F	53	Medical	Yes	South	10036	CCN0	4	AZR	Diploid	-	-	1,3,R	Y132F, S154F	Heterozygous	3	256/R	8/R	1/NWT	1/NWT	0.06/S	0.03/S	0.12/S	0.5/WT	0.06	
503	F3512	15GX075	SRR23915422	2015	F	62	ICU	No	South	506	CCN0	4	AZR	Diploid	-	-	1,3,R	Y132F, S154F	Heterozygous	6	256/R	8/R	0.5/WT	0.5/NWT	0.03/S	0.03/S	0.12/S	1/WT	≤0.06	
504	F3790	15SS015	SRR23915307	2015	F	65	Medical	Yes	North	506	CCN0	4	AZR	Diploid	-	-	R	Y132F, S154F	Heterozygous	8	512/R	8/R	1/NWT	1/NWT	0.03/S	0.03/S	0.06/S	1/WT	0.06	
505	F4082	16H4226	SRR23915258	2016	F	58	Surgical	Yes	Northeast	506	CCN0	4	AZR	Diploid	-	-	R	Y132F, S154F	Heterozygous	3	128/R	4/R	1/NWT	0.5/NWT	0.12/S	0.06/S	0.12/S	0.5/WT	0.06	
506	F3904	14XJ089	SRR23915482	2014	F	19	Medical	Yes	Northwest	506	CCN0	4	AZR	Diploid	-	-	R	Y132F, S154F, P397S	Heterozygous	8	512/R	8/R	1/NWT	1/NWT	0.03/S	0.03/S	0.03/S	2/WT	0.06	
507	F3600	14TJ530	SRR23915514	2014	M	53	Medical	Yes	Central	506	CCN0	4	AZR	Diploid	-	-	-	Y132F, S154F	Heterozygous	7	512/R	8/R	0.5/WT	0.5/NWT	0.03/S	0.03/S	0.12/S	1/WT	0.06	
508	F3782	15TJ680	SRR23915310	2015	F	18	Medical	Yes	Central	506	CCN0	4	AZR	Diploid	-	-	2,R	Y132F, S154F, P397S	Heterozygous	8	512/R	8/R	1/NWT	2/NWT	0.06/S	0.03/S	0.12/S	2/WT	0.06	
509	F3858	16DL099	SRR23915392	2016	M	51	Outpatient/Emergency	No	Northeast	225	CCN0	4	AZR	Diploid	-	-	2,3	Y132F, S154F	Heterozygous	7	512/R	8/R	0.5/WT	1/NWT	0.03/S	0.06/S	0.12/S	0.5/WT	0.06	
510	F9478	13BD263	SRR23915458	2013	N/A	N/A	ICU	No	North	506	CCN0	4	AZR	Diploid	-	-	6	Y132F, S154F	Heterozygous	7	512/R	>8/R	1/NWT	1/NWT	0.03/S	0.03/S	0.12/S	1/WT	≤0.06	
511	F3334	15HB048	SRR23915302	2015	F	62	Medical	Yes	North	506	CCN0	4	AZR	Diploid	-	-	R	Y132F, S154F	Heterozygous	8	512/R	>8/R	0.25/WT	0.5/NWT	0.015/S	0.03/S	0.03/S	1/WT	0.06	
512	F3964	15JS519	SRR23915294	2015	M	61	Surgical	No	East	754	CCN0	4	AZR	Diploid	-	-	2,R	Y132F, S154F	Heterozygous	7	512/R	>8/R	1/NWT	0.5/NWT	0.03/S	0.03/S	0.12/S	1/WT	≤0.06	
513	F3672	16GY018	SRR23915419	2016	F	45	Medical	Yes	Southwest	754	CCN0	4	AZR	Diploid	-	-	2	Y132F, S154F	Heterozygous	5	256/R	8/R	1/NWT	1/NWT	0.25/S	0.06/S	0.12/S	1/WT	0.06	
514	F3794	14J3066	SRR23915305	2014	N/A	N/A	Medical	No	East	506	CCN0	4	AZR	Diploid	-	-	1,6,R	Y132F, S154F	Heterozygous	7	512/R	8/R	1/NWT	1/NWT	0.03/S	0.03/S	0.03/S	1/WT	0.06	
515	F3826	16SS021	SRR23915616	2016	F	59	Medical	Yes	North	10068	CCN0	4	AZR	Diploid	-	-	6,R	Y132F, S154F	Heterozygous	4	128/R	8/R	0.25/WT	0.5/NWT	0.06/S	0.03/S	0.06/S	1/WT	0.06	
516	F4158	16ZD433	SRR23915362	2016	M	21	Medical	Yes	East	600	CCN0	4	AZR	Diploid	-	-	3,6,R	Y132F, S154F	Heterozygous	7	512/R	8/R	1/NWT	1/NWT	0.06/S	0.03/S	0.06/S	2/WT	0.06	
517	F3728	16ZD439	SRR23915500	2016	M	60	Surgical	Yes	East	506	CCN0	4	AZR	Diploid	-	-	1,2,6	Y132F, S154F, P397S	Heterozygous	5	256/R	8/R	1/NWT	1/NWT	0.06/S	0.03/S	0.06/S	1/WT	0.06	
518	5036	21GZ1468	SRR38060754	2021	F	39	Surgical	No	South	225	CCN0	4	AZR	Diploid	-	-	R	Y132F,S154F	Heterozygous	7	256/R	>8/R	0.5/WT	1/NWT	0.06/S	0.06/S	0.12/S	1/WT	≤0.06	Yes
519	F3582	16HX915	SRR23943252	2016	M	49	ICU	Yes	Southwest	506	CCN0	4	AZR	Diploid	-	-	R	Y132F, S154F	Heterozygous	8	512/R	>8/R	1/NWT	1/NWT	0.03/S	0.03/S	0.06/S	1/WT	≤0.06	
520	F3584	16HX921	SRR23915522	2016	M	49	ICU	Yes	Southwest	506	CCN0	4	AZR	Diploid	-	-	R	Y132F, S154F	Heterozygous	8	512/R	8/R	1/NWT	1/NWT	0.03/S	0.06/S	0.12/S	0.5/WT	0.06	
521	F3972	15NX150	SRR23915290	2015	M	21	Surgical	No	Northwest	506	CCN0	4	AZR	Diploid	-	-	R	Y132F, S154F	Heterozygous	8	4/SDD	0.25/I	0.25/WT	0.25/WT	0.06/S	0.03/S	0.06/S	1/WT	≤0.06	
522	F3622	14GX033	SRR23915649	2014	M	27	Surgical	Yes	South	754	CCN0	4	AZR	Diploid	-	-	2,6,R	Y132F, S154F	Heterozygous	5	256/R	8/R	1/NWT	1/NWT	0.03/S	0.03/S	0.06/S	1/WT	0.06	
523	F3900	14XJ087	SRR23915484	2014	M	47	Medical	Yes	Northwest	506	CCN0	4	AZR	Diploid	-	-	R	Y132F, S154F	Heterozygous	10	512/R	8/R	1/NWT	2/NWT	0.03/S	0.03/S	0.06/S	2/WT	0.06	
524	F3870	15HX846	SRR23915385	2015	F	49	Other wards	No	Southwest	506	CCN0	4	AZR	Diploid	-	-	R	Y132F, S154F	Heterozygous	10	512/R	8/R	1/NWT	1/NWT	0.25/S	0.06/S	0.25/S	1/WT	0.06	
525	F3946	15HZ053	SRR23915464	2015	M	66	ICU	Yes	Central	506	CCN0	4	AZR	Diploid	-	-	-	Y132F, S154F	Heterozygous	5	256/R	8/R	0.5/WT	0.5/NWT	0.12/S	0.03/S	0.12/S	1/WT	0.06	
526	F3984	15HZ046	SRR23915283	2015	F	32	Medical	No	Central	506	CCN0	4	AZR	Diploid	-	-	-	Y132F, S154F	Heterozygous	5	256/R	8/R	0.5/WT	1/NWT	0.06/S	0.03/S	0.12/S	1/WT	0.06	
527	F3358	16HB069	SRR23943248	2016	M	36	Medical	Yes	North	506	CCN0	4	AZR	Diploid	-	-	5	Y132F, S154F	Homozygous	6	256/R	8/R	1/NWT	1/NWT	0.03/S	0.03/S	0.06/S	1/WT	0.06	
528	F3902	14XJ084	SRR23915483	2014	M	29	Medical	Yes	Northwest	506	CCN0	4	AZR	Diploid	-	-	1,R	Y132F, S154F	Heterozygous	9	512/R	8/R	1/NWT	2/NWT	0.03/S	0.03/S	0.03/S	2/WT	0.06	
529	F3390	16FJ473	SRR23915550	2016	M	27	Medical	Yes	East	225	CCN0	4	AZR	Diploid	-	-	1,R	Y132F, S154F	Heterozygous	7	256/R	8/R	1/NWT	1/NWT	0.06/S	0.06/S	0.12/S	1/WT	0.06	
530	Z2721	20QQ022	SRR38060753	2020	F	74	Surgical	No	East	225	CCN0	4	AZR	Diploid	-	-	1,3,R	Y132F,S154F	Heterozygous	7	256/R	8/R	0.5/WT	1/NWT	0.06/S	0.06/S	0.25/S	1/WT	≤0.06	Yes
531	F3756	15A1045	SRR23915325	2015	M	86	ICU	No	Northwest	546	CCN0	4	AZR	Diploid	-	-	3,6	Y132F, S154F	Heterozygous	8	256/R	8/R	1/NWT	1/NWT	0.03/S	0.03/S	0.03/S	1/WT	0.06	
532	F3828	15LR267	SRR23915615	2015	M	88	ICU	Yes	Northeast	10066	CCN0	4	AZR	Diploid	-	-	1,3,6	Y132F, S154F	Heterozygous	7	256/R	8/R	1/NWT	1/NWT	0.03/S	0.03/S	0.015/S	1/WT	0.06	
533	F3820	15Z2137	SRR23915620	2015	M	68	ICU	No	East	10066	CCN0	4	AZR	Diploid	-	-	1,3,5,6	Y132F, S154F	Homozygous	10	4/SDD	0.25/I	0.25/WT	0.25/WT	0.03/S	0.03/S	≤0.015/S	0.25/WT	≤0.06	
534	F4026	14LR197	SRR23915592	2014	M	85	Surgical	Yes	Northeast	10066	CCN0	4	AZR	Diploid	-	-	1,3,6	Y132F, S154F	Heterozygous	6	256/R	8/R	1/NWT	1/NWT	0.015/S	0.015/S	0.015/S	1/WT	0.06	
535	F3996	15LR263	SRR23915604	2015	M	88	ICU	Yes	Northeast	10066	CCN0	4	AZR	Diploid	-	-	1,3,6	Y132F, S154F	Heterozygous	9	256/R	8/R	1/NWT	1/NWT	0.03/S	0.03/S	0.015/S	1/WT	0.06	
536	F3876	15LR264	SRR23915383	2015	M	88	ICU	Yes	Northeast	10066	CCN0	4	AZR	Diploid	-	-	1,3,5,6	Y132F, S154F	Homozygous	13	512/R	8/R	2/NWT	2/NWT	0.06/S	0.03/S	0.015/S	1/WT	0.06	
537	3018	21SJ688	SRR38060752	2021	F	32	Medical	Yes	Northeast	546	CCN0	4	AZR	Diploid	-	-	1,3,R	Y132F,S154F	Heterozygous	7	>256/R	>8/R	1/NWT	1/NWT	0.06/S	0.06/S	0.12/S	2/WT	≤0.06	Yes
538	A3681	23XH831	SRR38060751	2023	M	53	Medical	Yes	Central	546	CCN0	4	AZR	Diploid	-	-	1,3,R	Y132F,S154F	Heterozygous	8	>256/R	>8/R	0.5/WT	0.5/NWT	2/R	1/R	0.5/I	1/WT	≤0.06	
539	3716	21XH716	SRR38060749	2021	M	49	Surgical	No	Central	546	CCN0	4	AZR	Diploid	-	-	1,3,5,R	Y132F,S154F	Homozygous	12	>256/R	>8/R	0.5/WT	0.5/NWT	0.5/I	0.12/S	0.5/I	0.5/WT	≤0.06	Yes
540	3633	21XH633	SRR38060748	2021	F	46	Medical	Yes	Central	546	CCN0	4	AZR	Diploid	-	-	1,2,3,R	Y132F,S154F	Heterozygous	7	>256/R	>8/R	1/NWT	1/NWT	0.06/S	0.06/S	0.12/S	1/WT	≤0.06	Yes
541	3637	21XH637	SRR38060747	2021	F	54	Surgical	Yes	Central	225	CCN0	4	AZR	Diploid	-	-	R	Y132F,S154F	Heterozygous	8	>256/R	>8/R	1/NWT	1/NWT	0.06/S	0.06/S	0.25/S	1/WT	≤0.06	Yes
542	3642	21XH642	SRR38060746	2021	M	58	Medical	Yes	Central	225	CCN0	4	AZR	Diploid	-	-	5,R	Y132F,S154F	Homozygous	4	256/R	>8/R	0.5/WT	0.5/NWT	0.03/S	0.06/S	0.12/S	1/WT	≤0.06	Yes
543	4983	21GZ1415	SRR38060745	2021	M	64	Surgical	Yes	South	225	CCN0	4	AZR	Diploid	-	-	R	Y132F,S154F	Heterozygous	8	>256/R	8/R	0.5/WT	0.5/NWT	0.06/S	0.03/S	0.12/S	0.5/WT	≤0.06	Yes
544	3034	21SJ704	SRR38060744	2021	F	68	Medical	Yes	Northeast	225	CCN0	4	AZR	Diploid	-	-	R	Y132F,S154F	Heterozygous	3	256/R	8/R	0.5/WT	1/NWT	0.06/S	0.06/S	0.25/S	2/WT	≤0.06	Yes
545	3686	21XH686	SRR38060743	2021	F	65	Surgical	No	Central	225	CCN0	4	AZR	Diploid	-	-	R	Y132F,S154F	Heterozygous	8	>256/R	>8/R	1/NWT	1/NWT	0.12/S	0.06/S	0.12/S	1/WT	≤0.06	Yes
546	3674	21XH674	SRR38060742	2021	M	40	Surgical	Yes	Central	225	CCN0	4	AZR	Diploid	-	-	R	Y132F,S154F	Heterozygous	8	>256/R	>8/R	1/NWT	1/NWT	0.12/S	0.06/S	0.25/S	2/WT	≤0.06	Yes
547	L5716	22X1183	SRR38060741	2022	F	10	ICU	Yes	Northwest	225	CCN0	4	AZR	Diploid	-	-	R	Y132F,S154F	Heterozygous	7	>256/R	>8/R	1/NWT	1/NWT	0.25/S	0.06/S	0.12/S	2/WT	≤0.06	Yes
548	L3720	23XH870	SRR38060740	2023	M	56	Surgical	No	Central	225	CCN0	4	AZR	Diploid	-	-	R	Y132F,S154F	Heterozygous	8	>256/R	>8/R	1/NWT	1/NWT	0.12/S	0.06/S	0.25/S	2/WT	≤0.06	Yes
549	F4150	14Z2101	SRR23915367	2014	M	44	ICU	Yes	East	506	CCN0	4	AZR	Diploid	-	-	-	Y132F, S154F	Heterozygous	6	256/R	4/R	0.25/WT	0.5/NWT	0.06/S	0.03/S	0.06/S	0.5/WT	0.06	
550	F3504	15FJ463	SRR23943247	2015	M	68	Medical	Yes	East	506	CCN0	4	AZR	Diploid	-	-	5	Y132F, S154F	Homozygous	12	512/R	8/R	1/NWT	1/NWT	0.06/S	0.03/S	0.25/S	0.5/WT	0.06	
551	Z4333	21QH677	SRR38060738	2021	M	67	Surgical	Yes	East	225	CCN0	4	AZR	Diploid	-	-	-	Y132F,S154F	Heterozygous	7	256/R	>8/R	1/NWT	1/NWT	0.03/S	0.06/S	0.12/S	1/WT	≤0.06	Yes
552	F9522	12HX596	SRR23915555	2013	F	49	ICU	Yes	Southwest	595	CCN0	4	AZR	Diploid	-	-	5	Y132F, S154F	Heterozygous	8	512/R	>8/R	1/NWT	1/NWT	0.03/S	0.03/S	0.12/S	0.5/WT	≤0.06	
553	F4044	15DL066	SRR23915582	2015	F	89	Outpatient/Emergency	No	Northeast	225	CCN0	4	AZR	Diploid	-	-	5	Y132F, S154F	Heterozygous	5	256/R	8/R	0.5/WT	0.5/NWT	0.25/S	0.06/S	0.12/S	0.5/WT	0.06	
554	F3862	16DL089	SRR23915390	2016	M	88	Outpatient/Emergency	Yes	Northeast	225	CCN0	4	AZR	Diploid	-	chr 4-pos 2-2×	5	Y132F, S154F	Homozygous	8	512/R	8/R	1/NWT	1/NWT	0.06/S	0.03/S	0.12/S	1/WT	0.06	
555	F4156	15ZD406	SRR23943249	2015	M	63	Surgical	Yes	East	225	CCN0	4	AZR	Diploid	-	chr 4-pos 2-2.5×	1,5,R	Y132F, S154F	Heterozygous	16	512/R	8/R	1/NWT	1/NWT	0.06/S	0.06/S	0.25/S	1/WT	0.06	
556	F3714	14G1221	SRR23915508	2014	M	60	Medical	Yes	South	225	CCN0	4	AZR	Diploid	-	-	-	Y132F, S154F	Heterozygous	2	64/R	4/R	0.5/WT	1/NWT	0.03/S	0.03/S	0.06/S	1/WT	0.06	
557	F3654	14J3067	SRR23915631	2014	N/A	N/A	Medical	Yes	East	225	CCN0	4	AZR	Diploid	-	-	R	Y132F, S154F	Heterozygous	7	512/R	8/R	1/NWT	1/NWT	0.03/S	0.03/S	0.03/S	1/WT	0.06	
558	F3372	14HX752	SRR23915365	2014	M	37	ICU	Yes	Southwest	506	CCN0	4	AZR	Diploid	-	-	-	Y132F, S154F	Heterozygous	6	256/R	8/R	0.25/WT	0.25/WT	0.015/S	0.03/S	0.03/S	1/WT	0.06	
559	F9518	12HX445	SRR23915232	2013	F	55	Other wards	Yes	Southwest	506	CCN0	4	AZR	Diploid	-	-	4,R	Y132F, S154F	Heterozygous	7	512/R	8/R	1/NWT	1/NWT	0.06/S	0.03/S	0.12/S	0.5/WT	≤0.06	
560	F3516	15GX071	SRR23915675	2015	F	4	Surgical	Yes	South	506	CCN0	4	AZR	Diploid	-	-	R	Y132F, S154F	Heterozygous	8	512/R	8/R	1/NWT	1/NWT	0.03/S	0.03/S	0.12/S	0.5/WT	0.06	
561	F3548	15JD093	SRR23915659	2015	F	18	Medical	Yes	Northeast	506	CCN0	4	AZR	Diploid	-	-	R	Y132F, S154F	Heterozygous	8	512/R	8/R	1/NWT	1/NWT	0.03/S	0.03/S	0.25/S	1/WT	0.06	
562	F3676	15HX790	SRR23915416	2015	M	17	Medical	Yes	Southwest	506	CCN0	4	AZR	Diploid	chr 5-1.5×	-	-	Y132F, S154F	Heterozygous	7	512/R	8/R	0.25/WT	0.5/NWT	0.03/S	0.03/S	0.03/S	1/WT	0.06	
563	F3560	15JS379	SRR23915532	2015	M	18	Medical	Yes	East	506	CCN0	4	AZR	Diploid	-	-	-	Y132F, S154F	Heterozygous	8	1024/R	>8/R	1/NWT	1/NWT	0.06/S	0.03/S	0.12/S	1/WT	≤0.06	
564	F3866	15HX864	SRR23915387	2015	M	61	Medical	No	Southwest	506	CCN0	4	AZR	Diploid	-	-	4,R	Y132F, S154F	Heterozygous	8	512/R	8/R	0.5/WT	0.5/NWT	0.25/S	0.06/S	0.12/S	0.5/WT	0.06	
565	F3750	15A1040	SRR23915487	2015	M	48	Surgical	No	Northwest	225	CCN0	4	AZR	Diploid	-	-	4,R	Y132F, S154F	Heterozygous	10	512/R	8/R	1/NWT	1/NWT	0.03/S	0.03/S	0.12/S	1/WT	0.06	
566	F3532	15JS396	SRR23915668	2015	F	59	Medical	No	East	225	CCN0	4	AZR	Diploid	-	-	4,5,R	Y132F, S154F	Heterozygous	8	512/R	8/R	0.5/WT	0.5/NWT	0.03/S	0.03/S	0.03/S	1/WT	0.06	
567	F3528	15JS395	SRR23915670	2015	F	63	Medical	Yes	East	225	CCN0	4	AZR	Diploid	-	-	4,5,R	Y132F, S154F	Heterozygous	8	256/R	8/R	0.5/WT	0.5/NWT	0.03/S	0.03/S	0.12/S	0.5/WT	0.06	
568	F3534	15JS403	SRR23915667	2015	M	43	Medical	No	East	225	CCN0	4	AZR	Diploid	-	-	4,5,R	Y132F, S154F	Heterozygous	8	512/R	8/R	0.5/WT	0.5/NWT	0.03/S	0.03/S	0.06/S	1/WT	0.06	
569	F3958	15JS373	SRR23915299	2015	F	65	Medical	Yes	East	225	CCN0	4	AZR	Diploid	-	-	4,5,R	Y132F, S154F	Heterozygous	6	512/R	>8/R	0.5/WT	0.5/NWT	0.03/S	0.03/S	0.015/S	0.12/WT	0.06	
570	F3540	15JS405	SRR23915663	2015	F	59	Surgical	No	East	225	CCN0	4	AZR	Diploid	-	chr 4-pos 1-3×	1,4,5,R	Y132F, S154F	Heterozygous	6	256/R	8/R	0.5/WT	0.5/NWT	0.03/S	0.03/S	0.06/S	1/WT	0.06	
571	F3570	15PU983	SRR23915528	2015	M	46	Outpatient/Emergency	Yes	North	506	CCN0	4	AZR	Diploid	-	-	-	Y132F, S154F	Heterozygous	6	256/R	8/R	1/NWT	1/NWT	0.015/S	0.03/S	0.03/S	1/WT	0.06	
572	F3344	14ZD326	SRR23915378	2014	F	45	Medical	Yes	East	506	CCN0	4	AZR	Diploid	-	-	-	Y132F, S154F, P397S	Heterozygous	6	256/R	8/R	0.5/WT	0.5/NWT	0.03/S	0.03/S	0.06/S	1/WT	0.06	
573	F3394	16GH594	SRR23915547	2016	M	88	ICU	Yes	North	506	CCN0	4	AZR	Diploid	-	-	2,3	Y132F, S154F	Heterozygous	4	256/R	8/R	0.5/WT	0.5/NWT	0.03/S	0.03/S	0.12/S	1/WT	0.06	
574	Z3144	21SJ814	SRR38060737	2021	M	99	Medical	Yes	Northeast	225	CCN0	4	AZR	Diploid	-	-	2,R	Y132F,S154F	Heterozygous	5	>256/R	>8/R	1/NWT	1/NWT	0.12/S	0.06/S	0.12/S	1/WT	0.12	Yes
575	Z3140	21SJ810	SRR38060736	2021	M	99	Medical	Yes	Northeast	225	CCN0	4	AZR	Diploid	-	-	2,R	Y132F,S154F	Heterozygous	6	>256/R	>8/R	1/NWT	1/NWT	0.12/S	0.06/S	0.12/S	2/WT	≤0.06	Yes
576	Z3135	21SJ805	SRR38060735	2021	M	99	Medical	Yes	Northeast	225	CCN0	4	AZR	Diploid	-	-	2,R	Y132F,S154F	Heterozygous	5	>256/R	>8/R	1/NWT	1/NWT	0.06/S	0.06/S	0.12/S	1/WT	≤0.06	Yes
577	F3580	16HX939	SRR23915525	2016	F	64	Surgical	No	Southwest	506	CCN0	4	AZR	Diploid	-	-	-	Y132F, S154F	Heterozygous	8	256/R	8/R	0.5/WT	1/NWT	0.12/S	0.06/S	0.12/S	0.5/WT	0.06	
578	F3328	15TJ743	SRR23915494	2015	M	5	Other wards	Yes	Central	506	CCN0	4	AZR	Diploid	-	-	3	Y132F, S154F	Heterozygous	7	512/R	8/R	0.5/WT	1/NWT	0.06/S	0.03/S	0.25/S	1/WT	0.06	
579	F4116	15PU1021	SRR23915566	2015	F	69	Surgical	No	North	225	CCN0	4	AZR	Diploid	-	-	R	Y132F, S154F	Heterozygous	7	512/R	8/R	1/NWT	2/NWT	0.03/S	0.03/S	0.06/S	2/WT	0.06	
580	F4020	15PU1042	SRR23943250	2015	F	69	Surgical	No	North	225	CCN0	4	AZR	Diploid	-	-	R	Y132F, S154F	Heterozygous	7	512/R	8/R	1/NWT	1/NWT	0.03/S	0.06/S	0.06/S	1/WT	0.06	
581	F3544	15JS414	SRR23915661	2015	M	74	Medical	Yes	East	506	CCN0	4	AZR	Diploid	-	-	-	Y132F, S154F	Heterozygous	8	512/R	8/R	0.5/WT	0.5/NWT	0.03/S	0.03/S	0.03/S	1/WT	0.06	
582	F4112	15PU1019	SRR23915568	2015	F	69	Surgical	No	North	225	CCN0	4	AZR	Diploid	-	-	6,R	Y132F, S154F	Heterozygous	8	512/R	8/R	2/NWT	1/NWT	0.5/I	0.03/S	0.12/S	1/WT	0.06	
583	F3592	15GX101	SRR23915518	2015	M	58	Medical	Yes	South	506	CCN0	4	AZR	Diploid	Isochr 3q-1.5x	-	3,6,R	Y132F, S154F	Heterozygous	7	1024/R	>8/R	2/NWT	1/NWT	0.06/S	0.015/S	0.03/S	0.5/WT	≤0.06	
584	F3578	16HX933	SRR23915526	2016	M	47	ICU	Yes	Southwest	506	CCN0	4	AZR	Diploid	-	-	6,R	Y132F, S154F	Heterozygous	8	512/R	8/R	0.5/WT	0.5/NWT	0.06/S	0.06/S	0.12/S	0.5/WT	0.06	
585	F9495	12H1222	SRR23915440	2012	M	60	ICU	Yes	Northeast	10109	CCN0	4	AZR	Diploid	-	-	1,2,3,R	Y132F, S154F	Heterozygous	6	256/R	8/R	0.5/WT	0.5/NWT	0.03/S	0.03/S	0.25/S	1/WT	≤0.06	
586	4838	21GZ1270	SRR38060734	2021	M	50	Surgical	No	South	225	CCN0	4	AZR	Diploid	-	-	3,6,R	Y132F,S154F	Heterozygous	6	256/R	4/R	0.25/WT	0.25/NWT	0.06/S	0.03/S	0.06/S	0.5/WT	≤0.06	Yes
587	F3374	14HX753	SRR23915353	2014	F	65	Medical	Yes	Southwest	225	CCN0	4	AZR	Diploid	-	-	4	Y132F, S154F	Heterozygous	8	512/R	8/R	0.5/WT	0.5/NWT	0.03/S	0.03/S	0.03/S	1/WT	0.06	
588	F3932	16PU1131	SRR23915472	2016	F	61	ICU	No	North	225	CCN0	4	AZR	Diploid	-	-	-	Y132F, S154F	Heterozygous	7	512/R	8/R	1/NWT	1/NWT	0.03/S	0.03/S	0.015/S	1/WT	0.06	
589	F3812	15K1048	SRR23915624	2015	M	36	Medical	Yes	Southwest	506	CCN0	4	AZR	Diploid	-	-	R	Y132F, S154F	Heterozygous	8	512/R	8/R	0.5/WT	0.5/NWT	0.03/S	0.03/S	0.015/S	1/WT	0.06	
590	F3804	14DZ015	SRR23915627	2014	F	82	ICU	Yes	Northeast	225	CCN0	4	AZR	Diploid	-	-	-	Y132F, S154F	Heterozygous	8	512/R	8/R	0.5/WT	1/NWT	0.12/S	0.03/S	0.25/S	0.5/WT	0.06	
591	Z379	20CQ488	SRR38060733	2020	F	75	Surgical	No	Southwest	849	CCN0	4	AZR	Diploid	-	-	2,R	Y132F,S154F	Heterozygous	6	256/R	8/R	0.5/WT	1/NWT	0.06/S	0.03/S	0.25/S	1/WT	≤0.06	Yes
592	F9474	10BD113	SRR23915351	2010	F	21	ICU	No	North	225	CCN0	4	AZR	Diploid	-	-	6	Y132F, S154F	Heterozygous	2	32/R	1/R	0.5/WT	0.5/NWT	0.03/S	0.03/S	0.12/S	0.5/WT	≤0.06	
593	F4076	16CH122	SRR23915261	2016	F	64	Medical	No	East	225	CCN0	4	AZR	Diploid	-	-	R	Y132F, S154F	Heterozygous	7	512/R	8/R	1/NWT	1/NWT	0.06/S	0.06/S	0.12/S	1/WT	0.06	
594	F3526	15FJ425	SRR23915671	2015	M	29	Medical	Yes	East	506	CCN0	4	AZR	Diploid	-	-	-	Y132F, S154F	Heterozygous	8	512/R	8/R	1/NWT	2/NWT	0.06/S	0.06/S	0.12/S	0.5/WT	0.06	
595	3708	21XH708	SRR38060732	2021	M	55	ICU	Yes	Central	506	CCN0	4	AZR	Diploid	-	-	-	Y132F,S154F	Heterozygous	4	256/R	8/R	0.5/WT	0.5/NWT	0.06/S	0.06/S	0.12/S	1/WT	≤0.06	Yes
596	3707	21XH707	SRR38060731	2021	M	49	Medical	Yes	Central	506	CCN0	4	AZR	Diploid	-	-	-	Y132F,S154F	Heterozygous	4	256/R	>8/R	1/NWT	1/NWT	0.06/S	0.03/S	0.12/S	1/WT	≤0.06	Yes
597	A408	23T1523	SRR38060730	2023	M	61	Medical	Yes	North	1318	CCN137	Non-clade 4	Other	Diploid	-	-	R	V125A	Heterozygous	2	8/R	1/R	0.5/WT	0.5/NWT	>8/R	4/R	1/R	1/WT	0.12	
598	4990	21GZ1422	SRR38060729	2021	F	59	Surgical	No	South	12003	CCN58	4	AZR	Diploid	-	-	-	Y132F,S154F	Heterozygous	4	>256/R	>8/R	>16/NWT	>8/NWT	0.03/S	0.03/S	0.03/S	1/WT	≤0.06	Yes
599	4937	21GZ1369	SRR38060727	2021	M	37	ICU	No	South	1507	CCN16	4	AZR	Diploid	-	-	-	Y132F,S154F	Heterozygous	5	256/R	8/R	0.5/WT	0.5/NWT	0.03/S	0.06/S	0.06/S	1/WT	≤0.06	Yes
600	Z3662	21XH662	SRR38060726	2021	M	52	Surgical	No	Central	1507	CCN16	4	AZR	Diploid	-	-	-	Y132F,S154F	Heterozygous	7	256/R	>8/R	0.5/WT	0.5/NWT	8/R	1/R	0.25/S	1/WT	≤0.06	Yes
601	Z3651	21XH651	SRR38060725	2021	M	52	Surgical	Yes	Central	12004	CCN16	4	AZR	Diploid	chr 5-1.5×	-	-	Y132F,S154F	Heterozygous	8	>256/R	>8/R	1/NWT	1/NWT	4/R	2/R	0.5/I	1/WT	≤0.06	Yes
602	Z3652	21XH652	SRR38060724	2021	M	52	Surgical	Yes	Central	1507	CCN16	4	AZR	Diploid	-	-	-	Y132F,S154F	Heterozygous	7	>256/R	>8/R	1/NWT	1/NWT	4/R	2/R	0.5/I	2/WT	0.12	Yes
603	Z3660	21XH660	SRR38060723	2021	M	52	Surgical	Yes	Central	1507	CCN16	4	AZR	Diploid	-	-	-	Y132F,S154F	Heterozygous	8	>256/R	>8/R	1/NWT	1/NWT	>8/R	1/R	1/R	1/WT	≤0.06	Yes
604	Z3650	21XH650	SRR38060722	2021	M	52	Surgical	No	Central	1507	CCN16	4	AZR	Diploid	-	-	-	Y132F,S154F	Heterozygous	7	>256/R	>8/R	1/NWT	1/NWT	2/R	2/R	1/R	1/WT	≤0.06	Yes

Abbreviations: WGS, whole genome sequencing; CTC, *Candida tropicalis* clusters; F, female; M, male; MLST, multilocus sequence typing; DST, diploid sequence type; CC, clonal complex; AZR, cluster AZR; ADJ, group AZR-ADJ; LOH, loss of heterozygosity; FLU, fluconazole; VOR, voriconazole; IZ, itraconazole; PZ, posaconazole; CAS, caspofungin; MF, micafungin; AND, anidulafungin; AMB, amphotericin B; FC, 5-flucytosine; I, intermediate; S, susceptible; SDD, susceptible dose-dependent; R, resistant; WT, wild-type; NWT, non-wild-type; MIC, minimum inhibitory concentration; SRA, NCBI sequence read archive (https://www.ncbi.nlm.nih.gov/sra).

Supplementary Table S2. CLSI clinical breakpoints (CBPs) and epidemiological cutoff values (ECVs) used for antifungal susceptibility interpretation.

Antifungal agent	MIC Interpretive Criteria (CBPs and ECVs), μg/mL				
	S	SDD	I	R	ECV
Fluconazole	≤2	4	-	≥8	-
Voriconazole	≤0.12	-	0.25–0.5	≥1	-
Itraconazole	-	-	-	-	0.5
Posaconazole	-	-	-	-	0.12
Caspofungin	≤0.25	-	0.5	≥1	-
Micafungin	≤0.25	-	0.5	≥1	-
Anidulafungin	≤0.25	-	0.5	≥1	-
Amphotericin B	-	-	-	-	2

Note: S, susceptible; SDD, susceptible dose-dependent; I, intermediate; R, resistant; CBP, clinical breakpoint; ECV, epidemiological cutoff value. ECVs were used for antifungal agents without CLSI clinical breakpoints.

Supplementary Table S3. Multivariable logistic regression analysis of factors associated with fluconazole resistance among *C. tropicalis* isolates.

Characteristics	OR (95% CI)	*p*-value
Specimen source		
Non-blood	1.00	
Blood	1.25 (0.93-1.68)	0.141
Geographic region		0.002
Northeast	1.00	
North	3.22 (1.66-6.24)	0.001
East	2.91 (1.55-5.45)	0.001
South	3.85 (1.91-7.72)	<0.001
Central	2.03 (1.03-4.01)	0.04
Northwest	3.24 (1.67-6.29)	0.001
Southwest	2.14 (1.06-4.34)	0.034
Clinical service		<0.001
Medical	1.00	
Outpatient/Emergency	0.54 (0.33-0.90)	0.019
ICU	0.40 (0.28-0.58)	<0.001
Other wards	0.60 (0.31-1.16)	0.127
Surgical	0.51 (0.34-0.75)	0.001

Note: OR, odds ratio; CI, confidence interval; ICU, intensive care unit. Variables with OR >1 indicate increased odds relative to the reference category, whereas OR <1 indicates decreased odds. Reference categories are indicated by OR = 1.00. *p*-values were calculated using multivariable logistic regression analysis.

Supplementary Table S4. Novel multilocus sequence typing (MLST) diploid sequence types (DSTs) identified in this study.

DST	MLST loci					
	ZWF1a	*MDR1*	*ICL1*	*SAPT2*	*SAPT4*	*XYR1*
12024	2002	9	1	22	17	60
12029	22	22	1	22	17	60
12030	7	17	1	12	17	48
12026	9	4	1	4	23	2005
12023	38	91	15	29	2006	1004
12001	4	1001	3	1	41	77
12001	4	1001	3	1	41	77
12008	3	45	1	3	36	48
12012	3	90	1	3	7	1003
12009	3	7	7	1	2003	72
12021	22	22	1	3	38	4
12015	7	240	5	1	7	23
12027	7	240	5	1	7	2006
12015	7	240	5	1	7	23
12034	3	122	1	1	7	58
12032	3	22	1	3	3	48
12033	5	20	2	1003	61	26
12031	22	2005	5	12	2007	69
12016	1	72	18	4	13	73
12019	4	7	62	1	1013	67
12019	4	7	62	1	1013	67
12022	3	53	1002	23	20	54
12011	3	45	1	3	11	48
12025	22	2004	1003	4	11	169
12013	9	169	9	1	3	80
12013	9	169	9	1	3	80
12013	9	169	9	1	3	80
12018	44	22	1	1	10	54
12002	3	90	9	22	7	9
12005	22	7	9	2001	10	48
12005	22	7	9	2001	10	48
12005	22	7	9	2001	10	48
12036	22	7	9	2	2001	240
12006	47	7	9	2	2001	119
12006	47	7	9	2	2001	119
12006	47	7	9	2	2001	119
12010	1	7	9	12	10	119
12014	22	22	9	2	134	48
12003	22	1001	9	1	10	119
12004	9	7	26	3	10	2001

Supplementary Table S5. Multivariable logistic regression analysis of factors associated with cluster AZR distribution among fluconazole-resistant *C. tropicalis* isolates.

Characteristics	OR (95% CI)	*p*-value
Specimen source		
Non-blood	1.00	
Blood	1.19 (0.57-2.45)	0.645
Geographic region		0.479
Northeast	1.00	
North	0.63 (0.21-1.83)	0.393
East	1.24 (0.43-3.58)	0.692
South	0.95 (0.29-3.09)	0.936
Central	1.84 (0.62-5.48)	0.272
Northwest	2.24 (0.38-13.39)	0.377
Southwest	0.68 (0.25-1.89)	0.464
Clinical service		0.09
Medical	1.00	
Outpatient/Emergency	0.32 (0.08-1.34)	0.118
ICU	0.45 (0.20-0.99)	0.047
Other wards	0.67 (0.12-3.94)	0.661
Surgical	1.24 (0.50-3.10)	0.646

Note: OR, odds ratio; CI, confidence interval; ICU, intensive care unit. Variables with OR >1 indicate increased odds relative to the reference category, whereas OR <1 indicates decreased odds. Reference categories are indicated by OR = 1.00. *p*-values were calculated using multivariable logistic regression analysis.

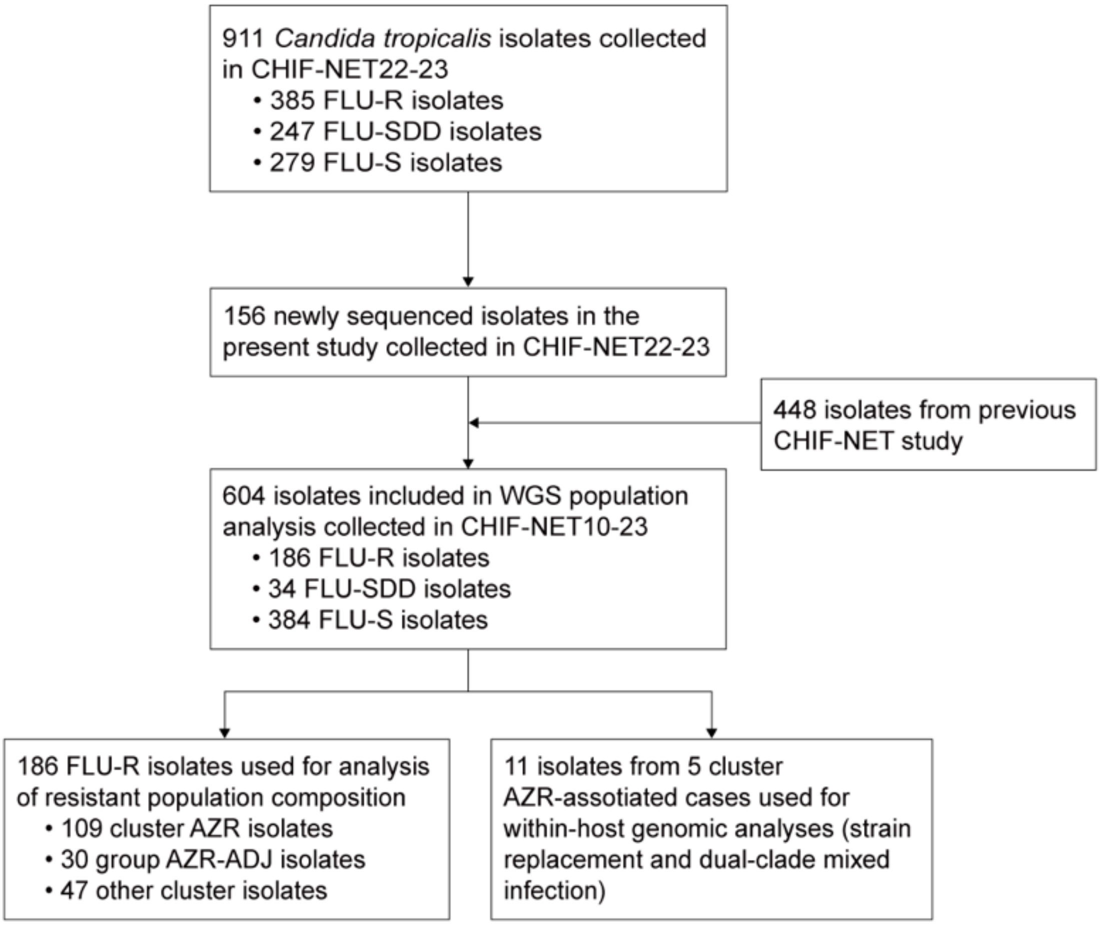

Supplementary Figure S1. Selection workflow of *Candida tropicalis* isolates included in the population genomic analysis.
